# Enhancers of the PAIR4 regulatory module promote distal V_H_ gene recombination at the *Igh* locus

**DOI:** 10.15252/embj.2022112741

**Published:** 2023-06-20

**Authors:** Louisa Hill, Markus Jaritz, Hiromi Tagoh, Karina Schindler, Daniela Kostanova‐Poliakova, Qiong Sun, Tanja A Schwickert, Martin Leeb, Meinrad Busslinger

**Affiliations:** ^1^ Research Institute of Molecular Pathology (IMP) Vienna BioCenter (VBC) Vienna Austria; ^2^ Max Perutz Laboratories University of Vienna, Vienna BioCenter (VBC) Vienna Austria

**Keywords:** CTCF, *Igh* V_H_ gene recombination, novel recombination enhancers, PAIR4 element, Pax5, Chromatin, Transcription & Genomics, Immunology

## Abstract

While extended loop extrusion across the entire *Igh* locus controls V_H_‐DJ_H_ recombination, local regulatory sequences, such as the PAIR elements, may also activate V_H_ gene recombination in pro‐B‐cells. Here, we show that PAIR‐associated V_H_8 genes contain a conserved putative regulatory element (V8E) in their downstream sequences. To investigate the function of PAIR4 and its V8.7E, we deleted 890 kb containing all 14 PAIRs in the *Igh* 5′ region, which reduced distal V_H_ gene recombination over a 100‐kb distance on either side of the deletion. Reconstitution by insertion of PAIR4‐V8.7E strongly activated distal V_H_ gene recombination. PAIR4 alone resulted in lower induction of recombination, indicating that PAIR4 and V8.7E function as one regulatory unit. The pro‐B‐cell‐specific activity of PAIR4 depends on CTCF, as mutation of its CTCF‐binding site led to sustained PAIR4 activity in pre‐B and immature B‐cells and to PAIR4 activation in T‐cells. Notably, insertion of V8.8E was sufficient to activate V_H_ gene recombination. Hence, enhancers of the PAIR4‐V8.7E module and V8.8E element activate distal V_H_ gene recombination and thus contribute to the diversification of the BCR repertoire in the context of loop extrusion.

## Graphical abstract

Local enhancers in the *Igh* locus promote V_H_ gene recombination and contribute to the diversification of the B cell receptor repertoire.

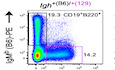

## Introduction

Humoral immunity to foreign pathogens depends on the generation of a diverse B cell antigen receptor (BCR) repertoire by V(D)J recombination, which assembles the variable regions of immunoglobulin (Ig) genes from variable (V), diversity (D) and joining (J) segments during B cell development (Jhunjhunwala *et al*, 2009; Schatz & Swanson, 2011; Alt *et al*, 2013). The Ig heavy‐chain (*Igh*) locus undergoes D_H_‐J_H_ rearrangements in lymphoid progenitors followed by V_H_‐DJ_H_ recombination in committed pro‐B cells (Jhunjhunwala *et al*, 2009; Alt *et al*, 2013). The *Igh* locus of the mouse is composed of a 0.26‐Mb long 3′ proximal region, containing the D_H_, J_H,_ and C_H_ gene segments, and of a distal 2.44‐Mb long V_H_ gene cluster (Johnston *et al*, 2006; Proudhon *et al*, 2015). Due to the large size of the V_H_ gene cluster, contraction of the entire *Igh* locus is required to juxtapose distantly located V_H_ genes next to the 3′ proximal DJ_H_‐rearranged gene segment, which facilitates V_H_‐DJ_H_ recombination in committed pro‐B cells (Kosak *et al*, 2002; Fuxa *et al*, 2004; Roldán *et al*, 2005; Jhunjhunwala *et al*, 2008; Medvedovic *et al*, 2013). The transcription factor Pax5 is known to regulate *Igh* locus contraction (Fuxa *et al*, 2004) by downregulating the expression of the cohesin‐release factor Wapl in pro‐B cells, which facilitates cohesin‐dependent chromatin loop extrusion across the entire *Igh* locus (Hill *et al*, 2020). Pax5 binding at the *Wapl* promoter recruits the Polycomb repressive complex 2 (PRC2), which leads to a 4‐fold repression of *Wapl* mRNA expression and, consequently, to an increased residence time of cohesin on DNA, thus resulting in the formation of extra‐long chromatin loops (Hill *et al*, 2020). Extended loop extrusion facilitates the convergent alignment of the recombination signal sequences (RSS) of all V_H_ genes with the RSS element of the DJ_H_‐rearranged segment in the 3′ proximal RAG^+^ recombination center prior to RAG‐mediated V_H_‐DJ_H_ recombination (Ji *et al*, 2010; Hill *et al*, 2020; Dai *et al*, 2021; Zhang *et al*, 2022).

Two regulatory elements have so far been shown to control essential aspects of V(D)J recombination at the *Igh* locus. The Eμ enhancer, which is located in the 3′ proximal region of the *Igh* locus, promotes D_H_‐J_H_ recombination in lymphoid progenitors by inducing active chromatin at the D_H_ and J_H_ clusters (Perlot *et al*, 2005; Chakraborty *et al*, 2009) and thereby contributes to the formation of the local RAG^+^ recombination center (Ji *et al*, 2010; Schatz & Ji, 2011). Moreover, two CTCF‐binding sites in the IGCR1 region, which is located between the V_H_ and D_H_ clusters, are essential for controlling the B‐lineage specificity of *Igh* recombination and the temporal order of D_H_‐J_H_ recombination prior to V_H_‐DJ_H_ recombination at the *Igh* locus (Guo *et al*, 2011).

We previously identified the Pax5‐activated intergenic repeat (PAIR) elements, which are dispersed over a 750‐kb region in the distal V_H_ gene cluster (Ebert *et al*, 2011). The 14 PAIR elements are defined by the presence of a highly conserved sequence repeat of 470‐bp length and are frequently associated with a member of the V_H_8 (V_H_3609) gene family (Ebert *et al*, 2011). The PAIR element and its associated V_H_8 gene contain active chromatin in pro‐B cells, suggesting that the two elements may function as a regulatory unit (Ebert *et al*, 2011). In addition, a long noncoding (lnc) RNA is transcribed from a promoter in the PAIR element (Ebert *et al*, 2011). PAIR4 and PAIR6 were subsequently identified as the most active PAIR elements as judged by the high transcription of their lncRNAs in pro‐B cells (Verma‐Gaur *et al*, 2012; Medvedovic *et al*, 2013). The activity of PAIR elements is strictly pro‐B cell‐specific, as their lncRNA is not expressed in pre‐B, immature B or T cells (Ebert *et al*, 2011). The transcription factors Pax5, E2A, CTCF, and YY1 are known to bind to PAIR elements (Ebert *et al*, 2011; Medvedovic *et al*, 2013). While the lncRNA expression from the promoters of PAIR4 and PAIR6 is lost in YY1‐deficient pro‐B cells (Verma‐Gaur *et al*, 2012; Medvedovic *et al*, 2013), it is increased upon downregulation of the CTCF protein (Degner *et al*, 2011). However, no genetic analysis has yet been performed to decipher the role of PAIR elements in the regulation of V_H_ gene recombination.

Here, we have analyzed the function of PAIR4 and its associated V_H_8‐7 gene by detailed genetic analysis. Mapping of open chromatin localized the putative regulatory element in the V_H_8‐7 gene region immediately downstream of the RSS sequence. We therefore refer to this element as the V_H_8‐7‐associated enhancer (V8.7E). Moreover, the presence of open chromatin downstream of the RSS element was identified as a specific feature of members of the V_H_8 gene family. Due to the high conservation of the PAIR sequences, we first eliminated all 14 PAIRs by deleting an 890‐kb long region in the distal V_H_ gene cluster, followed by insertion of different PAIR4 constructs at the deletion point. While the 890‐kb deletion resulted in reduced recombination of distal V_H_ genes over a 100‐kb distance on either side of the deletion point, the insertion of PAIR4 together with V8.7E strongly activated distal V_H_ gene recombination in pro‐B cells. Reconstitution with PAIR4 alone resulted in lower activation of V_H_ gene recombination, indicating that PAIR4 and V8.7E function as one regulatory unit. Pax5 contributed to the PAIR4 function as mutation of the Pax5‐binding site in PAIR4 resulted in decreased expression of the PAIR4‐derived transcript and reduced activation of V_H_ gene recombination. Notably, the pro‐B cell‐specific activity of PAIR4 was critically dependent on CTCF, as mutation of its CTCF‐binding site resulted in sustained PAIR4 activity in pre‐B and immature B cells as well as in the activation of PAIR4‐derived transcription in T cells. In summary, these data demonstrate that the enhancers of the PAIR4‐V8.7E module activate V_H_ gene recombination in the distal V_H_ gene region, which further contributes to the diversification of the BCR repertoire in the context of chromatin loop extrusion.

## Results

### Activation of PAIR4, PAIR6, and their downstream V_H_8‐associated elements during the BLP‐to‐pro‐B cell transition

**Figure 1:**
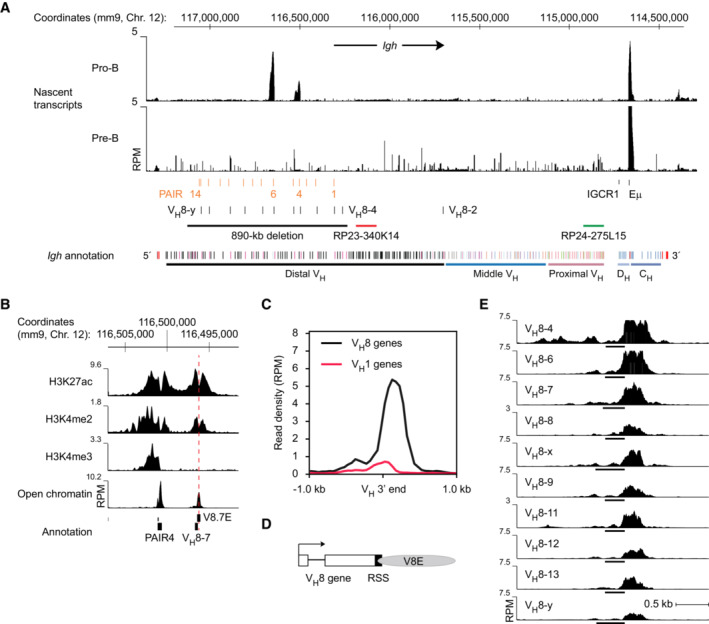
Identification of the V8E elements as a new class of potential regulatory elements in the distal V_H_ gene region of the *Igh* locus Mapping of total transcripts at the *Igh* locus in ex vivo sorted pro‐B and pre‐B cells. Total RNA was sequenced, as described in Materials and Methods, and plotted as reads per million mapped sequence reads (RPM). The locations of the PAIR elements, V_H_8 genes, IGCR1 sequence, and Eμ enhancer are shown together with the positions of the bacterial artificial chromosomes (BACs) RP23‐340K14 and RP24‐275L15 used as DNA‐FISH probes. The annotation of the C57BL/6 *Igh* locus indicates the distinct V_H_ gene families (different colors) in the distal, middle, and proximal V_H_ gene regions (Johnston *et al*, 2006), the D_H_ (light blue) and C_H_ (blue) elements, as well as the 3′RR enhancers (red) in the 3′ proximal *Igh* domain, together with the mm9 genomic coordinates of mouse chromosome 12. A black line indicates the extent of the 890‐kb deletion present in the *Igh* ^∆890^ allele.Presence of active histone marks and open chromatin at PAIR4 and the V8.7E element located immediately downstream of the V_H_8‐7 gene in pro‐B cells. *Ex vivo* sorted pro‐B cells were used for determining open chromatin by ATAC‐seq and short‐term cultured pro‐B cells for ChIP‐seq analysis of the indicated histone marks with modification‐specific antibodies. The peak of open chromatin at the V8.7E element is indicated by a dashed line.Specific presence of an open chromatin peak downstream of members of the V_H_8 gene family. The density of cumulative ATAC‐seq reads located downstream of members of the V_H_8 and V_H_1 gene families is shown. The 3′ end of the V_H_ genes was used as a reference point for sequence alignment.Schematic diagram of a V_H_8 gene with its RSS sequence and downstream V8E element. Gray shading indicates the extent of the ATAC‐seq peak present at the V8E element.Identification of different V8E elements by their open chromatin peaks that are located downstream of individual V_H_8 genes (indicated by black lines). As the previously identified V_H_3609.8pg.160 and V_H_3609.14pg.181 genes (Johnston *et al*, 2006) were not mapped in the mouse mm9 or mm10 genomes, we refer to these PAIR6‐ and PAIR13‐associated genes as V_H_8‐x and V_H_8‐y, respectively. These genes have the following coordinates: V_H_8‐x gene – chr12:116642631‐116643076 (mm9) or chr12:115404420‐115404865 (mm10); V_H_8‐y gene – chr12:117046639‐117047071 (mm9) or chr12:115808428‐115808860 (mm10).

**Figure EV1:**
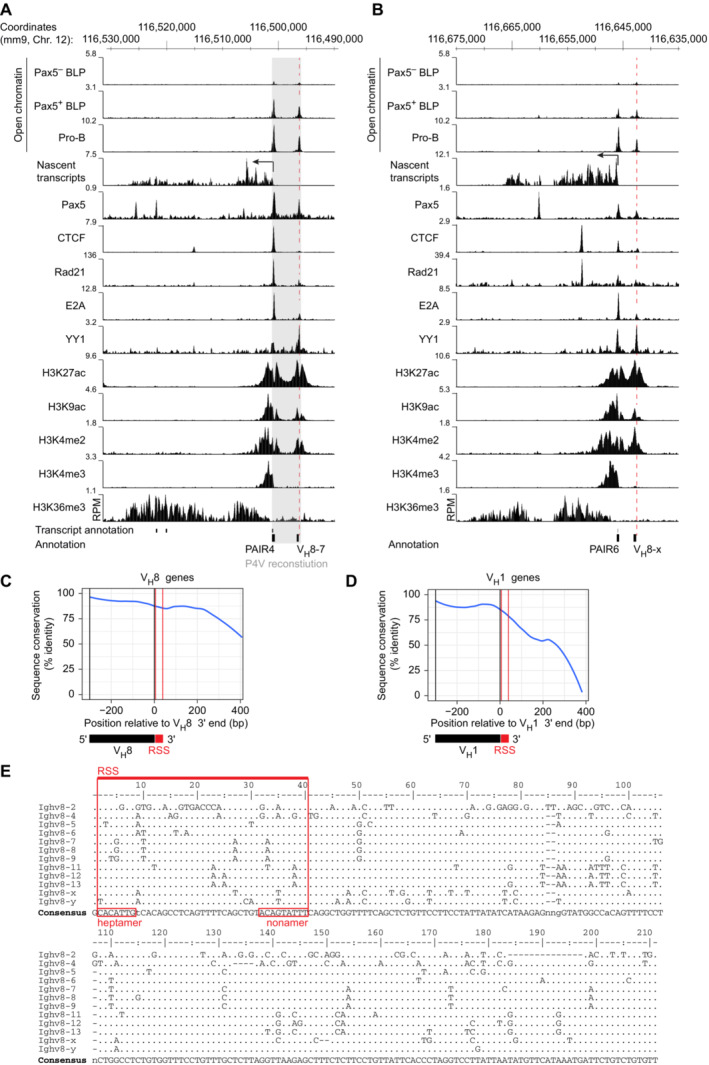
Characterization of the PAIR4, PAIR6, and V8E elements A, B
Presence of active histone marks, transcription factor binding, open chromatin, and transcription in the PAIR4 (A) and PAIR6 (B) regions at the pro‐B cell stage. Open chromatin was furthermore mapped by ATAC‐seq in hCD2^−^ (Pax5^−^) and hCD2^+^ (Pax5^+^) BLPs that were sorted from the bone marrow of *Pax5* ^ihCd2/ihCd2^ mice (Fuxa & Busslinger, 2007; Hill *et al*, 2020). The RPM scales for displaying the open chromatin peaks in BLPs and pro‐B cells were adjusted to show equal densities of the open chromatin peaks present at a gene‐dense genomic region, including the ubiquitously expressed *Tbp* locus. The location of PAIR4, PAIR6, and their associated V_H_8 genes are shown together with the exons of the PAIR4‐derived lncRNA (RIKEN clone CJ056205; below) and the mm9 genomic coordinates of mouse chromosome 12 (above). As the previously identified V_H_3609.8pg.160 (Johnston *et al*, 2006) was not mapped in the mouse mm9 or mm10 genome, we refer to this PAIR6‐associated gene as V_H_8‐x. The coordinates for the V_H_8‐x gene are chr12:116642631‐116643076 (mm9) or chr12:115404420–115,404,865 (mm10). Gray overlay indicates the DNA sequences of the PAIR4‐V8.7E module that were inserted with the Floxin method at the deletion point of the *Igh* ^∆890^ allele to generate the *Igh* ^P4V^ allele (Fig 3A). RPM, reads per million mapped sequence reads.C, D
Sequence conservation of the aligned V_H_8 (C) and V_H_1 (D) gene regions and their 3′ flanking sequences (~400 bp). The line denotes the LOESS‐smoothed position‐wise maximum sequence identity across the multiple sequence alignments. The coding sequences of the V_H_ genes are indicated together with the recombination signal sequences (RSS).E
Sequence alignment of the ~200‐bp conserved region downstream of the 3′ end of the V_H_8 genes. Dots indicate identical nucleotides relative to the consensus sequence shown below. Nonidentical nucleotides are shown by their respective letters (C, G, T, and A). Gaps in the alignment are indicated by dashes. Numbers refer to the positions downstream of the V_H_8 3′ end.

To study nascent transcription along the *Igh* locus, we performed total RNA‐sequencing of pro‐B and pre‐B cells that were sorted from the bone marrow of wild‐type mice. This analysis identified abundant transcripts in three regions of the *Igh* locus at the pro‐B cell stage. A high density of transcripts was detected in the Iμ‐Cμ region downstream of the Eμ enhancer (Fig 1A). The two other regions with strong transcription corresponded to the lncRNAs that originate from the PAIR4 and PAIR6 elements in the 5′ region of the *Igh* locus (Figs 1A and EV1A and B). These two PAIR‐associated transcripts were, however, already lost at the next developmental stage in pre‐B cells (Fig 1A). Hence, only these two of the 14 PAIR elements exhibited strong transcriptional activity in pro‐B cells (Fig 1A) in agreement with previous reports (Verma‐Gaur *et al*, 2012; Medvedovic *et al*, 2013). Consistent with this finding, both PAIR4 and PAIR6 contained the active histone marks H3K4me2, H3K4me3, and H3K27ac and were bound by Pax5, E2A, YY1, CTCF, and cohesin (Rad21) in pro‐B cells (Ebert *et al*, 2011; Figs 1B and EV1A and B). Active chromatin (H3K4me2 and H3K27ac) was also present at the adjacent V_H_8‐7 and V_H_8‐x genes downstream of PAIR4 and PAIR6, respectively, while the active promoter mark H3K4me3 was absent in pro‐B cells in agreement with the lack of nascent transcripts at these two V_H_ genes (Figs 1B and EV1A and B). Mapping of open chromatin by ATAC‐sequencing (Buenrostro *et al*, 2013) revealed that the putative regulatory element in the V_H_8‐7 and V_H_8‐x gene regions was located downstream of the RSS sequences of these V_H_ genes (Figs 1B and EV1A and B). Importantly, open chromatin could not be detected at the two PAIR and V_H_8‐associated elements in the majority of B‐cell‐biased lymphoid progenitors (Pax5^−^ BLPs), whereas open chromatin was already induced in the small fraction of Pax5^+^ BLPs (Fig EV1A and B). Hence, we conclude that PAIR4, PAIR6, and their downstream V_H_8‐associated elements are activated in the transition from BLPs to committed pro‐B cells.

Among all V_H_ genes, the members of the V_H_8 gene family predominantly contain active histone marks at a relatively high level in pro‐B cells (Malin *et al*, 2010). Moreover, 11 of the 14 PAIR elements are associated with a member of the V_H_8 gene family (Ebert *et al*, 2011), raising the question whether open chromatin peaks are located downstream of most of these genes. Cumulative analysis of the ATAC‐seq reads at all V_H_8 (V_H_3609) and V_H_1 (V_H_J558) genes present in the distal V_H_ gene cluster revealed that open chromatin was specifically present downstream of the V_H_8 genes in contrast to the V_H_1 genes (Fig 1C and D). Moreover, the analysis of individual V_H_8 genes confirmed the presence of open chromatin peaks downstream of most of these genes, although there was some variation in read density of these peaks (Fig 1E). Notably, the downstream sequences of the V_H_8 genes were strongly conserved, contrary to the respective sequences of the V_H_1 genes (Fig EV1C–E). We refer to these putative regulatory elements (V8E) by the number of their associated V_H_8 gene, and hence the element next to the V_H_8‐7 gene is named as V8.7E.

### An 890‐kb deletion in the *Igh* 5′ region impairs distal V_H_ gene recombination

**Figure 2:**
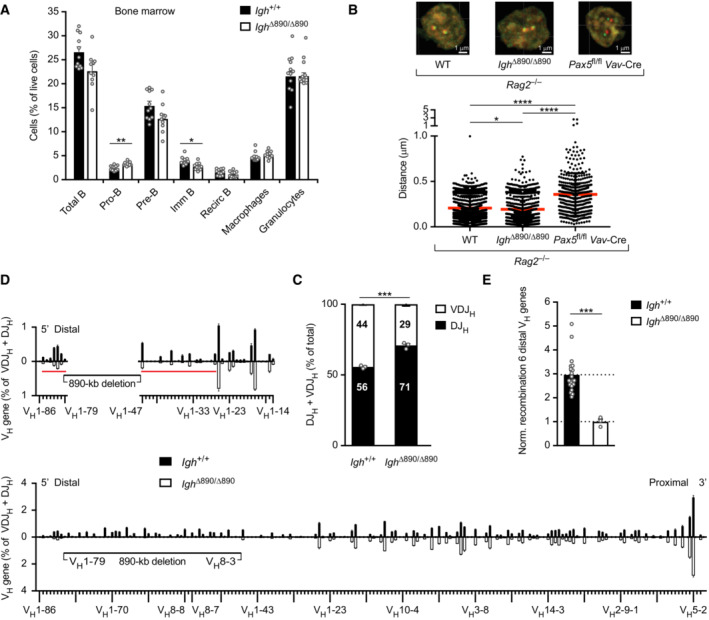
An 890‐kb deletion in the 5′ region of the *Igh* locus impairs adjacent distal V_H_ gene recombination A
B cell development in *Igh* ^∆890/∆890^ mice. The relative frequencies of the indicated cell types were determined by flow cytometric analysis of bone marrow cells from *Igh* ^∆890/∆890^ and *Igh* ^+/+^ mice at the age of 3–4 weeks, indicating a 1.3‐fold increase of pro‐B cells and a moderate decrease at subsequent B cell developmental stages in *Igh* ^∆890/∆890^ mice. Statistical data are shown as a mean value with SEM.B
Two‐color 3D DNA‐FISH analysis of ex vivo sorted pro‐B cells of the indicated genotypes with the RP23‐340K14 (red) and RP24‐275L15 (green) BAC probes (Fig 1A). Representative images are shown above. Dot plots (below) show the distances measured between the two DNA signals in individual *Igh* alleles (2,123 for *Rag2* ^−/−^, 1,514 for *Igh* ^∆890/∆890^ *Rag2* ^−/−^, and 502 for *Vav*‐Cre *Pax5* ^fl/fl^ (*Pax5* ^∆/∆^) *Rag2* ^−/−^ pro‐B cells) together with the mean distance (red bar) and SEM determined for each genotype. In total, four independent DNA‐FISH experiments were performed.C, D
V_H_ gene recombination analysis in ex vivo sorted *Igh* ^∆890/∆890^ and *Igh* ^+/+^ pro‐B cells, as determined by VDJ‐seq experiments (Chovanec *et al*, 2018). (C) The percentages of uniquely identified DJ_H_ and VDJ_H_ sequences are shown as mean percentages with SEM. Each dot corresponds to an independent VDJ‐seq experiment performed with sorted pro‐B cells from one mouse. (D) VDJ‐seq analysis of V_H_ gene rearrangements in *Igh* ^+/+^ pro‐B cells (black, above line) and *Igh* ^∆890/∆890^ pro‐B cells (white bars, below line). The different V_H_ genes (horizontal axis) are aligned according to their position in the *Igh* locus (Dataset EV1). The usage of each V_H_ gene (vertical axis) is shown as a percentage of all VDJ_H_ and DJ_H_ recombination events determined for each pro‐B cell type. The relative frequency of each V_H_ gene is shown as a mean value with SEM and is based on three independent VDJ‐seq experiments for pro‐B cells of each genotype. The enlargement (upper part) highlights the differences in recombination frequency of the V_H_ genes adjacent to the 890‐kb deletion. The red horizontal line indicates the extent of reduced V_H_ gene recombination on either side of the deletion point in *Igh* ^∆890/∆890^ pro‐B cells.E
Normalized recombination frequency of the first six distal V_H_ genes (V_H_1‐85 to V_H_1‐80) in *Igh* ^∆890/∆890^ and *Igh* ^+/+^ pro‐B cells. The average recombination frequency of the six distal V_H_ genes was calculated as mean value with SEM, and the value obtained with *Igh* ^∆890/∆890^ pro‐B cells was set to 1. The average recombination frequency in *Igh* ^+/+^ pro‐B cells was determined based on all VDJ‐seq experiments that were performed in this study with this control pro‐B cell type. The average recombination frequency of each distal V_H_ gene in *Igh* ^∆890/∆890^ and *Igh* ^+/+^ pro‐B cells is shown in Fig EV3C. Data information: Statistical data were analyzed by multiple *t*‐tests (unpaired and two‐tailed with Holm‐Sidak correction; A, C, E) or one‐way ANOVA (Tukey *post hoc* test; B); *P < 0.05, **P < 0.01, ***P < 0.001, ****P < 0.0001. Each dot (A, C, and E) corresponds to one mouse. Source data are available online for this figure.

**Figure EV2:**
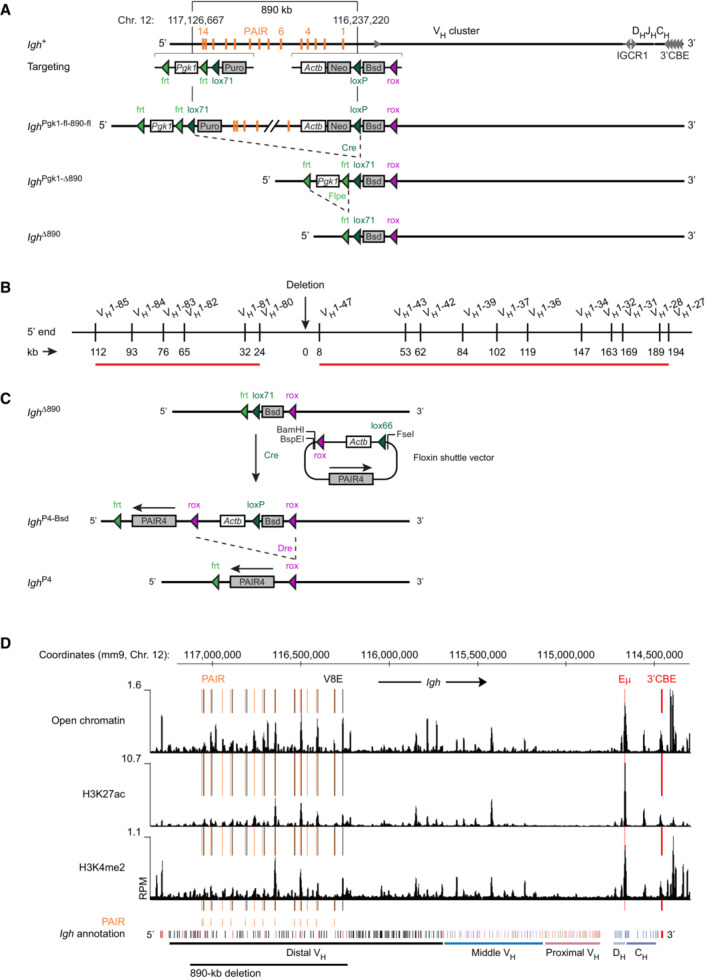
Generation, characterization, and reconstitution of the *Igh* ^∆890^ allele and presence of potential regulatory elements throughout the V_H_ gene cluster A
Generation of the *Igh* ^∆890^ allele. The indicated selection cassettes were used for introducing the upstream *lox*71 site and the downstream *lox*P site at the specified positions of the *Igh* locus by sequential ES cell targeting to generate the *Igh* ^Pgk1‐fl‐890‐fl^ allele. The *Igh* ^∆890^ allele was subsequently generated by sequential deletion of the indicated sequences by Cre‐ and Flpe‐mediated recombination *in vivo* in the mouse, as described in Materials and Methods. Actb, human β‐actin promoter; Bsd, blasticidin resistance gene; Neo, neomycin resistance gene; Puro, puromycin resistance gene; Pgk1, phosphoglycerate kinase‐1 promoter.B
Location of the V_H_1 gene family members, which undergo rearrangements in *Igh* ^+/+^ pro‐B cells and exhibit strongly reduced recombination (indicated by the red line; see Fig 2D) upon deletion of the 890‐kb region in the 5′ region of the *Igh* locus. The distance of the different V_H_ genes from the deletion point is shown in kilobases (kb).C
Schematic diagram of the Floxin system used for inserting the different PAIR4 constructs and the Eμ enhancer at the deletion point of the *Igh* ^∆890^ allele in ES cells. See Materials and Methods for detailed description of the reconstitution experiments.D
Presence of active chromatin across the *Igh* locus in *Rag2* ^−/−^ pro‐B cells. Open chromatin was mapped by ATAC‐seq, while the presence of H3K27ac and H3K4me2 peaks was identified by ChIP‐seq analysis with anti‐H3K27ac and anti‐H3K4me2 antibodies. The peaks corresponding to PAIR and V8E elements are highlighted by orange or black lines, respectively. The Eμ enhancer and 3′CBE region are indicated in red. The annotation of the C57BL/6 *Igh* locus with the different V_H_ gene families (different colors), the D_H_ (light blue), and C_H_ (blue) elements is shown together with the extent of the 890‐kb deletion (black line) and the mm9 genomic coordinates of mouse chromosome 12.

As the role of PAIR4 and PAIR6 in V_H_‐DJ_H_ recombination is still unknown, we decided to analyze the function of PAIR4 in detail. Due to the high sequence conservation of the 470‐bp long repeats defining the 14 PAIR elements (Ebert *et al*, 2011), we first deleted all 14 PAIR elements and then reconstituted the deletion by inserting wild‐type and mutant PAIR4 constructs. For this, we deleted an 890‐kb distal *Igh* region, containing 32 functional V_H_ genes in addition to the PAIR elements (Fig 1A), to generate the *Igh* ^∆890^ allele by sequential insertion of *lox*71 and *lox*P sites through ES cell targeting and subsequent Cre‐mediated deletion of the *lox*‐flanked sequences *in vivo* in the mouse (Fig EV2A and Materials and Methods). B cell development was moderately decreased at the pre‐B and immature B cell stage in the bone marrow of *Igh* ^∆890/∆890^ mice compared with control *Igh* ^+/+^ mice, which was likely caused by the observed increase of pro‐B cells in *Igh* ^∆890/∆890^ mice (Fig 2A). This partial B cell developmental arrest is likely brought about by the loss of the 32 functional V_H_ genes, which may reduce the overall frequency of V_H_‐DJ_H_ recombination in *Igh* ^∆890/∆890^ pro‐B cells (see below).

We next tested the hypothesis that the PAIR elements may contribute to *Igh* locus contraction in pro‐B cells (Ebert *et al*, 2011). To this end, we analyzed pro‐B cells by 3‐dimensional DNA‐fluorescence in situ hybridization (3D DNA‐FISH; Fig 2B) by using bacterial artificial chromosome (BAC) probes corresponding to central (red) or proximal (green) V_H_ gene regions (Fig 1A). For this, we used ex vivo sorted *Igh* ^∆890/∆890^ *Rag2* ^−/−^, *Vav*‐Cre *Pax5* ^fl/fl^ (*Pax5* ^∆/∆^) *Rag2* ^−/−^ and *Rag2* ^−/−^ pro‐B cells, which were unable to undergo V(D)J recombination due to RAG2 deficiency (Shinkai *et al*, 1992). While the two DNA‐FISH signals were far apart in the nuclei of *Pax5* ^∆/∆^ *Rag2* ^−/−^ pro‐B cells, they were overlapping in *Igh* ^∆890/∆890^ *Rag2* ^−/−^ pro‐B cells like in control *Rag2* ^−/−^ pro‐B cells (Fig 2B). These data therefore indicate that *Igh* locus contraction is not affected by deletion of all PAIR elements.

Analysis of V(D)J recombination by VDJ sequencing (VDJ‐seq; Chovanec *et al*, 2018) revealed proportionally fewer VDJ_H_‐rearranged *Igh* alleles in bone marrow pro‐B cells of *Igh* ^∆890/∆890^ mice compared with control *Igh* ^+/+^ mice, which may be largely caused by deletion of the 32 functional V_H_ genes (Fig 2C and D). Notably, the recombination of the V_H_ genes, which are located on either side of the deletion point, was strongly reduced in *Igh* ^∆890/∆890^ pro‐B cells compared with *Igh* ^+/+^ pro‐B cells (Fig 2D). V_H_ gene recombination was decreased over a distance of 112 kb (V_H_1‐85) or 194 kb (V_H_1‐27) upstream or downstream of the deletion point in *Igh* ^∆890/∆890^ pro‐B cells, respectively (Fig EV2B). To quantify the effect of the 890‐kb deletion on distal V_H_ gene recombination, we determined the average recombination frequency of the first six distal V_H_ genes (V_H_1‐85 to V_H_1‐80) in *Igh* ^∆890/∆890^ pro‐B cells relative to the corresponding frequency of these V_H_ genes in control *Igh* ^+/+^ pro‐B cells (Fig 2E). This analysis revealed that the 890‐kb deletion caused on average, a 3‐fold decrease in the recombination frequency of these distal V_H_ genes.

### PAIR4 with its associated V8.7E element strongly activates distal V_H_ gene recombination

**Figure 3:**
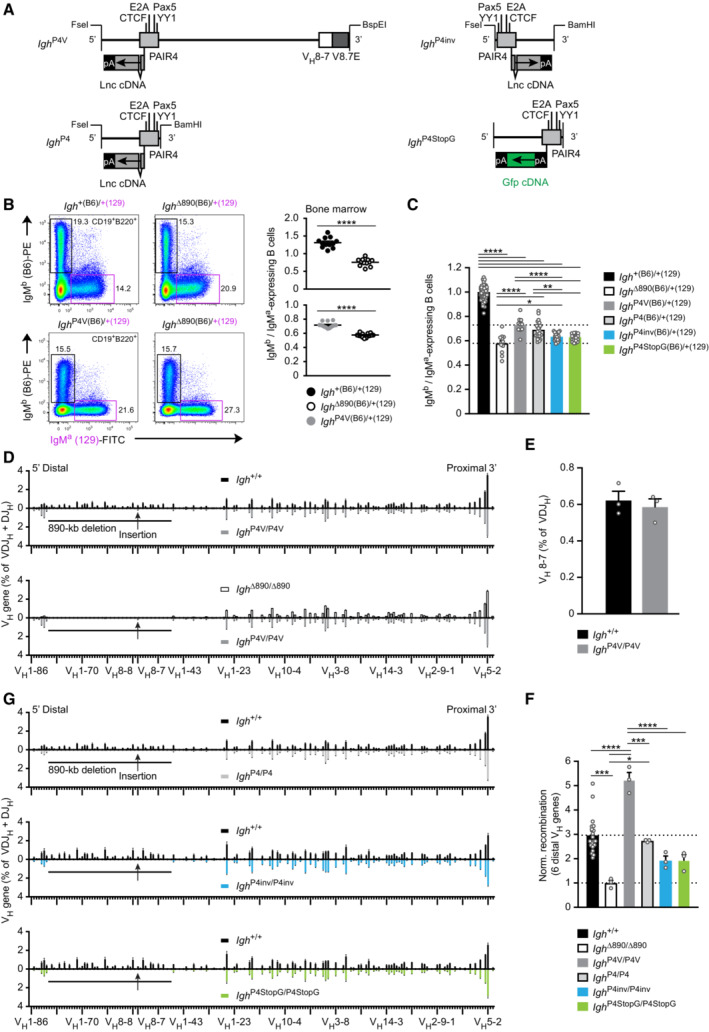
Identification of PAIR4‐V8.7E as a potent enhancer module promoting distal V_H_ gene recombination Schematic diagram of the different PAIR4 constructs used for reconstitution experiments. The PAIR4 element with its transcription factor binding sites and the cDNA insertion of the PAIR‐derived lncRNA (RIKEN clone CJ056205) in exon 2 of PAIR4 is shown together with the V_H_8‐7 gene and its downstream V8.7E element. Six copies of a synthetic polyadenylation sequence (pA; Levitt *et al*, 1989) linked to *Gfp* cDNA were inserted in exon 1 of PAIR4 to generate the P4StopG insert. FseI‐BspEI or FseI‐BamHI DNA fragments of the different PAIR4 constructs were inserted at the deletion point into the *Igh* ^∆890^ allele with the Floxin system (Singla *et al*, 2010) as described in detail in Fig EV2C and Materials and Methods. pA, 6 polyadenylation sites.Flow cytometric determination of the ratio of immature IgM^b^ (B6) to IgM^a^ (129) B cells in the bone marrow of *Igh* ^∆890(B6)/+(129)^, *Igh* ^P4V (B6)/+(129)^ and control *Igh* ^+(B6)/+(129)^ mice, which were generated by crossing *Igh* ^∆890/+^, *Igh* ^P4V/+^ and *Igh* ^+/+^ mice on the C57BL/6 (B6) background with *Igh* ^+/+^ mice of the 129/Sv (129) strain. The rearranged *Igh* alleles of the C57BL/6 and 129/Sv strains give rise to the expression of IgM^b^ and IgM^a^, respectively. The IgM^b^ (B6) to IgM^a^ (129) ratio is shown as a mean value with SEM (right).Summary of the ratios of immature IgM^b^ (B6) to IgM^a^ (129) B cells, determined for the indicated six mouse strains analyzed and shown as mean values with SEM. The ratio determined for control *Igh* ^+(B6)/+(129)^ B cells was set to 1.Comparison of the VDJ‐seq data obtained with pro‐B cells of the *Igh* ^+/+^ (black), *Igh* ^∆890/∆890^ (white bars), and *Igh* ^P4V/P4V^ (gray) genotypes. The different V_H_ genes (horizontal axis) are aligned according to their position in the *Igh* locus (Dataset EV1). The usage of each V_H_ gene (vertical axis) is shown as a percentage of all VDJ_H_ and DJ_H_ recombination events determined for each pro‐B cell type. The relative frequency of each V_H_ gene is shown as a mean value with SEM and is based on three independent VDJ‐seq experiments for pro‐B cells of each genotype.Recombination frequency of the inserted V_H_8‐7 gene, which was determined as a percentage of all VDJ_H_ recombination events in *Igh* ^+/+^ and *Igh* ^P4V/P4V^ pro‐B cells and is shown as a mean value with SEM.Normalized recombination frequency of the first six distal V_H_ genes (V_H_1‐85 to V_H_1‐80) determined in pro‐B cells of the indicated genotypes, based on the data shown in (D, G). The average recombination frequency of the six distal V_H_ genes was calculated as a mean value with SEM, and the value obtained with *Igh* ^∆890/∆890^ pro‐B cells was set to 1. The average recombination frequency of each distal V_H_ gene in pro‐B cells of the indicated genotypes is shown in Fig EV3C.Comparison of the VDJ‐seq data obtained with pro‐B cells of the *Igh* ^+/+^ (black), *Igh* ^P4/P4^ (light gray), *Igh* ^P4inv/P4inv^ (blue), and *Igh* ^P4StopG/P4StopG^ (green) genotypes. For further description see (D). Data information: Statistical data were analyzed by multiple *t*‐tests (unpaired and two‐tailed with Holm‐Sidak correction; B, E) or one‐way ANOVA (Tukey *post hoc* test; C, F); *P < 0.05, **P < 0.01, ***P < 0.001, ****P < 0.0001. Each dot (B, C, E, and F) corresponds to one mouse. Source data are available online for this figure.

**Figure EV3:**
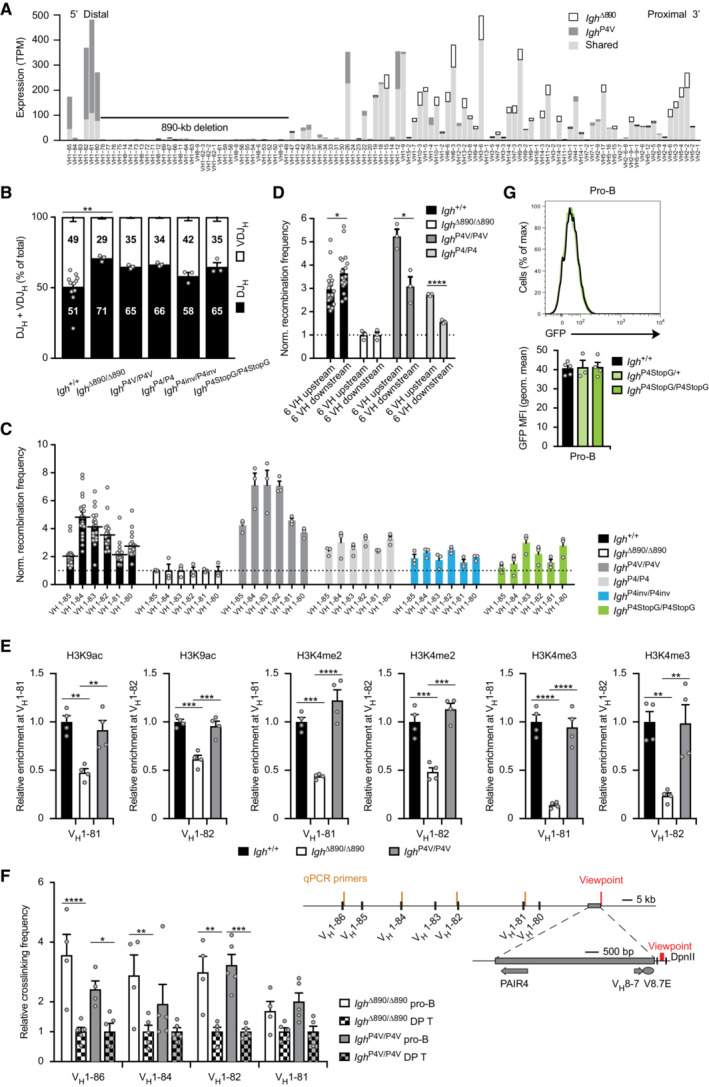
Activation of distal V_H_ gene recombination upon reconstitution of the *Igh* ^∆890^ allele with different PAIR4 constructs V_H_ gene expression from the *Igh* ^P4V(B6)^ and *Igh* ^∆890(B6)^ alleles in immature IgM^b^ (B6) B cells sorted from the bone marrow of *Igh* ^P4V(B6)/+(129)^ and *Igh* ^∆890(B6)/+(129)^ mice, respectively (see also Fig 3B). The expression (TPM) value of each V_H_ gene is shown as shared (light gray) or unique expression of the *Igh* ^P4V(B6)^ allele (dark gray) or *Igh* ^∆890(B6)^ allele (white bars). The expression data are shown as mean TPM values and are based on three or two independent RNA‐seq experiments for the immature B cells of *Igh* ^P4V(B6)/+(129)^ and *Igh* ^∆890(B6)/+(129)^ mice, respectively. The different V_H_ genes (horizontal axis) are aligned according to their position in the *Igh* locus (Dataset EV1). The 890‐kb deletion is indicated by a black line. TPM, transcripts per million; B6, C57BL/6 strain; 129, 129/Sv strain.VDJ‐seq analysis of *Igh* rearrangements in pro‐B cells of the indicated genotypes. The percentages of uniquely identified DJ_H_ and VDJ_H_ sequences are shown as mean percentage with SEM. The high variance caused by the many data points obtained with control *Igh* ^+/+^ pro‐B cells is likely responsible for the fact that only the comparison between *Igh* ^+/+^ and *Igh* ^∆890/∆890^ pro‐B cells reached statistical significance.Normalized recombination frequency of the first six distal V_H_ genes (V_H_1‐85 to V_H_1‐80) determined in pro‐B cells of the indicated genotypes, based on the data shown in Fig 3D and G. The recombination frequency of each of the six distal V_H_ genes was calculated as a mean value with SEM, and the value obtained with *Igh* ^∆890/∆890^ pro‐B cells was set to 1 (dashed line).The PAIR4‐V8.7E module and PAIR4 elements differentially activate the recombination of V_H_ genes located upstream or downstream of their insertion. The normalized recombination frequency of the first six upstream V_H_ genes (V_H_1‐85 to V_H_1‐80) and the first six downstream V_H_ genes (V_H_1‐47, V_H_1‐43, V_H_1‐42, V_H_1‐39, V_H_1‐37, V_H_1‐36; Fig EV2B) was determined in pro‐B cells of the indicated genotypes, based on the data shown in Fig 3D and G. The average recombination frequency of the six upstream and six downstream V_H_ genes was calculated as mean value with SEM, and the value obtained with *Igh* ^∆890/∆890^ pro‐B cells was set to 1.Active chromatin at the V_H_1‐81 and V_H_1‐82 genes in *Igh* ^P4V/P4V^, *Igh* ^∆890/∆890^ and *Igh* ^+/+^ pro‐B cells. Short‐term cultured pro‐B cells of the indicated genotypes were used for native ChIP analysis with anti‐H3K9ac, anti‐H3K4me2 and anti‐H3K4me3 antibodies. Input and precipitated DNA were quantified by qPCR analysis with primers amplifying the sequences of the V_H_1‐81, V_H_1‐82 and *Tbp* genes. The amount of precipitated DNA was calculated as mean percentage of input with SEM and was normalized to the value obtained for the positive *Tbp* control. The relative enrichment is shown by setting the normalized value of the control *Igh* ^+/+^ pro‐B cells to 1.3C‐qPCR analysis of the long‐range interactions between the PAIR4‐V8.7E module and distal V_H_ genes in sorted bone marrow pro‐B cells and CD4^+^CD8^+^ double‐positive (DP) thymocytes from *Igh* ^P4V/P4V^ and *Igh* ^∆890/∆890^ mice. The relative crosslinking frequencies between the reference DpnII fragment (viewpoint, located downstream of the insertion site) and the indicated distal V_H_ genes were determined with independently prepared 3C‐templates and were normalized relative to the crosslinking frequency measured for the control *Ercc3* gene (see Materials and Methods). The mean crosslinking frequency with SEM is shown for each V_H_ gene by setting the mean value obtained with DP T cells for each genotype to 1.Absence of GFP expression in pro‐B cells of *Igh* ^P4StopG/+^ (light green) and *Igh* ^P4StopG/P4StopG^ (green) mice compared with control *Igh* ^+/+^ (black) mice, as indicated by flow‐cytometric analysis and geometric mean fluorescence intensity measurement. Geometric mean fluorescence intensity measurements are shown as a mean value with SEM. Data information: Statistical data (B, D, E, F, G) were analyzed by one‐way ANOVA (Tukey *post hoc* test); *P < 0.05, **P < 0.01, ***P < 0.001, ****P < 0.0001. Each dot (B‐G) corresponds to one mouse. Source data are available online for this figure.

We next investigated whether the insertion of distinct PAIR4 constructs at the deletion point of the *Igh* ^∆890^ allele could rescue the recombination of adjacent distal V_H_ genes. To this end, we generated *Igh* ^∆890/+^ *Rosa26* ^CreERT2/+^ ES cells to be able to use the Floxin system (Singla *et al*, 2010) for Cre‐mediated insertion of different PAIR4 constructs (Fig 3A) at the *lox*71 site, which was left after deletion in the *Igh* ^∆890^ allele (Fig EV2A). We first generated the *Igh* ^P4V^ allele carrying the insertion of PAIR4 and its associated V8.7E element (Fig 3A). As the lncRNA transcribed from the PAIR4 element, which we refer to as PAIR4‐derived transcript, is spread over a 26‐kb sequence (Fig EV1A), we could not insert this large region together with the PAIR4 and V8.7E elements into the *Igh* ^P4V^ allele due to size constraints imposed by the Floxin method. Instead, we linked the cDNA sequence of the spliced PAIR4‐derived transcript (RIKEN clone CJ056205) in exon 2 of PAIR4 (Ebert *et al*, 2011) and added six polyadenylation sites at the 3′ end, followed by Floxin‐mediated insertion to create the *Igh* ^P4V^ allele (Fig 3A). We next compared the *Igh* ^P4V^ and *Igh* ^∆890^ alleles with the wild‐type *Igh* ^+^ allele in the following competitive setting. The mutant *Igh* alleles were of the C57BL/6 (B6) origin and therefore encode the IgM^b^ BCR, while *Igh* alleles of the 129/Sv (129) strain give rise to the expression of the IgM^a^ isotype. By crossing mice of these two strains, we determined the ratio of immature IgM^b^ (B6) to IgM^a^ (129) B cells in the bone marrow of *Igh* ^∆890(B6)/+(129)^ and control *Igh* ^+(B6)/+(129)^ mice using flow‐cytometric analysis. This analysis revealed that the *Igh* ^∆890(B6)^ allele generated, with a ratio of 0.58, only about half as many immature IgM^b^ B cells (CD19^+^B220^+^IgM^+^IgD^−^) compared with the *Igh* ^+(B6)^ allele (ratio of 1) in this competitive setting (Fig 3B and C). Notably, the *Igh* ^P4V(B6)^ allele gave rise to a significant 1.3‐fold increase of IgM^b^ B cells (ratio of 0.73) relative to the *Igh* ^∆890(B6)^ allele, which likely reflects an increase of V_H_ gene recombination. We next performed RNA‐sequencing of immature IgM^b^ B cells, which were sorted from the bone marrow of *Igh* ^P4V(B6)/+(129)^ or control *Igh* ^∆890(B6)/+(129)^ mice. This analysis revealed that the insertion of PAIR4 together with its associated V8.7E element strongly increased the expression of four V_H_ genes (V_H_1‐80, 81, 82, 85) at the 5′ end of the *Igh* locus (Fig EV3A). Together, these data suggested that V_H_ gene recombination may have been increased upon reinsertion of the PAIR4‐V8.7E sequences.

VDJ‐seq analysis demonstrated that the inserted V_H_8‐7 gene in *Igh* ^P4V/P4V^ pro‐B cells underwent V_H_‐DJ_H_ recombination at a similar frequency as the V_H_8‐7 gene present in its normal location in the *Igh* locus of wild‐type pro‐B cells (Fig 3D and E). In addition, VDJ‐seq analysis revealed an increase in VDJ_H_‐rearranged *Igh* alleles at the expense of DJ_H_‐rearranged *Igh* alleles in *Igh* ^P4V/P4V^ pro‐B cells compared with *Igh* ^∆890/∆890^ pro‐B cells (Fig EV3B). Notably, the inserted PAIR4 and V8.7E elements induced the recombination of the distal V_H_ genes adjacent to the insertion site in an asymmetrical manner (Fig 3D). The most prominent rescue of recombination was seen for the first six distal V_H_ genes (V_H_1‐85 to V_H_1‐80) upstream of the inserted PAIR4 and V8.7E elements in *Igh* ^P4V/P4V^ pro‐B cells (Figs 3D and EV3C), consistent with the observed increase in expression of these V_H_ genes in immature *Igh* ^P4V/P4V^ B cells (Fig EV3A). The average recombination frequency of these six V_H_ genes in *Igh* ^P4V/P4V^ pro‐B cells was increased 5.2‐fold compared with *Igh* ^∆890/∆890^ pro‐B cells (Fig 3F). Notably, the average recombination frequency of the six V_H_ genes (V_H_1‐47, 43, 42, 39, 37, 36), which are located downstream of the PAIR4‐V8.7E insertion (Fig EV2B), was reduced 2‐fold compared with that of the upstream V_H_ genes in *Igh* ^P4V/P4V^ pro‐B cells (Fig EV3D), thus indicating a directional activity of the inserted PAIR4 and V8.7E elements in controlling the recombination of V_H_ genes.

We next investigated whether the recombination frequency correlated with the abundance of active chromatin at these distal V_H_ genes. ChIP‐qPCR analysis of *Igh* ^P4V/P4V^, *Igh* ^∆890/∆890^, and *Igh* ^+/+^ pro‐B cells revealed that the level of H3K9ac, H3K4me2, and H3K4me3 at the V_H_1.81 and V_H_1.82 genes was significantly reduced in *Igh* ^∆890/∆890^ pro‐B cells compared with *Igh* ^+/+^ pro‐B cells (Fig EV3E). Importantly, the abundance of all three active histone marks at these two V_H_ genes was rescued by insertion of the PAIR4‐V8.7E module in *Igh* ^P4V/P4V^ pro‐B cells (Fig EV3E). To explore whether the PAIR4‐V8.7E module may induce active chromatin by specifically promoting its long‐distance interaction with the V_H_1.81 and V_H_1.82 genes, we performed 3C‐qPCR analysis (Oudelaar *et al*, 2017) with sorted pro‐B cells and control double‐positive (DP) T cells from *Igh* ^P4V/P4V^ and *Igh* ^∆890/∆890^ mice. To this end, we have chosen a DpnII fragment as a viewpoint, which is located downstream of the insertion site and adjacent to the inserted V8.7E enhancer (Fig EV3F). The 3C‐qPCR analysis revealed that the interactions from this viewpoint to the V_H_1‐81, V_H_1‐82, V_H_1‐84, and V_H_1‐86 genes was increased in pro‐B cells relative to the control T cells. Notably, the increased interactions to these V_H_ genes were observed in both *Igh* ^P4V/P4V^ and *Igh* ^∆890/∆890^ pro‐B cells, indicating that their formation does not depend on the presence of the PAIR4‐V8.7E module (Fig EV3F). In summary, these data demonstrate that the insertion of PAIR4 and its associated V8.7E element strongly activated recombination of the six most distal V_H_ genes over a distance of up to 112 kb by inducing active chromatin without promoting the long‐range interactions with these V_H_ genes.

### The PAIR4 and V8.7E elements function as enhancers of V_H_ gene recombination

To analyze the contribution of the V8.7E element and PAIR4 orientation to the rescue of distal V_H_ gene recombination, we generated the *Igh* ^P4^ and *Igh* ^P4inv^ alleles, containing the PAIR4 element in the normal forward or inverted (inv) orientation in the absence of the V8.7E element (Fig 3A). The analysis of immature B cells from *Igh* ^P4(B6)/+(129)^ and *Igh* ^P4inv(B6)/+(129)^ mice in the competition assay described above revealed that the *Igh* ^P4(B6)^ allele generated only a minimally reduced frequency (ratio of 0.69) of IgM^b^ B cells compared with the *Igh* ^P4V(B6)^ allele (ratio of 0.73). The frequency (ratio of 0.63) of the IgM^b^ B cells generated by the *Igh* ^P4inv(B6)^ allele was more strongly reduced, but still above the frequency (ratio of 0.58) of the *Igh* ^∆890(B6)^ allele (Fig 3C), indicating that both PAIR4 insertions were still able to promote V_H_ gene recombination. VDJ‐seq analysis demonstrated that the insertion of the PAIR4 element in its normal or inverted orientation in the *Igh* locus of *Igh* ^P4/P4^ and *Igh* ^P4inv/P4inv^ pro‐B cells increased distal V_H_ gene recombination by a factor of 2.7 or 1.9, respectively, relative to the level seen in *Igh* ^∆890/∆890^ pro‐B cells (Fig 3F and G). These data therefore indicated that the PAIR4 element in its normal forward orientation promoted distal V_H_ gene recombination better than in its inverted orientation. Importantly, as the PAIR4 element was maximally active with a 5.2‐fold increase of distal V_H_ gene recombination in the presence of its associated V8.7E element in *Igh* ^P4V/P4V^ pro‐B cells (Fig 3D and F), we conclude that the PAIR4 and V8.7E elements function as enhancers of V_H_ gene recombination, which together constitute one regulatory module.

We next investigated whether the transcription of the spliced PAIR4‐derived transcript contributes to the activity of the PAIR4 element. For this, we inserted six copies of a synthetic polyadenylation sequence (Levitt *et al*, 1989) coupled to a *Gfp* reporter gene into the first exon of the PAIR4‐derived transcript to generate the *Igh* ^P4StopG^ allele (Fig 3A). Pro‐B cells of *Igh* ^P4StopG/P4StopG^ mice failed to express GFP, thus indicating that the six polyadenylation sites interfered with transcription of the downstream *Gfp* gene (Fig EV3G), which was further confirmed by RT‐qPCR analysis (Fig EV5C). Despite the inability to express a spliced PAIR4‐derived transcript, the *Igh* ^P4StopG/P4StopG^ pro‐B cells still gave rise to a 1.9‐fold increase in distal V_H_ gene recombination, which was, however, lower than the 2.7‐fold effect seen with *Igh* ^P4/P4^ pro‐B cells (Fig 3F and G), suggesting that the spliced PAIR4‐derived transcript may weakly contribute to the function of PAIR4.

### Long‐range activation of distal V_H_ gene recombination by the V8.8E enhancer

**Figure 4:**
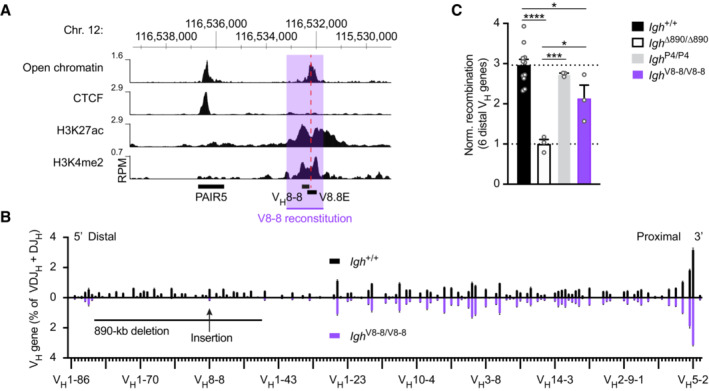
Long‐range activation of distal V_H_ gene recombination by the V8.8E enhancer in *Igh* ^V8‐8/V8‐8^ mice Presence of open chromatin and the active histone marks H3K27ac and H3K4me2 at the V8.8E enhancer in pro‐B cells. The location of the V_H_8‐8 gene, V8.8E enhancer (dashed line), and PAIR5 element are shown together with the mm9 genomic coordinates of mouse chromosome 12. The V_H_8‐8 gene with 500 bp of its upstream and downstream sequences (indicated by violet overlay) was inserted with the Floxin method at the deletion point of the *Igh* ^∆890^ allele to generate the *Igh* ^V8‐8^ allele (Hill *et al*, 2020).Comparison of the VDJ‐seq data obtained with pro‐B cells from the bone marrow of *Igh* ^+/+^ (black) and *Igh* ^V8‐8/V8‐8^ (violet) mice. The relative frequency of each V_H_ gene is shown as a mean value with SEM and is based on five (*Igh* ^+/+^) or three (*Igh* ^V8‐8/V8‐8^) independent VDJ‐seq experiments. The different V_H_ genes (horizontal axis) are aligned according to their position in the *Igh* locus (Dataset EV1). For further explanation, see legend of Fig 3D.Normalized recombination frequency of the first six distal V_H_ genes (V_H_1‐85 to V_H_1‐80) determined in pro‐B cells from *Igh* ^V8‐8/V8‐8^ (violet), *Igh* ^+/+^ (black), *Igh* ^∆890/∆890^ (white bar) and *Igh* ^P4/P4^ (gray) mice. The average recombination frequency of the six distal V_H_ genes was calculated as a mean value with SEM, and the value obtained with *Igh* ^∆890/∆890^ pro‐B cells was set to 1. Statistical data were analyzed by one‐way ANOVA (Tukey *post hoc* test); *P < 0.05, ***P < 0.001, ****P < 0.0001. Each dot corresponds to a mouse. Source data are available online for this figure.

To investigate whether a V_H_8‐associated element on its own is able to promote distal V_H_ gene recombination, we analyzed the function of the V8.8E element located downstream of the V_H_8‐8 gene. This element was characterized by the presence of open chromatin and the active histone marks H3K4me2 and H3K27ac, suggesting that it may function as an active regulatory element in pro‐B cells (Figs 1E and 4A). We previously generated the *Igh* ^V8‐8^ allele by inserting the V_H_8‐8 gene with 500 bp of its upstream and downstream sequences at the deletion point of the *Igh* ^∆890^ allele (Hill *et al*, 2020). Importantly, the inserted downstream sequences of the V_H_8‐8 gene encompassed the V8.8E element (Fig 4A). As shown by VDJ‐seq analysis, the V8.8E element was able to activate the recombination of the first six distal V_H_ genes 2.1‐fold in *Igh* ^V8‐8/V8‐8^ pro‐B cells compared with *Igh* ^∆890/∆890^ pro‐B cells (Fig 4B and C) and was thus only slightly less active than the PAIR4 element on its own in *Igh* ^P4/P4^ pro‐B cells (2.7‐fold). We conclude therefore that V8.8E element can function as an enhancer to promote long‐range activation of distal V_H_ gene recombination.

### The regulatory PAIR4‐V8.7E module is highly active only in pro‐B cells within the hematopoietic system

**Figure 5:**
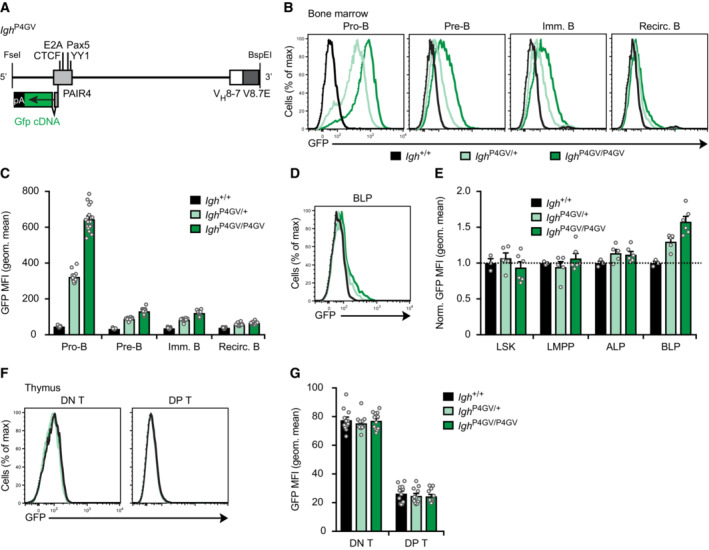
Pro‐B cell‐specific activity of the PAIR4‐V8.7E enhancer module A
Schematic diagram of the *Gfp*‐linked PAIR4‐V8.7E module. The *Gfp* cDNA linked to 6 polyadenylation sites (pA) was inserted in exon 2 of PAIR4 prior to the insertion of the entire module at the deletion point into the *Igh* ^∆890^ allele by the Floxin method.B, C
Flow‐cytometric analysis (B) and geometric mean fluorescence intensity measurements (MFI, C) of GFP expression are shown for pro‐B, pre‐B, immature B, and recirculating B cells from the bone marrow of *Igh* ^P4GV/P4GV^ (dark green, *n* = 16), *Igh* ^P4GV/+^ (light green, *n* = 12), and control *Igh* ^+/+^ (black, *n* = 13) mice. Geometric mean fluorescence intensity measurements (C) are shown as mean values with SEM.D, E
Induction of GFP expression in a small subset of BLPs, as shown by flow‐cytometric analysis (D) and geometric mean fluorescence intensity measurements (E) of GFP expression in MPPs (LSK), LMPPs, ALPs, and BLPs from the bone marrow of the indicated mice. The dashed line indicates the absence of GFP expression in *Igh* ^+/+^ cells, which were set to 1 for normalization of the MFI data. Geometric mean fluorescence intensity measurements (E) are shown as mean value with SEM based on 3 (*Igh* ^+/+^), 5 (*Igh* ^P4GV/+^) or 6 (*Igh* ^P4GV/P4GV^) independent replicates.F, G
Absence of GFP expression in DN (CD4^−^CD8^−^) and DP (CD4^+^CD8^+^) T cells, as shown by flow‐cytometric analysis (F) and geometric mean fluorescence intensity measurements (G) of GFP expression in thymocytes from the indicated mice. Geometric mean fluorescence intensity measurements (G) are shown as a mean value with SEM based on 12 (*Igh* ^+/+^), 11 (*Igh* ^P4GV/+^), or 12 (*Igh* ^P4GV/P4GV^) independent replicates. Data information: The flow‐cytometric definition of the lymphoid progenitors, B and T cell types is described in Materials and Methods. Source data are available online for this figure.

We next studied the developmental activity of PAIR4 together with its associated V8.7E enhancer within the hematopoietic system. For this, we linked the PAIR4 element to a *Gfp* gene in exon 2 of the PAIR4‐derived transcript so that we could monitor GFP expression as a proxy for PAIR4 activity. We then used the Floxin method to insert the PAIR4‐Gfp element together with the V8.7E enhancer into the *Igh* ^∆890^ allele to generate the *Igh* ^P4GV^ allele (Fig 5A). Within the B cell lineage, GFP expression was highest in pro‐B cells of *Igh* ^P4GV/+^ and *Igh* ^P4GV/P4GV^ mice (Fig 5B and C). GFP expression was already strongly reduced in pre‐B and immature B cells and was largely lost in recirculating B cells in the bone marrow of *Igh* ^P4GV/+^ and *Igh* ^P4GV/P4GV^ mice (Fig 5B and C). Importantly, no GFP expression was detected in multipotent progenitors (LSK), lymphoid‐primed multipotent progenitors (LMPP) and all‐lymphoid progenitors (ALP), while GFP expression was initiated only in a small fraction of BLPs just prior to the generation of committed pro‐B cells (Fig 5D and E). Notably, no GFP expression was detected in CD4^−^CD8^−^ double‐negative (DN) and CD4^+^CD8^+^ double‐positive (DP) T cells in the thymus of *Igh* ^P4GV/+^ and *Igh* ^P4GV/P4GV^ mice (Fig 5F and G). In summary, the activity of PAIR4 is induced to a high level only in committed pro‐B cells, rapidly declines in pre‐B and immature B cells and is absent in lymphoid progenitors and T‐lineage cells.

### Opposing roles of Pax5 and CTCF in regulating PAIR4 activity

**Figure 6:**
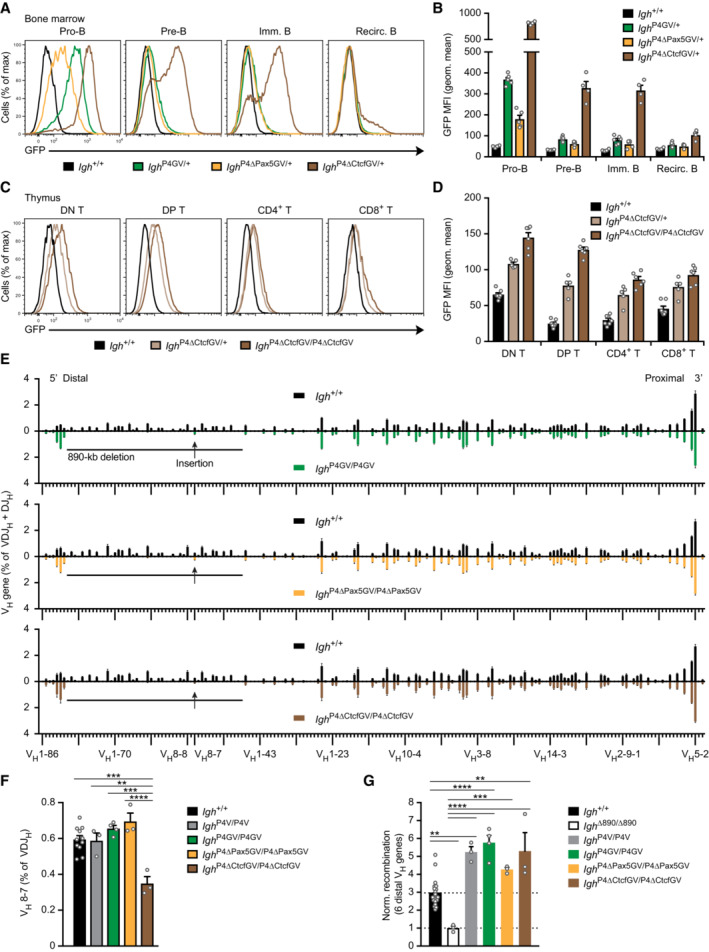
The CTCF binding site of PAIR4 restricts the activity of the PAIR4‐V8.7E module to the pro‐B cell stage A, B
Flow‐cytometric analysis (A) and geometric mean fluorescence intensity measurements (MFI, B) of GFP expression are shown for pro‐B, pre‐B, immature B, and recirculating B cells from the bone marrow of *Igh* ^P4GV/+^ (green, *n* = 6), *Igh* ^P4∆Pax5GV/+^ (yellow, *n* = 5), *Igh* ^P4∆CtcfGV/+^ (brown, *n* = 4), and control *Igh* ^+/+^ (black, *n* = 4) mice. Geometric mean fluorescence intensity measurements (B) are shown as a mean value with SEM.C, D
Activation of the PAIR4‐V8.7E module in thymocytes upon loss of CTCF binding at PAIR4, as shown by flow‐cytometric analysis (C) and geometric mean fluorescence intensity measurements (MFI, D) of GFP expression in DN, DP, CD4^+^, and CD8^+^ thymocytes of *Igh* ^P4∆CtcfGV/+^ (light brown, *n* = 5) and *Igh* ^P4∆CtcfGV/P4∆CtcfGV^ (dark brown, *n* = 6) mice compared with control *Igh* ^+/+^ (black, *n* = 7) mice. Geometric mean fluorescence intensity measurements (D) are shown as a mean value with SEM.E
Comparison of the VDJ‐seq data obtained with pro‐B cells of the *Igh* ^+/+^ (black), *Igh* ^P4GV/P4GV^ (green), *Igh* ^P4∆Pax5GV/P4∆Pax5GV^ (yellow), and *Igh* ^P4∆CtcfGV/P4∆CtcfGV^ (brown) genotypes. The relative frequency of each V_H_ gene is shown as mean value with SEM and is based on four (*Igh* ^+/+^, *Igh* ^P4GV/P4GV^) or three (*Igh* ^P4∆Pax5GV/P4∆Pax5GV^, *Igh* ^P4∆CtcfGV/P4∆CtcfGV^) independent VDJ‐seq experiments. The different V_H_ genes (horizontal axis) are aligned according to their position in the *Igh* locus (Dataset EV1). For further explanation, see legend of Fig 3D.F
Recombination frequency of the inserted V_H_8‐7 gene, which was determined as percentage of all VDJ_H_ recombination events in pro‐B cells of the indicated genotypes and is shown as a mean value with SEM.G
Normalized recombination frequency of the first six distal V_H_ genes (V_H_1‐85 to V_H_1‐80) determined in pro‐B cells of the indicated genotypes, based on the data shown in (E). The average recombination frequency of the six distal V_H_ genes was calculated as mean value with SEM, and the value obtained with *Igh* ^∆890/∆890^ pro‐B cells was set to 1. Data information: Statistical data were analyzed by one‐way ANOVA (Tukey *post hoc* test; F, G); **P < 0.01, ***P < 0.001, ****P < 0.0001. Each dot (B, D, F, and G) corresponds to a mouse. Source data are available online for this figure.

**Figure EV4:**
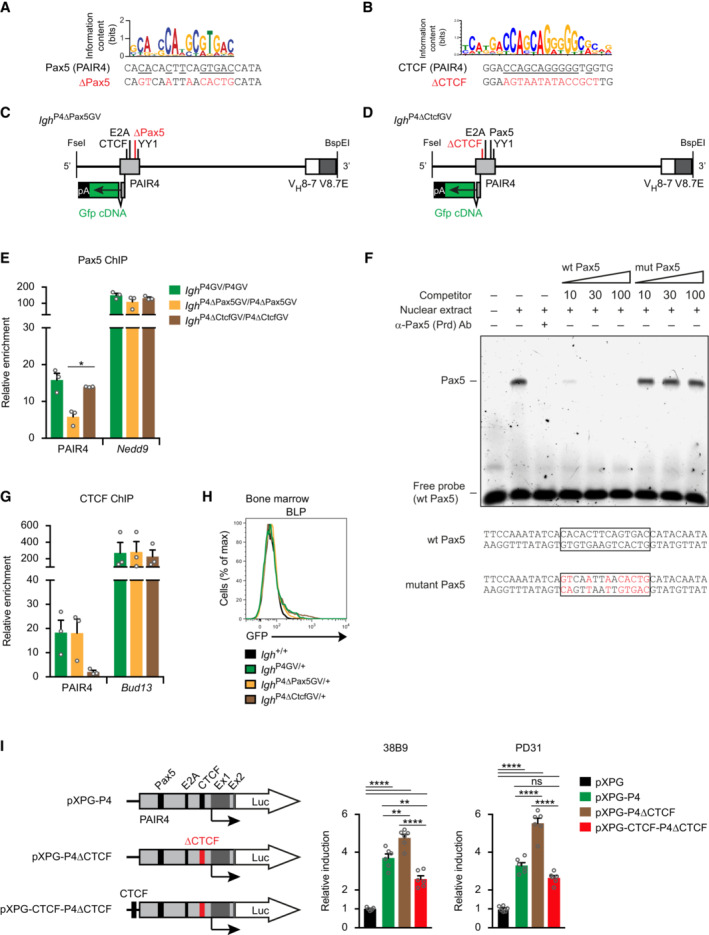
Mutation of the Pax5‐ and CTCF‐binding sites in the PAIR4 element A
Mutation of the Pax5‐binding sequence of PAIR4. The nucleotides of PAIR4, which match the Pax5 consensus recognition sequence (Kaiser *et al*, 2022), are underlined and were mutated to the nucleotides indicated in red (∆Pax5).B
Mutation of the CTCF‐binding sequence of PAIR4. The nucleotides of PAIR4, corresponding to the consensus CTCF‐binding motif (Hill *et al*, 2020), are underlined and were mutated to the nucleotides indicated in red (∆CTCF).C, D
Schematic diagram of the *Gfp*‐linked PAIR4‐V8.7E module with the Pax5‐ or CTCF‐binding site mutation, which was used for the generation of the *Igh* ^P4∆Pax5GV^ or *Igh* ^P4∆CtcfGV^ allele, respectively.E, G
Pax5 and CTCF binding to the wild‐type and mutant PAIR4 sequences. Short‐term cultured pro‐B cells from the bone marrow of *Igh* ^P4GV/P4GV^ (green), *Igh* ^P4∆Pax5GV/P4∆Pax5GV^ (yellow), and *Igh* ^P4∆CtcfGV/P4∆CtcfGV^ (brown) mice were used for ChIP analysis with an anti‐Pax5 paired domain antibody or an anti‐CTCF antibody. Input and precipitated DNA were quantified by qPCR analysis with primers amplifying the Pax5‐binding site present in PAIR4 or the *Nedd9* gene (E), the CTCF‐binding site present in PAIR4 or the *Bud13* gene (G) or by amplifying a gene‐poor region on chromosome 1 as a negative control (Dataset EV2). The amount of precipitated DNA was calculated as an average percentage of input with SEM and is shown relative to the value obtained for the negative control.F
No binding of Pax5 to the mutant Pax5 recognition sequence (∆Pax5) of PAIR4. A fluorescently‐labeled double‐stranded oligonucleotide containing the wild‐type (wt) Pax5 recognition sequence of PAIR4 (shown below) was used as a probe for electrophoretic mobility shift assay (EMSA) with a nuclear extract prepared from B cells of the human Ramos cell line (see Materials and Methods). The Pax5‐DNA complex (marked as Pax5 to the left) was not formed upon addition of an anti‐Pax5 antibody, directed against the DNA‐binding paired domain (Prd), or in the presence of a 10‐, 30‐, and 100‐fold molar excess of a nonlabeled competitor oligonucleotide containing the wild‐type Pax5‐binding site in contrast to the competitor oligonucleotide containing the mutant (mut) Pax5 recognition sequence (shown below).H
Absence of GFP expression in BLPs from the bone marrow of *Igh* ^P4GV/+^ (green), *Igh* ^P4∆Pax5GV/+^ (yellow), *Igh* ^P4∆CtcfGV/+^ (brown), and control *Igh* ^+/+^ (black) mice, as shown by flow‐cytometric analysis.I
Transient transfection reporter assay. The PAIR4‐luciferase reporter genes (schematically shown to the left) were transfected together with the control vector pRL‐CMV into cells of the pro‐B cell line 38B9 (Alt *et al*, 1984) or pre‐B cells line PD31 (Lewis *et al*, 1982). After 24 h, luciferase activities were measured, normalized and displayed relative to the activity of the parental vector pXPG, containing a promoter‐less firefly luciferase gene, which was set to 1. The relative luciferase (luc) activities of six independent transfection experiments are shown as mean values with SEM. Ex1 and Ex2 refer to exons 1 and 2 of the PAIR antisense transcript. Data information: Statistical data (E, G, I) were analyzed by one‐way ANOVA (Tukey *post hoc* test); ns *P* > 0.05, *P < 0.05; **P < 0.01, ****P < 0.0001. Source data are available online for this figure.

To investigate the role of Pax5 and CTCF in controlling the activity of PAIR4, we next mutated the binding site of each transcription factor in the PAIR4 sequence. For this, we mutated nine nucleotide positions of the Pax5‐binding site (Fig EV4A) and exchanged all nucleotides of the CTCF‐binding site (Fig EV4B) in PAIR4 to generate the *Igh* ^P4∆Pax5GV^ and *Igh* ^P4∆CtcfGV^ alleles, respectively (Fig EV4C and D). We next performed ChIP‐qPCR analysis with short‐term cultured pro‐B cells to investigate the effect of the introduced mutations on Pax5 and CTCF binding. The interaction of Pax5 with PAIR4 was 3‐fold reduced in *Igh* ^P4∆Pax5GV/P4∆Pax5GV^ pro‐B cells relative to control *Igh* ^P4GV/P4GV^ pro‐B cells (Fig EV4E). As the extensive mutation of the Pax5 recognition sequence in the PAIR4 element (Fig EV4A) is expected to abolish Pax5 binding, we further analyzed the interaction of Pax5 with its wild‐type or mutant binding site by electrophoretic mobility shift assay with a B cell nuclear extract (Fig EV4F). While the formation of the Pax5‐DNA complex was abolished in the presence of an anti‐Pax5 paired domain antibody or an excess of nonlabeled oligonucleotide containing the wild‐type Pax5‐binding site, a similar excess of the mutant oligonucleotide was unable to compete with Pax5 binding to the wild‐type probe (Fig EV4F). As the introduced mutations abolished Pax5 binding to PAIR4, it is therefore likely that the residual Pax5 binding observed by ChIP analysis in *Igh* ^P4∆Pax5GV/P4∆Pax5GV^ pro‐B cells may be caused by Pax5, bound to its site in the V8.7E enhancer (Fig EV1A), through a looping interaction with PAIR4 within the same regulatory module. GFP expression was 2‐fold reduced in pro‐B cells of *Igh* ^P4∆Pax5GV/+^ mice compared with *Igh* ^P4GV/+^ mice (Fig 6A and B). Hence, these results indicate that Pax5 binding is required for maximal activity of PAIR4 in pro‐B cells.

Mutation of the CTCF recognition site completely abrogated binding of CTCF to PAIR4, as shown by ChIP‐qPCR analysis of *Igh* ^P4∆CtcfGV/P4∆CtcfGV^ pro‐B cells (Fig EV4G). GFP expression was 2‐fold increased in pro‐B cells of *Igh* ^P4∆CtcfGV/+^ mice compared with *Igh* ^P4GV/+^ mice (Fig 6A and B), consistent with the previous observation that the downregulation of CTCF protein expression in cultured pro‐B cells leads to enhanced transcription of the PAIR4‐derived lncRNA (Degner *et al*, 2011). Notably, there was a similarly small fraction of GFP^int^ BLPs in the bone marrow of *Igh* ^P4∆CtcfGV/+^ and *Igh* ^P4GV/+^ mice (Fig EV4H), demonstrating that the loss of CTCF binding led to a 2‐fold increase of GFP expression only upon transition to the pro‐B cell stage. Unexpectedly however, GFP expression remained at the same high level in pre‐B and immature B cells of *Igh* ^P4∆CtcfGV/+^ mice as in *Igh* ^P4GV/+^ pro‐B cells, while GFP expression was largely lost in recirculating *Igh* ^P4∆CtcfGV/+^ B cells (Fig 6A and B). The biphasic distribution of the GFP protein levels in *Igh* ^P4∆CtcfGV/+^ pre‐B and immature B cells likely indicates that the majority of GFP^hi^ cells still actively transcribed the *Gfp* gene, while the smaller faction of GFP^lo^ cells in the absence of *Gfp* mRNA synthesis progressively lost the GFP protein with its relatively long half‐life of 2–3 days (Nagaoka *et al*, 2000). Most surprisingly, GFP expression was also observed in CD4^−^CD8^−^ (DN), CD4^+^CD8^+^ (DP), CD4^+^ and CD8^+^ T cells in the thymus of *Igh* ^P4∆CtcfGV/+^ and *Igh* ^P4∆CtcfGV/P4∆CtcfGV^ mice (Fig 6C and D). These data therefore demonstrate that the CTCF‐binding site in PAIR4 restrains the activity of this element in B and T cells. We next performed transient transfection assays with wild‐type and mutant PAIR4‐luciferase constructs in established pro‐B (38B9) and pre‐B (PD31) cell lines (Fig EV4I) to investigate whether CTCF can function as a repressor to restrain the transcriptional activity of PAIR4. While the PAIR4‐luciferase gene (pXPG‐P4) gave rise to a 3.5‐fold increase of luciferase activity relative to the promoter‐less luciferase gene (pXPG) in both cell lines, its activity was further increased upon mutation of the CTCF‐binding site (pXPG‐P4∆CTCF; Fig EV4I). Notably, the activity of the mutant PAIR4‐luciferase gene was 2‐fold reduced upon addition of the wild‐type CTCF‐binding sequence at the 5′ end of PAIR4 (pXPG‐CTCF‐P4∆CTCF; Fig EV4I). These data therefore indicate that CTCF can function as a repressor of PAIR4 activity (Fig EV4I). In summary, we conclude that Pax5 is required for maximal activity of PAIR4 in pro‐B cells, whereas CTCF contributes to the pro‐B cell‐specific activity of PAIR4 by preferentially suppressing the activity of this element at later B cell developmental stages and during T lymphopoiesis.

### Binding of Pax5 and CTCF at PAIR4 differentially affects V_H_ gene recombination

We next studied the role of Pax5 and CTCF for PAIR4‐mediated V_H_ gene recombination by performing VDJ‐seq analysis with sorted pro‐B cells from *Igh* ^P4GV/P4GV^, *Igh* ^P4∆Pax5GV/P4∆Pax5GV^ and *Igh* ^P4∆CtcfGV/P4∆CtcfGV^ mice (Fig 6E). Notably, the recombination efficiency of the PAIR4‐associated V_H_8‐7 gene was neither affected by replacement of the PAIR4‐derived transcript with the *Gfp* gene in *Igh* ^P4GV/P4GV^ pro‐B cells nor by mutation of the Pax5‐binding site in *Igh* ^P4∆Pax5GV/P4∆Pax5GV^ pro‐B cells compared with *Igh* ^P4V/P4V^ pro‐B cells (Fig 6F). Importantly however, loss of the CTCF‐binding site in PAIR4 resulted in a 1.7‐fold reduced recombination frequency of the V_H_8‐7 gene. Hence, the CTCF‐binding site in PAIR4 is essential for efficient recombination of the V_H_8‐7 gene by acting over a short 4.1‐kb distance.

Next, we analyzed the recombination frequencies of the 6 most distal V_H_ genes (Fig 6E and G), which are present at a distance of up to 112 kb away from the inserted PAIR4 element (Fig EV2B). The recombination of these distal V_H_ genes was induced to a similarly high level (5.3‐ and 5.8‐fold) in *Igh* ^P4∆CtcfGV/P4∆CtcfGV^ and *Igh* ^P4GV/P4GV^ pro‐B cells as in *Igh* ^P4V/P4V^ pro‐B cells, when measured relative to the *Igh* ^∆890/∆890^ pro‐B cells containing only the 890‐kb *Igh* deletion (Fig 6G). Although the induction of distal V_H_ gene recombination was still 4.3‐fold in *Igh* ^P4∆Pax5GV/P4∆Pax5GV^ pro‐B cells, it was lower in comparison with the *Igh* ^P4GV/P4GV^ and *Igh* ^P4∆CtcfGV/P4∆CtcfGV^ pro‐B cells (Fig 6G). Together, these data demonstrate that the CTCF‐binding site of PAIR4 promotes V_H_8‐7 gene recombination over a short distance (4.1‐kb), while it is dispensable for distal V_H_ gene recombination over a long distance. In contrast, the Pax5‐binding site is not essential for recombination of the adjacent V_H_8‐7 gene but is required for efficient recombination of distal V_H_ genes located at a longer distance from the PAIR4 element.

### Insertion of the Eμ enhancer in the *Igh* 5′ region promotes distal V_H_ gene recombination

**Figure 7:**
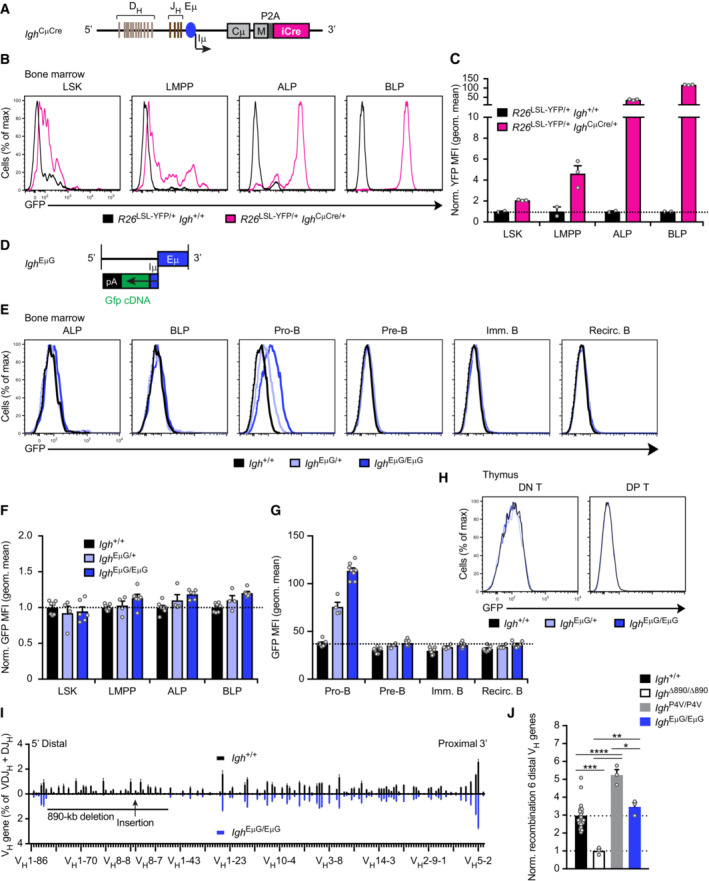
The Eμ enhancer upon insertion in the *Igh* 5′ region promotes distal V_H_ gene recombination and exhibits pro‐B cell‐specific activity A
Schematic diagram of the *Igh* ^CμCre^ allele, which contains the insertion of a *Cre* gene linked via a P2A sequence to the last codon of the second Cμ transmembrane exon (M2) in the *Igh* locus. The generation of the *Igh* ^CμCre^ allele is described in detail in Fig EV5A and Materials and Methods.B, C
Flow‐cytometric analysis (B) and geometric mean fluorescence intensity measurements (MFI, C) of YFP expression are shown for MPPs (LSK), LMPPs, ALPs, and BLPs from the bone marrow of *Rosa26* ^LSL‐YPF/+^ *Igh* ^CμCre/+^ (violet) and control *Rosa26* ^LSL‐YPF/+^ *Igh* ^+/+^ (black) mice. Geometric mean fluorescence intensity measurements (B) are shown as a mean value with SEM and have been normalized by setting the mean value obtained with *Igh* ^+/+^ cells to 1.D
Schematic diagram of the *Gfp*‐linked Iμ‐Eμ sequences used to generate the *Igh* ^EμG^ allele. The *Gfp* cDNA together with six polyadenylation sites (pA) was linked to the Iμ exon downstream of the Eμ enhancer prior to insertion of these sequence at the deletion point into the *Igh* ^∆890^ allele by the Floxin method.E–G
Flow‐cytometric analysis (E) and geometric mean fluorescence intensity measurements (MFI, F, and G) of GFP expression are shown for MPPs (LSK), LMPPs, ALPs, BLPs, pro‐B, pre‐B, immature B, and recirculating B cells from the bone marrow of *Igh* ^EμG/+^ (light blue), *Igh* ^EμG/EμG^ (dark blue), and control *Igh* ^+/+^ (black) mice. Geometric mean fluorescence intensity measurements (B) are shown as mean a value with SEM. For lymphoid progenitors, MFI values have been normalized by setting the mean value obtained with *Igh* ^+/+^ cells to 1. The flow‐cytometric definition of the lymphoid progenitors, B and T cell types is described in Materials and Methods.H
Absence of GFP expression in DN and DP T cells from the thymus of *Igh* ^EμG/+^ (light blue) and *Igh* ^EμG/EμG^ (dark blue) mice compared with control *Igh* ^+/+^ (black) mice.I
Comparison of the VDJ‐seq data obtained with pro‐B cells of the *Igh* ^+/+^ (black) and *Igh* ^EμG/EμG^ (dark blue) genotypes. The relative frequency of each V_H_ gene is shown as mean value with SEM and is based on six (*Igh* ^+/+^) or three (*Igh* ^EμG/EμG^) independent VDJ‐seq experiments. For further description, see legend of Fig 3D.J
Normalized recombination frequency of the first six distal V_H_ genes (V_H_1‐85 to V_H_1‐80) determined in pro‐B cells of the indicated genotypes, based on the data shown in (I). The average recombination frequency of the six distal V_H_ genes was calculated as a mean value with SEM, and the value obtained with *Igh* ^∆890/∆890^ pro‐B cells was set to 1. Data information: Statistical data were analyzed by one‐way ANOVA (Tukey *post hoc* test; J); *P < 0.05, **P < 0.01, ***P < 0.001, ****P < 0.0001. Each dot (C, F, G, and J) corresponds to one mouse. Source data are available online for this figure.

**Figure EV5:**
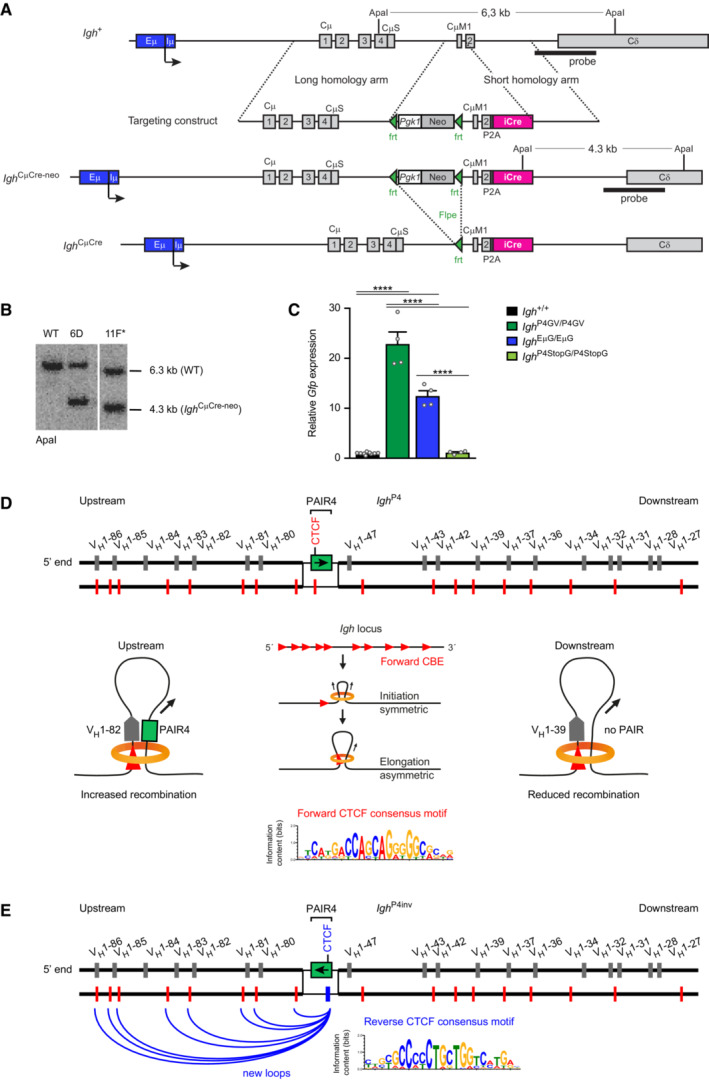
Generation and characterization of the *Igh* ^CμCre^ allele and explanation of recombination differences in the context of loop extrusion Generation of the *Igh* ^CμCre^ allele by ES cell targeting. The *Igh* ^CμCre‐neo^ allele was generated by homologous recombination in the ES cell line A9 by using the following targeting vector. The targeting vector consisted of a 4.1‐kb long 5′ homology region (containing Cμ1 to CμS), a *frt*‐flanked 2.1‐kb DNA fragment containing the mouse phosphoglycerate kinase (*Pgk1*) promoter linked to the neomycin (Neo) resistance gene and a SV40 polyadenylation signal, a 1.6‐kb DNA fragment containing the CμM1/2 sequence linked in frame via the P2A peptide to an iCre gene followed by a 1.8‐kb long 3′ homology region. The *frt* sites are indicated by green arrowheads. The ApaI fragments of the *Igh* ^+^ and *Igh* ^CμCre‐neo^ alleles, which were used for allele identification by Southern blot analysis with the indicated probe, are shown together with their length (in kilobases, kb). The *Igh* ^CμCre^ allele was generated by deletion of the *frt*‐flanked *Pgk1*‐*Neo* expression cassette, which was used for selection of the targeted ES cell clones, in *Igh* ^CμCre‐neo^ mice expressing the Flpe transgene.Southern blot analysis of wild‐type (WT) ES cells and correctly targeted ES cell clones by hybridization of ApaI‐digested genomic DNA with the probe indicated in (A). The ES cell clone 11F was injected into blastocysts to generate the *Igh* ^CμCre‐neo/+^ mouse strain.RT‐qPCR analysis of *Gfp* mRNA expression in sorted pro‐B cells from the bone marrow of the indicated mouse strains. The transcripts of the *Gfp* gene were normalized against the control *Tbp* mRNA, and the mean value obtained with *Igh* ^+/+^ pro‐B cells was set to 1. *Gfp* mRNA expression is shown as a mean value with SEM based on nine (*Igh* ^+/+^) or four (*Igh* ^P4GV/P4GV^, *Igh* ^EμG/EμG^, *Igh* ^P4StopG/P4StopG^) independent samples. Statistical data were analyzed by one‐way ANOVA (Tukey *post hoc* test); ****P < 0.0001.Role of loop extrusion in mediating increased recombination of V_H_ genes located upstream of the insertion of the PAIR4‐V8.7E module (Figs 3G and EV3D). The loop extrusion process has been shown to generate a largely contiguous interaction zone where all the different sequences within the V_H_ gene region appear to interact with each other (Hill *et al*, 2023). The generation of this interaction zone requires that all CTCF‐binding sites are present in forward orientation in the V_H_ gene cluster (Hill *et al*, 2020). Loop extrusion likely initiates at random positions in the V_H_ gene cluster and initially proceeds in a symmetrical manner until the cohesin ring interacts with a CTCF protein bound to the next upstream forward‐oriented CTCF‐binding site, which leads to stabilized binding of cohesin at this site (Li *et al*, 2020). Thereafter, asymmetrical loop extrusion reels the DNA of the downstream *Igh* regions into the loop, until it is halted by a CTCF protein bound to a reverse‐oriented CTCF‐binding site at the IGCR1 or 3′CBE elements (Hill *et al*, 2023). This loop extrusion mechanism predicts that only the V_H_1 genes located upstream of the PAIR4 insertion can efficiently interact through loop extrusion with the inserted PAIR4 element and thus undergo increased V_H_1 gene recombination. In contrast, the downstream V_H_1 genes are unable to interact through this loop extrusion mechanism with the inserted PAIR4 element, which results in reduced V_H_1 gene recombination.Explanation for the reduced recombination efficiency of upstream V_H_1 genes in *Igh* ^P4inv/P4inv^ pro‐B cells compared with *Igh* ^P4/P4^ pro‐B cells (Fig 3G and F). The inversion of PAIR4 also inverts the orientation of the CTCF‐binding site of PAIR4 in *Igh* ^P4inv/P4inv^ pro‐B cells relative to *Igh* ^P4/P4^ pro‐B cells. The reverse‐oriented CTCF‐binding site of PAIR4 is now in convergent orientation relative to the forward CTCF‐binding sites in the upstream region and can thus form new stabilized loops (Rao *et al*, 2014) that interfere with prolonged loop extrusion beyond the inserted PAIR4 element and can therefore prevent interactions of the upstream V_H_ genes with the RAG^+^ recombination center in the *Igh* 3′ region, as previously shown for the inversion of the 890‐kb V_H_ gene region (Hill *et al*, 2020). The forward and reverse CTCF consensus motifs (D, E) are shown. Source data are available online for this figure.

We next asked whether the insertion of any enhancer in the 5′ region of the *Igh* locus could also promote distal V_H_ gene recombination similar to the regulatory PAIR4‐V8.7E module. For this, we have chosen the Eμ enhancer, which is normally located at the 3′ end of the *Igh* locus. One important function of the Eμ enhancer in early lymphopoiesis is to render the surrounding DNA sequences of the *Igh* locus accessible by inducing active chromatin, which promotes D_H_‐J_H_ recombination and expression of the Iμ germline transcript in uncommitted lymphoid progenitors (Perlot *et al*, 2005; Chakraborty *et al*, 2009). To determine the exact onset of Eμ activity in these early lymphoid progenitors, we used homologous recombination in ES cells to insert a *Cre* gene linked via a P2A sequence to the last codon of the second Cμ transmembrane exon (M2) to generate the *Igh* ^CμCre^ allele (Figs 7A and EV5A and B). The Eμ‐mediated expression of the Iμ germline transcript from *Igh* ^CμCre^ allele should thus give rise to two proteins, a Cμ polypeptide and the Cre protein. To monitor Cre activity as a proxy of the Eμ enhancer activity, we crossed the *Igh* ^CμCre^ allele with the *Rosa26* ^LSL‐YPF^ Cre reporter allele (Srinivas *et al*, 2001) to generate *Rosa26* ^LSL‐YPF/+^ *Igh* ^CμCre/+^ mice, which express YFP only after Cre‐mediated excision of the *lox*P‐stop‐*lox*P (LSL) cassette. YFP expression in response to Eμ enhancer activity was already detected at a low level in multipotent progenitors (LSK), was then strongly increased in LMPPs and reached high levels in ALPs and BLPs (Fig 7B and C). These data therefore indicate that the Eμ enhancer is already highly active prior to B cell lineage commitment.

We next inserted the Eμ enhancer, with its 5′ and 3′ matrix attachment regions (MAR) and the downstream Iμ exon linked to a GFP gene, into the *Igh* ^∆890^ allele with the Floxin method to generate the *Igh* ^EμG^ allele (Fig 7D). Unexpectedly, GFP was expressed only in pro‐B cells of *Igh* ^EμG/+^ and *Igh* ^EμG/EμG^ mice, as it was not detected in uncommitted lymphoid progenitors, at later B cell developmental stages or in DN and DP T cells of these mice (Fig 7E–H). VDJ‐seq analysis furthermore revealed that the recombination frequency of the six most distal V_H_ gene was induced 3.5‐fold in *Igh* ^EμG/EμG^ pro‐B cells relative to *Igh* ^∆890/∆890^ pro‐B cells (Fig 7I and J). These data therefore demonstrate that the Eμ enhancer like the regulatory PAIR4‐V8.7E module can promote distal V_H_ gene recombination, when inserted in the 5′ region of the *Igh* locus, although at a somewhat lower frequency, which is consistent with the 1.8‐fold lower expression of *Gfp* mRNA in *Igh* ^EμG/EμG^ pro‐B cells relative to *Igh* ^P4GV/P4GV^ pro‐B cells (Fig EV5C). Like the PAIR4‐V8.7E module, the ectopic Eμ enhancer was also only active in pro‐B cells and thus behaved completely differently in this 5′ location as compared to its normal physiological position at the 3′ end of the *Igh* locus. These data strongly indicate that the 5′ region of the *Igh* locus may provide a special, but so far unknown environment that restricts the activity of the transplanted Eμ enhancer and possibly also the PAIR4‐V8.7E module specifically to the pro‐B cell stage during B lymphopoiesis.

## Discussion

Extended chromatin loop extrusion across the entire *Igh* locus is the overarching principle governing V_H_‐DJ_H_ recombination (Hill *et al*, 2020; Dai *et al*, 2021; Zhang *et al*, 2022) by facilitating convergent alignment of the RSS sequences of all V_H_ genes with the RSS element of the DJ_H_‐recombined segment in the 3′ proximal RAG^+^ recombination center prior to RAG‐mediated cleavage (Ji *et al*, 2010; Schatz & Ji, 2011). However, regulatory sequences within the V_H_ gene cluster, such as the PAIR elements (Ebert *et al*, 2011), may also contribute to the efficiency of V_H_‐DJ_H_ recombination. Here, we have shown that an 890‐kb deletion, which eliminated all 14 PAIRs and many other potential regulatory elements (Fig EV2D) in the distal V_H_ gene region, resulted in a strongly reduced recombination efficiency of V_H_ genes located up to 112 kb upstream or 194 kb downstream of the deletion point. Both total transcript and mRNA sequencing identified PAIR4 and PAIR6 as two highly active potential regulatory elements within the deleted region (Verma‐Gaur *et al*, 2012; Medvedovic *et al*, 2013; this study). By performing reconstitution experiments, we have now demonstrated that the PAIR4 and V8.7E sequences as well as the V8.8E element function as enhancers to promote distal V_H_ gene recombination over a 100‐kb region. Hence, local regulatory elements, such as the PAIR4‐V8.7E module and the V8.8E enhancer, can influence V_H_ gene recombination and thus contribute to the diversification of the BCR repertoire in the context of chromatin loop extrusion.

Among all V_H_ genes, the members of the V_H_8 gene family predominantly contain active histone marks at a relatively high level in pro‐B cells (Malin *et al*, 2010). Moreover, 11 of the 14 PAIR elements are associated with a V_H_8 gene, leading to the suggestion that the active chromatin regions at the PAIR element and its associated V_H_8 gene may function together as a regulatory unit (Ebert *et al*, 2011). By analyzing open chromatin, we identified new potential regulatory elements (V8E) that are located immediately downstream of the RSS sequence of most V_H_8 genes. The new elements V8.7E, V8.8E, and V8.xE are likely responsible for the generation of active chromatin (H3K4me2, H3K9ac, and H3K27ac) at the V_H_8‐7, V_H_8‐8, and V_H_8‐x genes (Ebert *et al*, 2011; this study). The active promoter mark H3K4me3 was, however, absent at these elements, suggesting that they function as enhancers, which was corroborated by the observed decrease of distal V_H_ gene recombination upon V8.7E deletion in *Igh* ^P4/P4^ pro‐B cells compared with *Igh* ^P4V/P4V^ pro‐B cells and by the activation of distal V_H_ gene recombination by the V8.8E enhancer in *Igh* ^V8‐8/V8‐8^ pro‐B cells. While PAIR4 on its own already functions as an enhancer of V_H_ gene recombination in forward and inverse orientation in *Igh* ^P4/P4^ and *Igh* ^P4inv/P4inv^ pro‐B cells, respectively, the collaboration of PAIR4 with V8.7E results in maximal recombination activity in *Igh* ^P4V/P4V^ pro‐B cells, thus indicating that both enhancers together constitute one regulatory module. The PAIR4‐V8.7E module and the V8.8E enhancer in the *Igh* locus appear to fulfill a similar role as the recently identified E34 and E88 enhancers in the *Igk* locus (Barajas‐Mora *et al*, 2019, 2023) and the E_VH_1 enhancer in the proximal V_H_ gene region of the *Igh* locus (Bhat *et al*, 2023), since these regulatory elements promote local V gene recombination in their respective Ig locus. Hence, fine‐tuning of the V gene repertoire depends on local enhancers of V gene recombination in both *Igh* and *Igk* loci.

What could be the molecular mechanism by which the PAIR4‐V8.7E and V8.8E enhancers activate distal V_H_ gene recombination? It has been shown that the RAG^+^ recombination center in the *Igh* 3′ region specifically interacts with transcriptionally active PAIR elements and V8 genes in the distal V_H_ gene region, including PAIR4, V_H_8‐7, and V_H_8‐8 (Dai *et al*, 2021). These interactions are mediated by extended loop extrusion that occurs across the entire *Igh* locus and is essential for the participation of all V_H_ genes in V_H_‐DJ_H_ recombination at the pro‐B cell stage (Hill *et al*, 2020; Dai *et al*, 2021). Recently, we have shown that this process generates one largely contiguous interaction zone at the *Igh* locus, where all the different sequences within the V_H_ gene region appear to interact with each other (Hill *et al*, 2023). As our 3C‐qPCR analysis did not identify a role for the inserted PAIR4‐V8.7E module in promoting its interaction with the distal V_H_ genes, we propose that the extended loop extrusion mechanism mediates these long‐range interactions, which allows the enhancers of the PAIR4‐V8.7E module to induce active chromatin at the distal V_H_ genes. As the interaction of an active promoter with the RAG^+^ recombination center is sufficient to stall loop extrusion in the absence of CTCF‐binding sites (Zhang *et al*, 2019), it is likely that the dwelling time of the PAIR4‐activated distal V_H_ genes at the RAG^+^ recombination center is increased, thus leading to their enhanced recombination.

The directionality of the PAIR4 element may also be explained by loop extrusion, which likely initiates at random positions in the V_H_ gene cluster and initially proceeds in a symmetrical manner until the cohesin ring interacts with a CTCF protein bound to the next upstream forward‐oriented CTCF‐binding site, which leads to stabilized binding of cohesin at this site (Li *et al*, 2020; Hill *et al*, 2023; Fig EV5D). Thereafter, asymmetrical loop extrusion reels the DNA of the downstream *Igh* regions into the loop until it is halted by a CTCF protein bound to a reverse CTCF‐binding site in convergent orientation at the IGCR1 or 3′CBE elements (Hill *et al*, 2023; Fig EV5D). This loop extrusion mechanism predicts that only the V_H_1 genes located upstream of the PAIR4 insertion can efficiently interact through loop extrusion with the inserted PAIR4 element and thus undergo increased V_H_1 gene recombination, as observed (Figs EV3D and EV5D). In contrast, the downstream V_H_1 genes should be unable to interact through this loop extrusion mechanism with the inserted PAIR4 element, which explains the observed reduced recombination of these V_H_1 genes (Figs EV3D and EV5D). Moreover, loop extrusion can also explain the reduced recombination efficiency of the upstream V_H_1 genes in *Igh* ^P4inv/P4inv^ pro‐B cells (Fig 3G and F), as the inversion of PAIR4 also inverts the orientation of the CTCF‐binding site of PAIR4 in *Igh* ^P4inv/P4inv^ pro‐B cells relative to *Igh* ^P4/P4^ pro‐B cells (Fig EV5E). The reverse CTCF‐binding site of PAIR4 can now form new stabilized loops with the forward CTCF‐binding sites in the upstream region (Rao *et al*, 2014; Fig EV5E), which interferes with prolonged loop extrusion beyond the inserted PAIR4 element and can thus prevent interaction of the upstream V_H_ genes with the RAG^+^ recombination center, as previously shown for the inversion of the 890‐kb V_H_ gene region and upon the insertion of an array of reverse CTCF‐binding sites (Hill *et al*, 2020).

Whether the 26‐kb lncRNA transcribed from PAIR4 has a function in promoting distal V_H_ gene recombination is difficult to assess in our reconstitution system. Insertion of the short spliced PAIR4‐derived transcript or *Gfp* cDNA in exon 2 of PAIR4 was equally compatible with maximal induction of distal V_H_ gene recombination in *Igh* ^P4V/P4V^ and *Igh* ^P4GV/P4GV^ pro‐B cells despite the different sequences of the two transcripts. Moreover, the higher expression of GFP in *Igh* ^P4∆CtcfGV/P4∆CtcfGV^ pro‐B cells compared to *Igh* ^P4V/P4V^ pro‐B cells did not result in increased recombination of distal V_H_ genes, indicating that different levels of the PAIR4‐derived transcript do not correlate with a corresponding change in recombination efficiency. Finally, truncating the transcription at the start of exon 1 in PAIR4 did not abolish distal V_H_ gene recombination but reduced their recombination efficiency in *Igh* ^P4StopG/P4StopG^ pro‐B cells relative to *Igh* ^P4/P4^ pro‐B cells. Based on these data, we conclude that the PAIR4‐derived transcript has a relatively minor effect on the function of PAIR4 as a long‐range recombination enhancer. In contrast to the PAIR4‐derived transcript, there are multiple examples of lncRNAs that play an essential role in controlling gene expression, such as the Xist RNA in X‐chromosome inactivation (Loda & Heard, 2019) and the ThymoD RNA in activating the T‐lineage commitment gene *Bcl11b* in early T cell development (Isoda *et al*, 2017).

By analyzing the expression of the PAIR4‐ and PAIR6‐derived transcripts in early B cell development, we previously demonstrated that the activity of PAIR4 and PAIR6 is strictly pro‐B cell‐specific (Ebert *et al*, 2011), which we have now further confirmed by total transcript analysis. As the binding of Pax5 to both PAIR elements is already lost at the next developmental stage in pre‐B cells, we argued that Pax5 is involved in the control of PAIR activity (Ebert *et al*, 2011). Using our reconstitution system, we now demonstrate that mutation of the Pax5‐binding site in PAIR4 resulted in a 2‐fold decrease of PAIR4‐derived GFP expression and in reduced activation of distal V_H_ gene recombination in *Igh* ^P4∆Pax5GV/P4∆Pax5GV^ pro‐B cells. However, this experiment may not have revealed the full function of Pax5 in controlling the activity of the PAIR4‐V8.7E module, as Pax5 could still mediate part of its regulation through the intact Pax5‐binding site in the V8.7E enhancer in *Igh* ^P4∆Pax5GV/P4∆Pax5GV^ pro‐B cells. Notably, *Pax5* ^−/−^ progenitors do not express the lncRNAs of PAIR4 and PAIR6 (Ebert *et al*, 2011) possibly due to their developmental arrest at an early lymphoid progenitor stage resembling the Pax5^−^ BLP. Given our finding that open chromatin at the PAIR4‐V8.7E modules is induced in a small fraction of Pax5^+^ BLPs during the BLP‐to‐pro‐B cell transition, it is conceivable that Pax5 may indirectly activate the PAIR4‐V8.7E module, possibly by relocating the *Igh* locus from the repressive compartment at the nuclear periphery to central euchromatic positions in committed pro‐B cells. However, the *Igh* locus is already located at central nuclear positions in the absence of Pax5 in *Pax5* ^−/−^ progenitors, which express *Ebf1*, or upon ectopic expression of Pax5 in double‐negative *Ikzf1* ^Pax5/+^ thymocytes, which leads to Pax5‐dependent activation of *Ebf1* (Fuxa *et al*, 2004). Hence, the relocation of the *Igh* locus from the repressive periphery to central positions in the nucleus appears to be controlled by the B cell‐specific transcription factor Ebf1 rather than by Pax5 (Fuxa *et al*, 2004).

The multifunctional transcription factor CTCF is well known for its architectural role in stabilizing cohesin‐dependent long‐range loops in the genome as well as for its insulating function in preventing communication between regulatory elements (Merkenschlager & Nora, 2016). While CTCF was originally identified as a transcriptional repressor based on reporter gene assays (Baniahmad *et al*, 1990; Filippova *et al*, 1996), acute degradation of CTCF combined with nascent transcript analysis has recently reinforced the notion that CTCF can also function as a repressor of transcription in a manner independent of its architectural function (Luan *et al*, 2022). Here, we have shown by functional analysis of the CTCF‐binding site of PAIR4 in reporter gene assays that CTCF can act as a transcriptional repressor to decrease PAIR4 activity in pro‐B and pre‐B cells. Downregulation of CTCF expression in pro‐B cells was previously shown to enhance lncRNA transcription from PAIR4 and PAIR6 (Degner *et al*, 2011). Consistent with this finding, mutation of the CTCF‐binding site in PAIR4 resulted in a 2‐fold increase of PAIR4‐derived GFP expression in *Igh* ^P4∆CtcfGV/P4∆CtcfGV^ pro‐B cells, although it did not affect the long‐range activation of distal V_H_ gene recombination by the PAIR4‐V8.7E enhancer module. Unexpectedly, loss of the CTCF‐binding site prevented the shutdown of transcription from the PAIR4 element in pre‐B and immature B cells and furthermore activated PAIR4‐derived transcription during T cell development in *Igh* ^P4∆CtcfGV/P4∆CtcfGV^ mice. Hence, the pro‐B cell‐specific activity of the PAIR4‐V8.7E module critically depends on CTCF binding to the PAIR4 element. Notably, our analysis has provided the first evidence that deletion of a single CTCF‐binding site has a strong effect on the developmental regulation of a control region in the distal V_H_ gene region. Previously, two CTCF‐binding sites in the intergenic IGCR1 region between the V_H_ and D_H_ clusters were shown to be essential for controlling the proper ordering and B‐lineage specificity of V(D)J recombination at the *Igh* locus (Guo *et al*, 2011). Moreover, recombination of the most 3′ proximal V_H_ genes is known to critically depend on a CTCF‐binding site located immediately downstream of their RSS elements, demonstrating that CTCF bound to these sites promotes their accessibility and interaction with the RAG^+^ recombination center (Jain *et al*, 2018).

The Eμ enhancer in its normal position at the 3′ end of the *Igh* locus is already activated in LMPPs and remains active throughout B lymphopoiesis. It plays an important role in V(D)J recombination by promoting D_H_‐J_H_ rearrangements in lymphoid progenitors (Perlot *et al*, 2005; Chakraborty *et al*, 2009). Upon insertion into the 5′ region of the *Igh* locus, the Eμ enhancer was able to promote distal V_H_ gene recombination and was exclusively active only in pro‐B cells in a similar manner as the PAIR4‐V8.7E module. As the Eμ enhancer does not contain a CTCF‐binding site, these data demonstrate that a second mechanism apart from CTCF‐mediated repression must be responsible for the pro‐B cell specificity of the distally located Eμ enhancer. At present, it is unclear what specific feature in the 5′ region of the *Igh* locus may cause the suppression of enhancer activity in lymphoid cells other than pro‐B cells. In this context, it is interesting to note that the *Igh* locus is anchored via its 5′ region in the repressive compartment at the nuclear periphery in pro‐T cells (Fuxa *et al*, 2004) as well as at the repressive centromeric chromatin in pre‐B cells (Roldán *et al*, 2005).

In summary, we identified the regulatory PAIR4‐V8.7E module and the V8.8E element as the first local enhancers in the distal V_H_ gene cluster that promote V_H_ gene recombination and thus contribute to the fine‐tuning of the V_H_ gene repertoire. Importantly, the distal V_H_ gene region contains additional V_H_8‐associated elements (Fig 1E) and many other sequence elements that are characterized by the presence of open chromatin and the active H3K4me2 and H3K27ac histone marks (Fig EV2D) similar to the PAIR4‐V8.7E module and the V8.8E enhancer. It is therefore likely that multiple local enhancers shape the pattern of V_H_ gene recombination and thus contribute to the diversification of the BCR repertoire in the context of chromatin loop extrusion.

## Materials and Methods

### Mice

The following mice were maintained on the C57BL/6 background: *Pax5* ^fl/fl^ mice (Horcher *et al*, 2001), *Rag2* ^−/−^ mice (Shinkai *et al*, 1992), *Igh* ^V8‐8/V8‐8^ mice (Hill *et al*, 2020), *Meox2* ^Cre/+^ mice (Tallquist & Soriano, 2000), *Rosa26* ^CreERT2/+^ mice (Seibler *et al*, 2003), *Rosa26* ^LSL‐YPF/+^ mice (Srinivas *et al*, 2001), transgenic *Vav‐*Cre mice (de Boer *et al*, 2003), transgenic Flpe mice (Rodriguez *et al*, 2000), and transgenic CAGGs‐Dre mice (Anastassiadis *et al*, 2009). All animal experiments were carried out according to valid project licenses, which were approved and regularly controlled by the Austrian Veterinary Authorities.

### Generation of the *Igh* ^∆890^ allele

Genetic alterations were introduced into the C57BL/6 *Igh* allele. The hybrid C57BL/6 × 129/Sv ES cell line A9 was used for homologous recombination in ES cells. The *Igh* ^Pgk1‐fl‐890‐fl^ allele was created by first inserting a *lox*P (fl) site at position 116,237,220 (mm9, Chr.12) into the middle of the V_H_ gene cluster, followed by insertion of a second *lox* (*lox*71) site (in the same orientation) at position 117,126,667 in the *Igh* 5′ region by ES cell targeting and subsequent injection into blastocysts to obtain *Igh* ^Pgk1‐fl‐890‐fl/+^ mice (Fig EV1C). The *Igh* ^Pgk1‐∆890^ allele was generated by Cre‐mediated deletion of the *lox*71/*lox*P‐flanked *Igh* region in *Meox2* ^Cre/+^ *Igh* ^Pgk1‐fl‐890‐fl/+^ mice. The *Igh* ^∆890^ allele was created by Flpe‐mediated deletion of the *frt*‐flanked Pgk1 promoter region in *Igh* ^Pgk1‐fl‐890‐fl/+^ mice expressing the Flpe transgene. The *Igh* ^∆890^ allele thus contained only a promoter‐less blasticidin resistance gene at the deletion point (Fig EV1C), which facilitated the subsequent insertion of different PAIR constructs by the Floxin method (Singla *et al*, 2010).

### Floxin‐mediated insertion of PAIR4 constructs and the Eμ enhancer into the *Igh* ^∆890^ allele

Blastocyts from *Igh* ^∆890/+^ *Rosa26* ^CreERT2/+^ mice on a hybrid C57BL/6 × 129/Sv background were used to generate *Igh* ^∆890/+^ *Rosa26* ^CreERT2/+^ ES cell lines, as described (Leeb *et al*, 2015). The different PAIR4 constructs (Figs 3A, 5A, 7D, and EV4C and D) were cloned between the FseI and BamHI or BspEI sites of the custom‐made Floxin exchange vector pL1L2 and were then transfected into *Igh* ^∆890/+^ *Rosa26* ^CreERT2/+^ ES cells using the FuGENE^®^ HD Transfection Reagent (Promega). CreER^T2^ activity was induced by 4‐hydroxytamoxifen to facilitate Cre‐mediated insertion of the PAIR4 sequences into the lox71 site at the deletion point (117,126,667/116,237,220; mm9, Chr.12) of the *Igh* ^∆890^ allele (Fig EV2A) by the Floxin method (Singla *et al*, 2010). The *rox*‐flanked *Actb*‐Bsd expression cassette, used for selection of the targeted ES cells, was deleted after germline transmission with the CAGGs‐Dre transgene, which left only one *frt* and one *rox* site in the targeted *Igh* locus (Fig EV2C). The mouse Eμ enhancer with its 5′ and 3′ matrix attachment regions (XbaI DNA fragment), which was linked in the Iμ exon to a Gfp reporter gene, was cloned into the exchange vector pL1L2 followed by Floxin‐mediated insertion at the deletion point into the *Igh* ^∆890^ allele in ES cells, as described above.

### Generation of the *Igh* ^CμCre^ allele

The *Igh* ^CμCre^ allele was generated by homologous recombination in the ES cell line A9 as described in detail in Fig EV5A. The *frt*‐flanked *Pgk1*‐*Neo* expression cassette, which was used for selection of the targeted ES cell clones, was deleted in *Igh* ^CμCre‐neo^ mice expressing the Flpe transgene. This and all other mutant strains were backcrossed to the C57BL/6 background.

### Antibodies

The following monoclonal antibodies were purchased from BD Biosciences, Thermo Fisher Scientific, BioLegend, or Miltenyi Biotec and were used for flow‐cytometric analysis of mouse bone marrow, spleen, and thymus: B220/CD45R (RA3‐6B2), CD4 (L3T4), CD8a (53–6.7), CD19 (1D3), CD25/IL‐2Rα (PC61), CD115/MCSF‐R (AFS98), CD117/Kit (2B8), CD127/IL‐7Rα (A7R34), CD135/Flt3 (A2F10.1), Gr1 (RB6‐8C5), IgD (11‐26c), IgM (II/41), IgM^b^ (AF6‐78), Sca1/Ly6A (D7), Ly6D (49H4), TCRβ (H57‐597), CD44 (IM7), CD90.2/Thy1.2 (30‐H12), IgM^a^ (MA‐69), CD3 (145‐2C11), CD11b (M1/70), NK1.1 (PK136), and Ly6C (HK1.4).

The following rabbit polyclonal antibodies were used for ChIP analysis: anti‐Pax5 Ab (affinity‐purified, directed against amino acids 17–145 (Adams *et al*, 1992)), anti‐CTCF Ab (07‐729, Sigma‐Aldrich), anti‐Rad21 Ab (ab992, Abcam), anti‐H3K4me2 (07‐030, Millipore), anti‐H3K4me3 Ab (pAb‐003‐050, Diagenode), anti‐H3K9ac (07‐352, Millipore), and anti‐H3K27ac (ab4729, Abcam).

### Definition of hematopoietic cell types by flow cytometry

Cell types were defined as follows in the bone marrow: LSK (Lin^−^Kit^hi^Sca1^hi^), LMPP (Lin^−^Kit^hi^Sca1^hi^CD135^+^), ALP (Lin^−^CD127^+^CD135^+^Ly6D^−^), BLP (Lin^−^CD127^+^CD135^+^Ly6D^+^), Pax5‐deficient progenitors (CD19^−^B220^+^Kit^+^Ly6D^+^), pro‐B cells (CD19^+^B220^+^IgM^−^IgD^−^Kit^+^CD25^−^), pre‐B cells (CD19^+^B220^+^IgM^−^IgD^−^Kit^−^CD25^+^), immature B cells (CD19^+^B220^+^IgM^+^IgD^−^), recirculating B cells (CD19^+^B220^+^IgD^+^), macrophages (CD115^+^Gr1^int^), granulocytes (Gr1^+^); in the thymus: DN T cells (CD4^−^CD8^−^CD90.2^+^), DP T cells (CD4^+^CD8^+^), CD4^+^ SP T cells (CD4^+^CD8^−^), CD8^+^ SP T cells (CD4^−^CD8^+^). Lineage cocktail (Lin^−^) contained anti‐TCRβ, CD3, Gr1, CD11b, CD19, Ly6C, and NK1.1 antibodies. Flow cytometry experiments and FACS sorting were performed on LSR Fortessa (BD Biosciences) and FACSAria III (BD Biosciences) machines, respectively. Flowjo software (Treestar) was used for data analysis.

### RT‐qPCR analysis of *Gfp* mRNA expression

Total RNA was prepared from ex vivo sorted pro‐B cells by using a semi‐automated RNA bead isolation method with Sera‐Mag SpeedBead Carboxylate‐Modified Magnetic Particles (Hydrophobic; GE Healthcare) run on the magnetic particle processor KingFisher Duo instrument (Thermo Fisher Scientific). The cDNA was synthesized using Oligo d(T)_18_ primer (NEB) and SuperScript® II Reverse Transcriptase (Thermo Fisher Scientific) in the presence of RNase inhibitor (Thermo Fisher Scientific). Transcripts of the *Gfp* gene were amplified by qPCR using primers located in different exons (Dataset EV2) and normalized against the *Tbp* mRNA.

### 3D DNA‐FISH analysis

*Igh* locus‐specific DNA probes were prepared from the BACs RP RP23‐340K14 (*Igh* 5′ end) and RP24‐275L15 (*Igh* 5′ end) by nick‐translation with dUTP‐Alexa568 and dUTP‐Alexa488 (Invitrogen), respectively. Ex vivo FACS‐sorted pro‐B cells and Pax5‐deficient progenitors were washed in PBS and then fixed onto poly‐L‐lysine‐coated slides for two‐color 3D DNA‐FISH analysis as described in detail (Fuxa *et al*, 2004; Roldán *et al*, 2005). In short, cells were fixed in 2% PFA and permeabilized in 0.4% Triton. After blocking in 2.5% BSA, 0.1% Tween, and 10% goat serum, cells were incubated with RNaseA, permeabilized in 0.7% Triton and 0.1 M HCl and denatured. Hybridization with the labeled DNA probes was performed overnight, followed by washing and mounting in ProLong™ Gold Antifade Mountant (Invitrogen) mixed with 1.5 μg/ml DAPI. Image acquisition was performed by confocal microscopy on a Zeiss LSM780 system with GaAsP detector technology. Optical sections separated by 0.25 μm were collected with a 63× objective (63×/1.4 plan‐apochromat Oil DIC). Cells with signals from both alleles were analyzed by the Imaris (Bitplane) software.

### ChIP analysis of transcription factors and histone modifications

Pro‐B cells, which were short‐term cultured on OP9 cells in IL‐7‐containing IMDM (Nutt *et al*, 1997), were crosslinked with 1% formaldehyde (Sigma) for 10 min. Nuclei were prepared and lysed in the presence of 0.25% SDS (Pax5, CTCF) or 1% SDS (histone modifications). The chromatin was sheared by sonication with the Bioruptor® Standard (Diagenode), followed by immunoprecipitation with a polyclonal anti‐Pax5 paired domain Ab (Adams *et al*, 1992) or a polyclonal anti‐CTCF Ab (07–729, Sigma‐Aldrich; Fig EV4E and G). The specific enrichment, which was measured by qPCR analysis with primers amplifying the Pax5‐ or CTCF‐binding site of PAIR4, *Nedd9* and *Bud13* or a control sequence in a gene‐poor region of chromosome 1 (Dataset EV2), was calculated as the amount of precipitated DNA relative to input DNA.

Native ChIP analysis of the H3K4me2, H3K4me3, and H3K9ac modifications (Fig EV3E) was performed by fixing short‐term cultured pro‐B cells (5 × 10^7^) with acetone at −20°C prior to nucleosome extraction as described (Fursova *et al*, 2019). In brief, the nuclei were released by resuspension in lysis buffer (10 mM Tris‐HCl pH 8.0, 10 mM NaCl, 3 mM MgCl_2_, 3 mM CaCl_2_, 0.1% Igepal). The chromatin was digested with 100 units of micrococcal nuclease (MNase, Fermentas EN0181) in MNase digestion buffer (0.25 M sucrose, 10 mM Tris–HCl pH 8.0, 10 mM NaCl, 3 mM MgCl_2_, 0.1% Igepal, 1× EDTA‐free cOmplete protease inhibitor cocktail) for 5 min at 37°C. The nucleosomes were further extracted by incubating the nuclei in nucleosome release buffer (10 mM Tris–HCl pH 7.5, 10 mM NaCl, 0.2 mM EDTA, 1× EDTA‐free cOmplete) for 1 h at 4°C. ChIP was carried out by incubating 40 ng of chromatin in ChIP incubation buffer (10 mM Tris‐HCl pH 7.5, 70 mM NaCl, 2 mM MgCl_2_, 2 mM EDTA, 0.1% Triton X‐100, 1× EDTA‐free cOmplete) with anti‐H3K4me2, anti‐H3K4me3 or anti‐H3K9ac antibodies overnight at 4°C. The immune complexes were captured on Protein G Mag Sepharose® Xtra (Cytiva 28‐9670‐70), washed four times with N‐ChIP wash buffer (10 mM Tris–HCl pH 7.5, 125 mM NaCl, 2 mM EDTA, 0.1% Triton X‐100) and once with TE buffer (10 mM Tris–HCl pH 8.0, 1 mM EDTA), followed by release in DNA extraction buffer (100 mM Tris, pH 8.0, 5 mM EDTA, 0.1% SDS, 200 mM NaCl, 20 μg/ml Proteinase K) overnight at 50°C. The DNA was isolated using carboxyl magnetic beads (produced in‐house) and analyzed by qPCR amplification.

### Electrophoretic mobility shift assay

Nuclear extracts were prepared from the human B cell line Ramos as described (Decker *et al*, 2009). One of the two strands of the double‐stranded DNA probe, which contains the Pax5‐binding sites of PAIR4, was ordered as a 6‐FAM‐labeled oligonucleotide (IDT). The labeled probe (20 fmoles) was incubated with 6 μg of nuclear extract in 20 μl of binding buffer (10 mM HEPES pH 7.9, 55 mM KCl, 3 mM MgAc, 1 mM DTT, 4% Ficoll, 250 μg/ml BSA, 0.1 μg/μl poly[d(I‐C)]) in the presence or absence of specific competitor DNA or anti‐Pax5 antibody for 30 min at room temperature. Protein‐DNA complexes were separated on a 4% polyacrylamide gel in 0.25× TBE buffer (22 mM Tris‐borate pH 8.3, 0.5 mM EDTA) at 100 V for 2–3 h at room temperature. Gels were subjected to fluorography using the ChemiDoc MP Imaging System (Bio‐Rad Laboratories). The oligonucleotides used for EMSA analysis are listed in Dataset EV2.

### Transient transfection assays

The luciferase reporter gene pXPG‐P4 was generated by linking the entire PAIR4 sequence with PAIR4 exon 1 and 2 to the promoter‐less firefly luciferase gene present in plasmid pXPG (Bert *et al*, 2000; shown in Fig EV4I). The mutant CTCF‐binding site (shown in Fig EV4B) was introduced to generate pXPG‐P4∆CTCF while a wild‐type CTCF‐binding site was additionally inserted upstream of PAIR4 in the construct pXPG‐CTCF‐P4∆CTCF. Transient transfections of cells of the pro‐B cell line 38B9 (Alt *et al*, 1984) and the pre‐B cell line PD31 (Lewis *et al*, 1982) were performed six times in triplicate using the Lipofectamine 2000 reagent (Invitrogen). Pro‐B and pre‐B cells (2 × 10^5^) were co‐transfected with the firefly luciferase construct (40 ng) and control vector pRL‐CMV (0.2 ng; Promega) expressing the Renilla luciferase gene under the control of the CMV enhancer and promoter. After 24 h, the cells were lysed, and luciferase activities were measured using the Dual‐Glo Luciferase Assay System (Promega) in the Synergy 2 microplate reader (Biotek). The firefly luciferase activities were normalized to the control Renilla luciferase activity.

### 3C‐qPCR analysis

The 3C‐templates were prepared from 5–10 × 10^5^ sorted pro‐B cells (CD19^+^B220^+^Kit^+^CD25^−^IgM^−^IgD^−^) from the bone marrow or double‐positive (DP) T cells (CD19^−^CD90.2^+^CD4^+^CD8^+^) from the thymus by DpnII digestion of the chromatin followed by religation, as previously described (Oudelaar *et al*, 2017). Independently prepared 3C‐templates were subjected to quantitative TaqMan PCR analysis using the Luna Universal Probe qPCR Master Mix (New England Biolabs). As an internal control for the quality of the 3C template, the ubiquitously expressed *Ercc3* (XPB) locus was also analyzed by qPCR (Splinter *et al*, 2006). The crosslinking frequencies, determined for the V_H_1.81, V_H_1.82, V_H_1.84, and V_H_1.86 genes as well as for the control *Ercc3* locus, were calculated by using DpnII‐digested and randomly ligated BAC DNA spanning the respective distal *Igh* region or the *Ercc3* locus as a standard for PCR amplification. The relative crosslinking frequency was determined as the ratio of the crosslinking frequency measured at the distal V_H_ genes relative to the crosslinking frequency determined at the *Ercc3* gene. The oligonucleotides used for 3C‐qPCR analysis are listed in Dataset EV2.

### Total RNA‐sequencing

Pro‐B and pre‐B cells were sorted from the bone marrow of 3‐5‐week‐old mice. Total RNA was isolated using a lysis step based on guanidine thiocyanate and was then further processed on the KingFisher Flex Magnetic Particle Processor. The semi‐automated procedure included a 15‐min DNase I digest at 37°C and a 5‐min elution in H_2_O at 60°C. RNA integrity and concentration were assessed by a fragment analyzer. Depletion of rRNA from the total RNA was performed by using a mix of antisense oligonucleotides matching mouse rRNA and the Hybridase Thermostable RNase H (Epicenter), which specifically degrades RNA in RNA–DNA hybrids, as described in detail (Batki *et al*, 2019). DNA was subsequently digested with TURBO DNase (Invitrogen), and RNA was purified using RNA Clean & Concentrator‐5 (Zymo) according to the manufacturer's instructions. Libraries were prepared using a NEBNext Ultra Directional RNA Library Prep Kit for Illumina (NEB) according to the manufacturer's protocol. Paired‐end 50‐bp sequencing was performed on a NextSeq3000 sequencing instrument.

### cDNA preparation for RNA‐sequencing

RNA from *ex vivo*‐sorted immature B cells was isolated with the RNeasy Plus Mini Kit (Qiagen). mRNA was obtained by two rounds of poly(A) selection using the Dynabeads mRNA purification kit (Invitrogen) and fragmented by heating at 94°C for 3 min in fragmentation buffer. The fragmented mRNA was used as a template for first‐strand cDNA synthesis with random hexamers using the Superscript Vilo First‐Strand Synthesis System (Invitrogen). The second‐strand cDNA was synthesized with 100 mM dATP, dCTP, dGTP, and dUTP in the presence of RNase H, *E. coli* DNA polymerase I, and DNA ligase (Invitrogen).

### Library preparation and Illumina deep sequencing

About 0.5–5 ng of cDNA or ChIP‐precipitated DNA were used as starting material for the generation of sequencing libraries with the NEBNext Ultra II DNA library prep kit for Illumina (NEB). Alternatively, sequencing libraries were generated using the NEBNext End Repair/dATailingModule and NEBNext Ultra Ligation Module (NEB), followed by amplification with the KAPA Real‐Time Amplification kit (KAPA Biosystems). Cluster generation and sequencing were carried out using the Illumina HiSeq 2000/2500 system according to the manufacturer's guidelines. Dataset EV3 provides further information about all sequencing experiments of this study.

### VDJ‐seq analysis

VDJ‐Seq analysis of the *Igh* locus was performed as described (Chovanec *et al*, 2018). Genomic DNA was extracted from *ex vivo*‐sorted pro‐B cells. The DNA (2 μg) was sheared using the Bioruptor sonicator (Diagenode) and subjected to end‐repair and A‐tailing, followed by ligation of adapters containing 12 UMI sequences using the NEBNext Ultra II DNA library prep kit for Illumina (NEB). A primer extension step with biotinylated J_H_‐specific primers generated the single‐stranded DNA products that were captured using Dynabeads MyOne streptavidin T1 beads (Thermo Fisher Scientific) and PCR‐amplified with nested J_H_‐specific and adapter‐binding primers (Chovanec *et al*, 2018). The llumina sequencing adapter primers, including the indexes for multiplexing of libraries, were added to the PCR products in a final PCR amplification step. Paired‐end 300‐bp sequencing was performed on a MiSeq (Illumina) sequencing instrument (Dataset EV3).

### Bioinformatic analysis of VDJ‐seq data

The bioinformatic analysis of the VDJ‐seq data was performed as described in detail (Chovanec *et al*, 2018), and the resulting data was displayed using scripts based on R version 3.3.3.

### Bioinformatic analysis of ChIP‐seq data

All sequence reads that passed the Illumina quality filtering were considered for alignment after adapter trimming. Reads were aligned against the *Mus musculus* genome version of July 2007 (NCBI37/mm9) with the Bowtie program version 0.12.5 (Langmead *et al*, 2009). Read coverages were visualized with the UCSC genome browser (Kuhn *et al*, 2013).

### Bioinformatic analysis of RNA‐seq data

Only reads that passed the Illumina quality filtering were processed. Reads were filtered against rDNA with the Bowtie 2 program (Langmead & Salzberg, 2012).

For total RNA‐seq, the remaining reads were aligned with the STAR program version 2.4.2 (Dobin *et al*, 2013) to the mm9 genome version of July 2007 (NCBI37). Read coverages were calculated with the BEDTool program (Quinlan & Hall, 2010), were normalized to reads per million (RPM) using SAMTools (Li *et al*, 2009) as well as KentTools (Kuhn *et al*, 2013) and were visualized with the UCSC genome browser (Kuhn *et al*, 2013).

For mRNA‐seq, the remaining reads were aligned with the TOPHAT program version 1.4.1 (Trapnell *et al*, 2009) using transcriptome‐guided alignment. Only uniquely mapping reads, as marked by MarkDuplicates (PICARD program version 2.6.0, McKenna *et al*, 2010), were used for counting. Reads were counted using the featureCounts program version 1.5.0 (Liao *et al*, 2014), over all genes of the transcriptome (Revilla‐i‐Domingo *et al*, 2012) including the immunoglobulin genes incorporated from the Ensembl release 67 (Flicek *et al*, 2013). TPM values were calculated as described (Wagner *et al*, 2012).

### Multiple sequence alignments of V_H_8 and V_H_1 sequences

V_H_8 and V_H_1 gene sequences, including 400 bp of their downstream regions, were analyzed based on their previously described mm10 genomic coordinates (Proudhon *et al*, 2015; Dataset EV1). Two additional V_H_8 genes, that were not annotated in the mm10 genome, were included in the analysis: V_H_8‐x (previous annotation: V_H_3609.8pg.160 close to PAIR6) with the mm10 coordinates chr12:115404420‐115404865 and V_H_8‐y (previous annotation: V_H_3609.14pg.181 close to PAIR13) with the mm10 coordinates chr12:115808428‐115808860. The DNA sequences were aligned with the MUSCLE version 3.8.31 (Edgar, 2004). Reverse complement conversion and the alignment display were done with emboss tools version 6.6.0. Sequence identities were calculated with R/Bioconductor (Gentleman *et al*, 2004) and Biostrings modules (Pagès *et al*, 2022), and the data were plotted with ggplot (https://ggplot2.tidyverse.org).

### Statistical analysis

Statistical analysis was performed with the GraphPad Prism 7 software. Two‐tailed unpaired Student's *t*‐test analysis was used to assess the statistical significance of one observed parameter between two experimental groups. If more than one parameter was measured in two experimental groups, multiple *t*‐tests were applied, and the Holm‐Sidak multicomparison test was used to report the significance between the two groups. One‐way ANOVA was used when more than two experimental groups were compared, and the statistical significance was determined by the Tukey *post hoc* test.

## Disclosure and competing interests statement

The authors declare that they have no conflict of interest.

## Supporting information



Expanded View Figures PDF

Dataset EV1

Dataset EV2

Dataset EV3

Source Data for Expanded View

PDF+

Source Data for Figure 2

Source Data for Figure 3

Source Data for Figure 4

Source Data for Figure 5

Source Data for Figure 6

Source Data for Figure 7

## Data Availability

The VDJ‐seq, ChIP‐seq, and ATAC‐seq data reported in this study (Dataset EV3) are available at the Gene Expression Omnibus (GEO) repository under the accession number GSE214869 (https://www.ncbi.nlm.nih.gov/geo/query/acc.cgi?acc=GSE214869).
