# Supplementary material for: Enhancers of the PAIR4 regulatory module promote distal VH gene recombination at the Igh locus
Source: EMBO J. 2023 Jun 20;42(15):e112741. doi: 10.15252/embj.2022112741 (PMC10390877; doi:10.15252/embj.2022112741)
Supplement: Supplementary file 1 — Expanded View Figures PDF [file EMBJ-42-e112741-s001.pdf]

Expanded View Figures

Figure EV1. Characterization of the PAIR4, PAIR6, and V8E elements.

A, B Presence of active histone marks, transcription factor binding, open chromatin, and transcription in the PAIR4 (A) and PAIR6 (B) regions at the pro-B cell stage. Open chromatin was furthermore mapped by ATAC-seq in hCD2^{-} (Pax5^{-}) and hCD2^{+} (Pax5^{+}) BLPs that were sorted from the bone marrow of *Pax5*$^{ihCd2/ihCd2}$ mice (Fuxa & Busslinger, 2007; Hill *et al*, 2020). The RPM scales for displaying the open chromatin peaks in BLPs and pro-B cells were adjusted to show equal densities of the open chromatin peaks present at a gene-dense genomic region, including the ubiquitously expressed *Tbp* locus. The location of PAIR4, PAIR6, and their associated V_H8 genes are shown together with the exons of the PAIR4-derived lncRNA (RIKEN clone CJ056205; below) and the mm9 genomic coordinates of mouse chromosome 12 (above). As the previously identified V_H3609.8pg.160 (Johnston *et al*, 2006) was not mapped in the mouse mm9 or mm10 genome, we refer to this PAIR6-associated gene as V_H8-x. The coordinates for the V_H8-x gene are chr12:116642631-116643076 (mm9) or chr12:115404420–115,404,865 (mm10). Gray overlay indicates the DNA sequences of the PAIR4-V8.7E module that were inserted with the Floxin method at the deletion point of the *Igh*$^{\Delta 890}$ allele to generate the *Igh*^{P4V} allele (Fig 3A). RPM, reads per million mapped sequence reads.

C, D Sequence conservation of the aligned V_H8 (C) and V_H1 (D) gene regions and their 3′ flanking sequences (~400 bp). The line denotes the LOESS-smoothed position-wise maximum sequence identity across the multiple sequence alignments. The coding sequences of the V_H genes are indicated together with the recombination signal sequences (RSS).

E Sequence alignment of the ~200-bp conserved region downstream of the 3′ end of the V_H8 genes. Dots indicate identical nucleotides relative to the consensus sequence shown below. Nonidentical nucleotides are shown by their respective letters (C, G, T, and A). Gaps in the alignment are indicated by dashes. Numbers refer to the positions downstream of the V_H8 3′ end.

 © 2023 The Authors

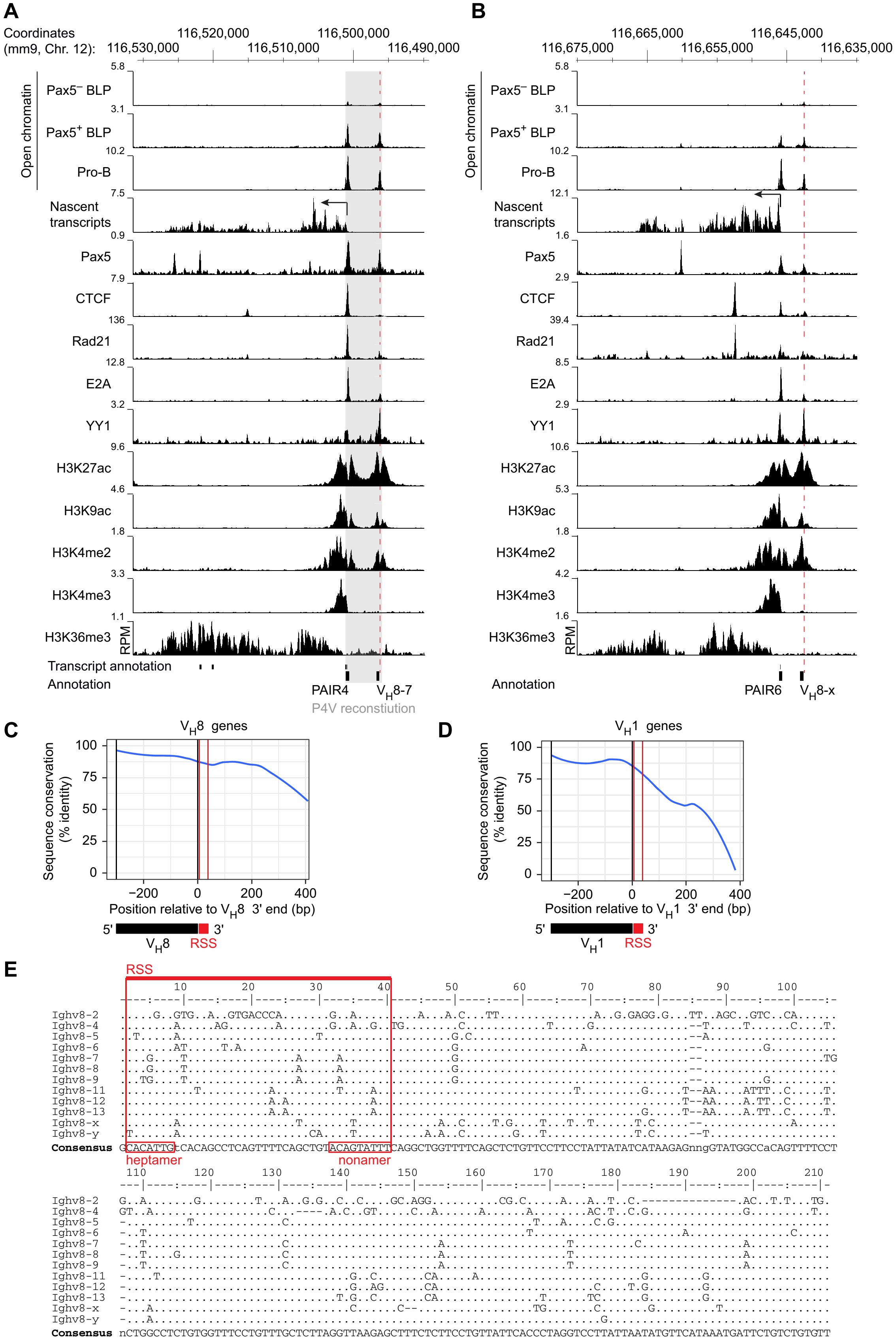
A
Coordinates
(mm9, Chr. 12):
116,530,000
116,520,000
116,510,000
116,500,000
116,490,000
Open chromatin
Pax5⁻ BLP
Pax5⁺ BLP
Pro-B
Nascent transcripts
Pax5
CTCF
Rad21
E2A
YY1
H3K27ac
H3K9ac
H3K4me2
H3K4me3
H3K36me3
RPM
Transcript annotation
Annotation
PAIR4
VH8-7
P4V reconstiution
B
116,675,000
116,665,000
116,655,000
116,645,000
116,635,000
PAIR6
VH8-x
C
VH8 genes
Sequence conservation (% identity)
Position relative to VH8 3' end (bp)
5'
3'
VH8
RSS
D
VH1 genes
Position relative to VH1 3' end (bp)
VH1
RSS
E
RSS
heptamer
nonamer
Ighv8-2
Ighv8-4
Ighv8-5
Ighv8-6
Ighv8-7
Ighv8-8
Ighv8-9
Ighv8-11
Ighv8-12
Ighv8-13
Ighv8-x
Ighv8-y
Consensus

Figure EV1.

Figure EV2. Generation, characterization, and reconstitution of the *Igh*$^{\Delta 890}$ allele and presence of potential regulatory elements throughout the V_H gene cluster.

A Generation of the *Igh*$^{\Delta 890}$ allele. The indicated selection cassettes were used for introducing the upstream *lox*71 site and the downstream *lox*P site at the specified positions of the *Igh* locus by sequential ES cell targeting to generate the *Igh*$^{Pgk1\text{-}fl\text{-}890\text{-}fl}$ allele. The *Igh*$^{\Delta 890}$ allele was subsequently generated by sequential deletion of the indicated sequences by Cre- and Flpe-mediated recombination *in vivo* in the mouse, as described in Materials and Methods. Actb, human β-actin promoter; Bsd, blasticidin resistance gene; Neo, neomycin resistance gene; Puro, puromycin resistance gene; Pgk1, phosphoglycerate kinase-1 promoter.

B Location of the V_H1 gene family members, which undergo rearrangements in *Igh*$^{+/+}$ pro-B cells and exhibit strongly reduced recombination (indicated by the red line; see Fig 2D) upon deletion of the 890-kb region in the 5′ region of the *Igh* locus. The distance of the different V_H genes from the deletion point is shown in kilobases (kb).

C Schematic diagram of the Floxin system used for inserting the different PAIR4 constructs and the Eμ enhancer at the deletion point of the *Igh*$^{\Delta 890}$ allele in ES cells. See Materials and Methods for detailed description of the reconstitution experiments.

D Presence of active chromatin across the *Igh* locus in *Rag2*$^{-/-}$ pro-B cells. Open chromatin was mapped by ATAC-seq, while the presence of H3K27ac and H3K4me2 peaks was identified by ChIP-seq analysis with anti-H3K27ac and anti-H3K4me2 antibodies. The peaks corresponding to PAIR and V8E elements are highlighted by orange or black lines, respectively. The Eμ enhancer and 3′CBE region are indicated in red. The annotation of the C57BL/6 *Igh* locus with the different V_H gene families (different colors), the D_H (light blue), and C_H (blue) elements is shown together with the extent of the 890-kb deletion (black line) and the mm9 genomic coordinates of mouse chromosome 12.

 © 2023 The Authors

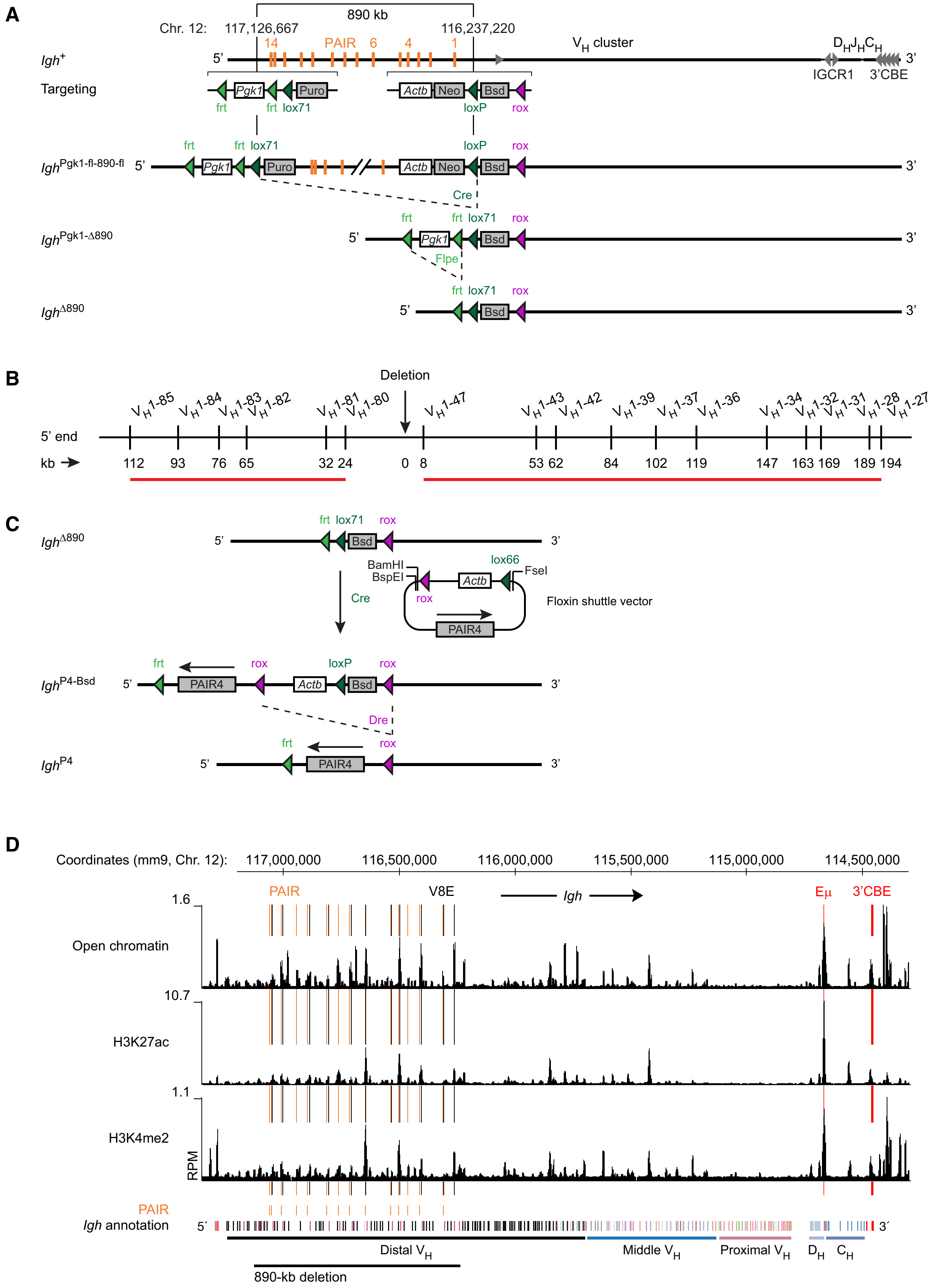
A
890 kb
Chr. 12: 117,126,667 116,237,220
14 PAIR 6 4 1
VH cluster
DHJHCH
Igh+ 5' 3'
IGCR1 3'CBE
Targeting
Pgk1 Puro Actb Neo Bsd
frt frt lox71 loxP rox
IghPgk1-fl-890-fl
Cre
IghPgk1-Δ890
Flpe
IghΔ890
B
Deletion
VH1-85 VH1-84 VH1-83 VH1-82 VH1-81 VH1-80 VH1-47 VH1-43 VH1-42 VH1-39 VH1-37 VH1-36 VH1-34 VH1-32 VH1-31 VH1-28 VH1-27
5' end
kb
112 93 76 65 32 24 0 8 53 62 84 102 119 147 163 169 189 194
C
IghΔ890
frt lox71 rox
BamHI
BspEI
lox66
FseI
Cre
Floxin shuttle vector
PAIR4
IghP4-Bsd
loxP
Dre
IghP4
D
Coordinates (mm9, Chr. 12):
117,000,000 116,500,000 116,000,000 115,500,000 115,000,000 114,500,000
PAIR V8E Igh Eμ 3'CBE
Open chromatin
H3K27ac
H3K4me2
RPM
1.6 10.7 1.1
PAIR
Igh annotation
Distal VH Middle VH Proximal VH DH CH
890-kb deletion

Figure EV2.

Figure EV3. Activation of distal V_H gene recombination upon reconstitution of the *Igh*$^{\Delta 890}$ allele with different PAIR4 constructs.

A V_H gene expression from the *Igh*$^{P4V(B6)}$ and *Igh*$^{\Delta 890(B6)}$ alleles in immature IgM^{b} (B6) B cells sorted from the bone marrow of *Igh*$^{P4V(B6)/+(129)}$ and *Igh*$^{\Delta 890(B6)/+(129)}$ mice, respectively (see also Fig 3B). The expression (TPM) value of each V_H gene is shown as shared (light gray) or unique expression of the *Igh*$^{P4V(B6)}$ allele (dark gray) or Igh$^{\Delta 890(B6)}$ allele (white bars). The expression data are shown as mean TPM values and are based on three or two independent RNA-seq experiments for the immature B cells of *Igh*$^{P4V(B6)/+(129)}$ and *Igh*$^{\Delta 890(B6)/+(129)}$ mice, respectively. The different V_H genes (horizontal axis) are aligned according to their position in the *Igh* locus (Dataset EV1). The 890-kb deletion is indicated by a black line. TPM, transcripts per million; B6, C57BL/6 strain; 129, 129/Sv strain.

B VDJ-seq analysis of *Igh* rearrangements in pro-B cells of the indicated genotypes. The percentages of uniquely identified DJ_H and VDJ_H sequences are shown as mean percentage with SEM. The high variance caused by the many data points obtained with control *Igh*$^{+/+}$ pro-B cells is likely responsible for the fact that only the comparison between *Igh*$^{+/+}$ and *Igh*$^{\Delta 890/\Delta 890}$ pro-B cells reached statistical significance.

C Normalized recombination frequency of the first six distal V_H genes (V_H1-85 to V_H1-80) determined in pro-B cells of the indicated genotypes, based on the data shown in Fig 3D and G. The recombination frequency of each of the six distal V_H genes was calculated as a mean value with SEM, and the value obtained with Igh$^{\Delta 890/\Delta 890}$ pro-B cells was set to 1 (dashed line).

D The PAIR4-V8.7E module and PAIR4 elements differentially activate the recombination of V_H genes located upstream or downstream of their insertion. The normalized recombination frequency of the first six upstream V_H genes (V_H1-85 to V_H1-80) and the first six downstream V_H genes (V_H1-47, V_H1-43, V_H1-42, V_H1-39, V_H1-37, V_H1-36; Fig EV2B) was determined in pro-B cells of the indicated genotypes, based on the data shown in Fig 3D and G. The average recombination frequency of the six upstream and six downstream V_H genes was calculated as mean value with SEM, and the value obtained with *Igh*$^{\Delta 890/\Delta 890}$ pro-B cells was set to 1.

E Active chromatin at the V_H1-81 and V_H1-82 genes in *Igh*$^{P4V/P4V}$, *Igh*$^{\Delta 890/\Delta 890}$ and *Igh*$^{+/+}$ pro-B cells. Short-term cultured pro-B cells of the indicated genotypes were used for native ChIP analysis with anti-H3K9ac, anti-H3K4me2 and anti-H3K4me3 antibodies. Input and precipitated DNA were quantified by qPCR analysis with primers amplifying the sequences of the V_H1-81, V_H1-82 and *Tbp* genes. The amount of precipitated DNA was calculated as mean percentage of input with SEM and was normalized to the value obtained for the positive *Tbp* control. The relative enrichment is shown by setting the normalized value of the control *Igh*$^{+/+}$ pro-B cells to 1.

F 3C-qPCR analysis of the long-range interactions between the PAIR4-V8.7E module and distal V_H genes in sorted bone marrow pro-B cells and CD4^{+}CD8^{+} double-positive (DP) thymocytes from *Igh*$^{P4V/P4V}$ and *Igh*$^{\Delta 890/\Delta 890}$ mice. The relative crosslinking frequencies between the reference DpnII fragment (viewpoint, located downstream of the insertion site) and the indicated distal V_H genes were determined with independently prepared 3C-templates and were normalized relative to the crosslinking frequency measured for the control *Ercc3* gene (see Materials and Methods). The mean crosslinking frequency with SEM is shown for each V_H gene by setting the mean value obtained with DP T cells for each genotype to 1.

G Absence of GFP expression in pro-B cells of *Igh*$^{P4StopG/+}$ (light green) and *Igh*$^{P4StopG/P4StopG}$ (green) mice compared with control *Igh*$^{+/+}$ (black) mice, as indicated by flow-cytometric analysis and geometric mean fluorescence intensity measurement. Geometric mean fluorescence intensity measurements are shown as a mean value with SEM.

Data information: Statistical data (B, D, E, F, G) were analyzed by one-way ANOVA (Tukey *post hoc* test); *$P < 0.05$, **$P < 0.01$, ***$P < 0.001$, ****$P < 0.0001$. Each dot (B-G) corresponds to one mouse.
Source data are available online for this figure.

 © 2023 The Authors

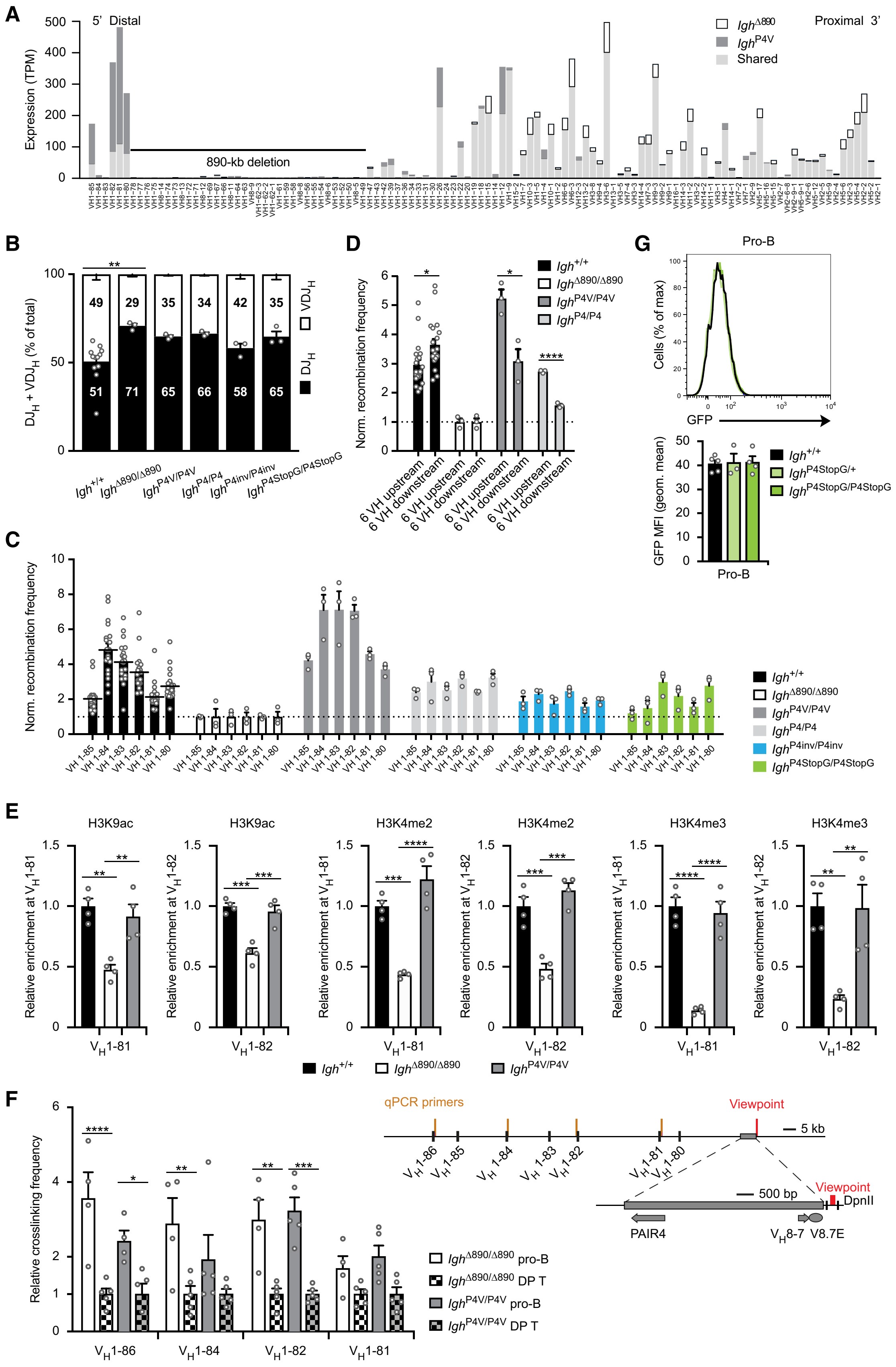

Figure EV3.

Figure EV4. Mutation of the Pax5- and CTCF-binding sites in the PAIR4 element.

A Mutation of the Pax5-binding sequence of PAIR4. The nucleotides of PAIR4, which match the Pax5 consensus recognition sequence (Kaiser *et al*, 2022), are underlined and were mutated to the nucleotides indicated in red (ΔPax5).

B Mutation of the CTCF-binding sequence of PAIR4. The nucleotides of PAIR4, corresponding to the consensus CTCF-binding motif (Hill *et al*, 2020), are underlined and were mutated to the nucleotides indicated in red (ΔCTCF).

C, D Schematic diagram of the *Gfp*-linked PAIR4-V8.7E module with the Pax5- or CTCF-binding site mutation, which was used for the generation of the $Igh^{P4\Delta Pax5GV}$ or $Igh^{P4\Delta CtcfGV}$ allele, respectively.

E, G Pax5 and CTCF binding to the wild-type and mutant PAIR4 sequences. Short-term cultured pro-B cells from the bone marrow of $Igh^{P4GV/P4GV}$ (green), $Igh^{P4\Delta Pax5GV/P4\Delta Pax5GV}$ (yellow), and $Igh^{P4\Delta CtcfGV/P4\Delta CtcfGV}$ (brown) mice were used for ChIP analysis with an anti-Pax5 paired domain antibody or an anti-CTCF antibody. Input and precipitated DNA were quantified by qPCR analysis with primers amplifying the Pax5-binding site present in PAIR4 or the *Nedd9* gene (E), the CTCF-binding site present in PAIR4 or the *Bud13* gene (G) or by amplifying a gene-poor region on chromosome 1 as a negative control (Dataset EV2). The amount of precipitated DNA was calculated as an average percentage of input with SEM and is shown relative to the value obtained for the negative control.

F No binding of Pax5 to the mutant Pax5 recognition sequence (ΔPax5) of PAIR4. A fluorescently-labeled double-stranded oligonucleotide containing the wild-type (wt) Pax5 recognition sequence of PAIR4 (shown below) was used as a probe for electrophoretic mobility shift assay (EMSA) with a nuclear extract prepared from B cells of the human Ramos cell line (see Materials and Methods). The Pax5-DNA complex (marked as Pax5 to the left) was not formed upon addition of an anti-Pax5 antibody, directed against the DNA-binding paired domain (Prd), or in the presence of a 10-, 30-, and 100-fold molar excess of a nonlabeled competitor oligonucleotide containing the wild-type Pax5-binding site in contrast to the competitor oligonucleotide containing the mutant (mut) Pax5 recognition sequence (shown below).

H Absence of GFP expression in BLPs from the bone marrow of $Igh^{P4GV/+}$ (green), $Igh^{P4\Delta Pax5GV/+}$ (yellow), $Igh^{P4\Delta CtcfGV/+}$ (brown), and control $Igh^{+/+}$ (black) mice, as shown by flow-cytometric analysis.

I Transient transfection reporter assay. The PAIR4-luciferase reporter genes (schematically shown to the left) were transfected together with the control vector pRL-CMV into cells of the pro-B cell line 38B9 (Alt *et al*, 1984) or pre-B cells line PD31 (Lewis *et al*, 1982). After 24 h, luciferase activities were measured, normalized and displayed relative to the activity of the parental vector pXPG, containing a promoter-less firefly luciferase gene, which was set to 1. The relative luciferase (luc) activities of six independent transfection experiments are shown as mean values with SEM. Ex1 and Ex2 refer to exons 1 and 2 of the PAIR antisense transcript.

Data information: Statistical data (E, G, I) were analyzed by one-way ANOVA (Tukey *post hoc* test); ns $P > 0.05$, $^{*}P < 0.05$; $^{**}P < 0.01$, $^{****}P < 0.0001$.
Source data are available online for this figure.

 © 2023 The Authors

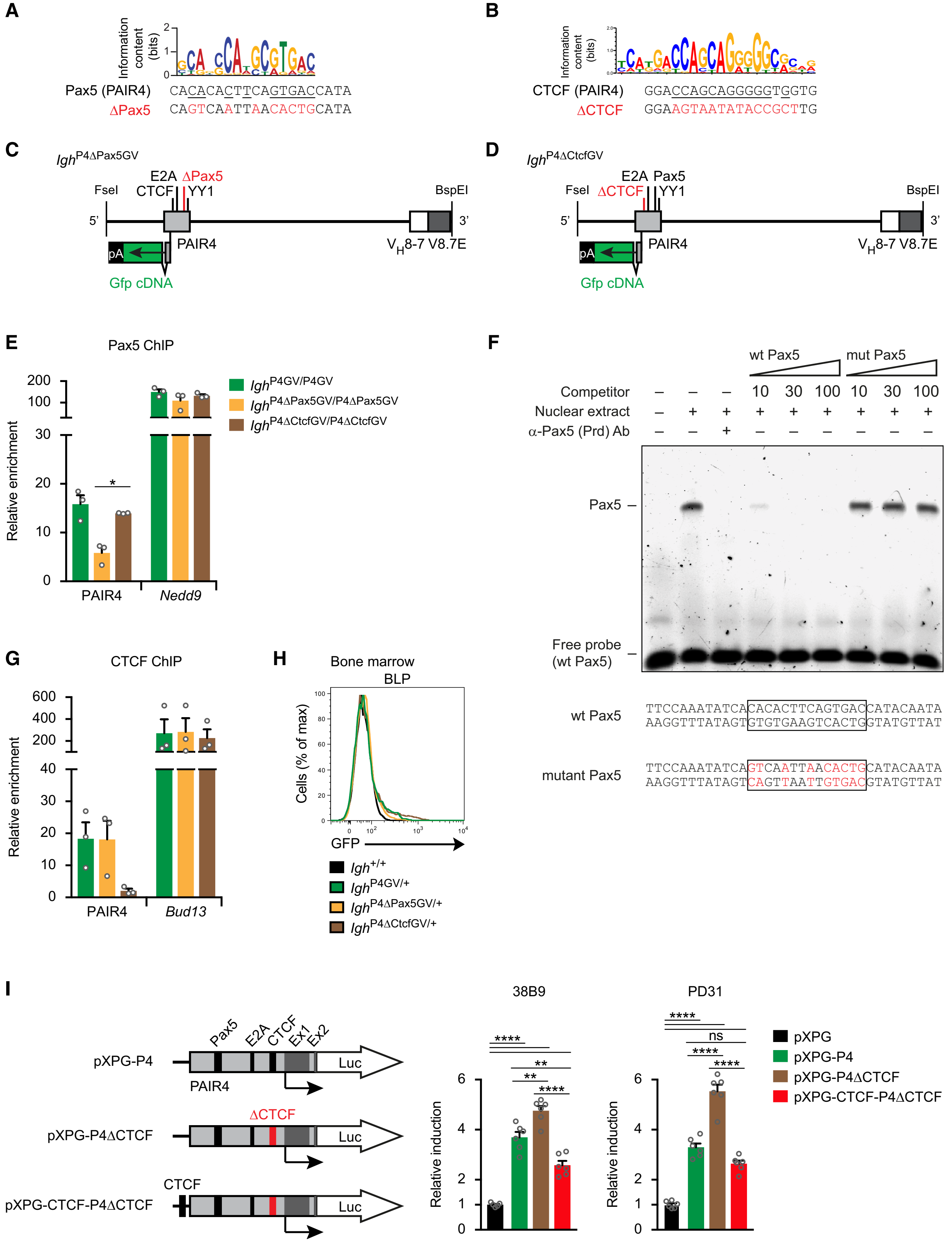
A
Information content (bits)
Pax5 (PAIR4) CACACACTTCAGTGACCATA
ΔPax5 CAGTCAATTAACACTGCATA
B
Information content (bits)
CTCF (PAIR4) GGACCAGCAGGGGGTGGTG
ΔCTCF GGAAGTAATATACCGCTTG
C
Igh P4ΔPax5GV
E2A ΔPax5
FseI CTCF YY1
BspEI
5' 3'
pA PAIR4 VH8-7 V8.7E
Gfp cDNA
D
Igh P4ΔCtcfGV
E2A Pax5
FseI ΔCTCF YY1
BspEI
pA PAIR4 VH8-7 V8.7E
Gfp cDNA
E
Pax5 ChIP
Relative enrichment
PAIR4 Nedd9
Igh P4GV/P4GV
Igh P4ΔPax5GV/P4ΔPax5GV
Igh P4ΔCtcfGV/P4ΔCtcfGV
F
wt Pax5 mut Pax5
Competitor – – – 10 30 100 10 30 100
Nuclear extract – + + + + + + + +
α-Pax5 (Prd) Ab – – + – – – – – –
Pax5 –
Free probe (wt Pax5) –
wt Pax5 TTCCAAATATCACACACTTCAGTGACCATACAATA
AAGGTTTATAGTGTGTGAAGTCACTGGTATGTTAT
mutant Pax5 TTCCAAATATCAGTCAATTAACACTGCATACAATA
AAGGTTTATAGTCAGTTAATTGTGACGTATGTTAT
G
CTCF ChIP
Relative enrichment
PAIR4 Bud13
H
Bone marrow BLP
Cells (% of max)
GFP
Igh +/+
Igh P4GV/+
Igh P4ΔPax5GV/+
Igh P4ΔCtcfGV/+
I
Pax5 E2A CTCF Ex1 Ex2
pXPG-P4 Luc
PAIR4
ΔCTCF
pXPG-P4ΔCTCF Luc
CTCF
pXPG-CTCF-P4ΔCTCF Luc
38B9
PD31
Relative induction
pXPG
pXPG-P4
pXPG-P4ΔCTCF
pXPG-CTCF-P4ΔCTCF

Figure EV4.

Figure EV5. Generation and characterization of the *Igh*$^{C\mu Cre}$ allele and explanation of recombination differences in the context of loop extrusion.

A Generation of the *Igh*$^{C\mu Cre}$ allele by ES cell targeting. The *Igh*$^{C\mu Cre\text{-}neo}$ allele was generated by homologous recombination in the ES cell line A9 by using the following targeting vector. The targeting vector consisted of a 4.1-kb long 5′ homology region (containing Cμ1 to CμS), a *frt*-flanked 2.1-kb DNA fragment containing the mouse phosphoglycerate kinase (*Pgk1*) promoter linked to the neomycin (Neo) resistance gene and a SV40 polyadenylation signal, a 1.6-kb DNA fragment containing the CμM1/2 sequence linked in frame via the P2A peptide to an iCre gene followed by a 1.8-kb long 3′ homology region. The *frt* sites are indicated by green arrowheads. The ApaI fragments of the *Igh*$^{+}$ and *Igh*$^{C\mu Cre\text{-}neo}$ alleles, which were used for allele identification by Southern blot analysis with the indicated probe, are shown together with their length (in kilobases, kb). The *Igh*$^{C\mu Cre}$ allele was generated by deletion of the *frt*-flanked *Pgk1-Neo* expression cassette, which was used for selection of the targeted ES cell clones, in *Igh*$^{C\mu Cre\text{-}neo}$ mice expressing the Flpe transgene.

B Southern blot analysis of wild-type (WT) ES cells and correctly targeted ES cell clones by hybridization of ApaI-digested genomic DNA with the probe indicated in (A). The ES cell clone 11F was injected into blastocysts to generate the *Igh*$^{C\mu Cre\text{-}neo/+}$ mouse strain.

C RT-qPCR analysis of *Gfp* mRNA expression in sorted pro-B cells from the bone marrow of the indicated mouse strains. The transcripts of the *Gfp* gene were normalized against the control *Tbp* mRNA, and the mean value obtained with *Igh*$^{+/+}$ pro-B cells was set to 1. *Gfp* mRNA expression is shown as a mean value with SEM based on nine (*Igh*$^{+/+}$) or four (*Igh*$^{P4GV/P4GV}$, *Igh*$^{E\mu G/E\mu G}$, *Igh*$^{P4StopG/P4StopG}$) independent samples. Statistical data were analyzed by one-way ANOVA (Tukey *post hoc* test); ****$P < 0.0001$.

D Role of loop extrusion in mediating increased recombination of V_H genes located upstream of the insertion of the PAIR4-V8.7E module (Figs 3G and EV3D). The loop extrusion process has been shown to generate a largely contiguous interaction zone where all the different sequences within the V_H gene region appear to interact with each other (Hill *et al*, 2023). The generation of this interaction zone requires that all CTCF-binding sites are present in forward orientation in the V_H gene cluster (Hill *et al*, 2020). Loop extrusion likely initiates at random positions in the V_H gene cluster and initially proceeds in a symmetrical manner until the cohesin ring interacts with a CTCF protein bound to the next upstream forward-oriented CTCF-binding site, which leads to stabilized binding of cohesin at this site (Li *et al*, 2020). Thereafter, asymmetrical loop extrusion reels the DNA of the downstream *Igh* regions into the loop, until it is halted by a CTCF protein bound to a reverse-oriented CTCF-binding site at the IGCR1 or 3′CBE elements (Hill *et al*, 2023). This loop extrusion mechanism predicts that only the V_H1 genes located upstream of the PAIR4 insertion can efficiently interact through loop extrusion with the inserted PAIR4 element and thus undergo increased V_H1 gene recombination. In contrast, the downstream V_H1 genes are unable to interact through this loop extrusion mechanism with the inserted PAIR4 element, which results in reduced V_H1 gene recombination.

E Explanation for the reduced recombination efficiency of upstream V_H1 genes in *Igh*$^{P4inv/P4inv}$ pro-B cells compared with *Igh*$^{P4/P4}$ pro-B cells (Fig 3G and F). The inversion of PAIR4 also inverts the orientation of the CTCF-binding site of PAIR4 in *Igh*$^{P4inv/P4inv}$ pro-B cells relative to *Igh*$^{P4/P4}$ pro-B cells. The reverse-oriented CTCF-binding site of PAIR4 is now in convergent orientation relative to the forward CTCF-binding sites in the upstream region and can thus form new stabilized loops (Rao *et al*, 2014) that interfere with prolonged loop extrusion beyond the inserted PAIR4 element and can therefore prevent interactions of the upstream V_H genes with the RAG^{+} recombination center in the *Igh* 3′ region, as previously shown for the inversion of the 890-kb V_H gene region (Hill *et al*, 2020). The forward and reverse CTCF consensus motifs (D, E) are shown.

Source data are available online for this figure.

 © 2023 The Authors

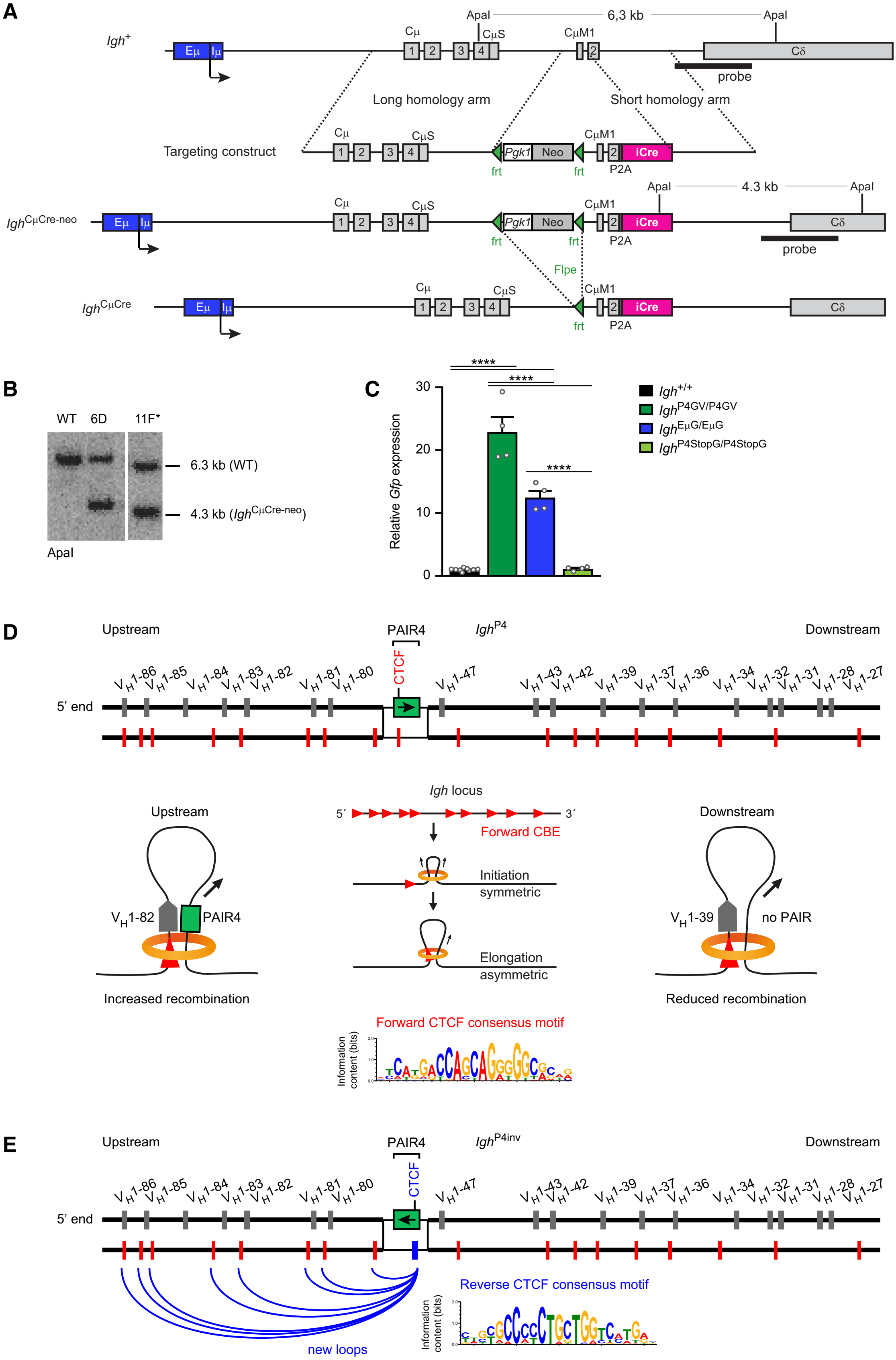
A
Igh+
Eμ Iμ
Cμ
1 2 3 4
CμS
ApaI
6,3 kb
ApaI
CμM1
Cδ
probe
Long homology arm
Short homology arm
Targeting construct
Pgk1 Neo
frt
iCre
P2A
IghCμCre-neo
4.3 kb
IghCμCre
Flpe
B
WT 6D 11F*
6.3 kb (WT)
4.3 kb (IghCμCre-neo)
ApaI
C
Relative Gfp expression
****
Igh+/+
IghP4GV/P4GV
IghEμG/EμG
IghP4StopG/P4StopG
D
Upstream
PAIR4
IghP4
Downstream
CTCF
5' end
VH1-86 VH1-85 VH1-84 VH1-83 VH1-82 VH1-81 VH1-80 VH1-47 VH1-43 VH1-42 VH1-39 VH1-37 VH1-36 VH1-34 VH1-32 VH1-31 VH1-28 VH1-27
Igh locus
Forward CBE
Initiation
symmetric
Elongation
asymmetric
VH1-82
PAIR4
Increased recombination
VH1-39
no PAIR
Reduced recombination
Forward CTCF consensus motif
Information content (bits)
E
IghP4inv
new loops
Reverse CTCF consensus motif

Figure EV5.
