# Supplementary material for: Enhancers of the PAIR4 regulatory module promote distal VH gene recombination at the Igh locus
Source: EMBO J. 2023 Jun 20;42(15):e112741. doi: 10.15252/embj.2022112741 (PMC10390877; doi:10.15252/embj.2022112741)
Supplement: Supplementary file 13 [file EMBJ-42-e112741-s003.pdf]

THE EMBO JOURNAL

Enhancers of the PAIR4 regulatory module promote distal V_H gene recombination at the Igh locus

Louisa Hill, Markus Jaritz, Hiromi Tagoh, Karina Schindler, Daniela Kostanova-Poliakova, Qiong Sun, Tanja Schwickert, Martin Leeb and Meinrad Busslinger
DOI: 10.15252/embj.2022112741

Corresponding author(s): Meinrad Busslinger (Meinrad.Busslinger@imp.ac.at)

Review Timeline:

Submission Date:	4th Oct 22
Editorial Decision:	11th Oct 22
Appeal Received:	24th Oct 22
Editorial Decision:	21st Nov 22
Revision Received:	4th May 23
Editorial Decision:	18th May 23
Revision Received:	22nd May 23
Accepted:	2nd Jun 23

Editor: Stefanie Boehm / Karin Dumstrei

Transaction Report:

(Note: With the exception of the correction of typographical or spelling errors that could be a source of ambiguity, letters and reports are not edited. Depending on transfer agreements, referee reports obtained elsewhere may or may not be included in this compilation. Referee reports are anonymous unless the Referee chooses to sign their reports.)

Prof. Meinrad Busslinger
Research Institute of Molecular Pathology (IMP)
Campus-Vienna-Biocenter 1
Vienna, Vienna 1030
Austria

11th Oct 2022

Re: EMBOJ-2022-112741
Enhancers of the PAIR4 regulatory module promote distal VH gene recombination at the Igh locus

Dear Prof. Busslinger,

Thank you for submitting your manuscript (EMBOJ-2022-112741) to The EMBO Journal. I have now read your study carefully and discussed the work with the other members of the editorial team. In addition, I also consulted an external expert on your study and further discussed these comments as well as the manuscript with my colleague Karin Dumstrei. However, taking everything into consideration, we have unfortunately decided that we cannot offer to send the study out for peer-review at The EMBO Journal, at this time.

We appreciate that you identify novel regulatory elements (V8E) downstream of the V(H)8 genes and perform further analysis of the PAIR4-V8.7E module and V8.8E element, which you find enhances recombination of six most distal V(H) genes and induces active chromatin. Moreover, you report that module activity is regulated by Pax5 and CTCF, where Pax5 binding is required for maximal activity of PAIR4 in pro-B cells, while CTCF ensure the pro-B cell specificity. We recognize that the identification of the enhancer element promoting long-distance activation of V(H) gene recombination, will be of interest to the field. However, at the same time, we also find that this process would need to further defined on a molecular level.

Our advisor, who is familiar with the field as well as the journal and its scope, came to a similar conclusion. S/he acknowledged the interest in the topic and that this was the first report of "recombination enhancers in the V portion of Igh". However, despite also noting that the additional characterization of the PAIR element and region was of interest to the field, our advisor was also concerned about the depth of mechanistic understanding. S/he concluded that it is shown that enhancer elements in the V portion of antigen receptor loci exist, but not how they work. For example that it is "unclear how PAIR-VH elements stimulate recombination. Is it by activating germline transcription of the V segments, which then creates a local speed bump? Or by altering locus topology?". In addition, our advisor was also not convinced by the Pax5 binding site deletion analyses and noted that the effects of deleting the CTCF binding site were interesting, but the mechanism not explored.

Thus, taking everything into consideration, we have concluded that the mechanistic insight currently provided is not sufficient to warrant further consideration for publication at The EMBO Journal. However, as our advisor, we also appreciate the interest of your findings to the field. We noticed that you selected the 'no transfer' option on the submission form, but we would like to strongly encourage you to consider a transfer of the manuscript to our sister journal EMBO reports for peer-review in its current form. If you decide to do so, you can use the link provided below, no reformatting is required. Please also do not hesitate to contact me to set up a meeting in case you would like to discuss the possible ways forward in more detail.

Thank you again for giving us the opportunity to consider your manuscript. I am sorry that we cannot be more positive on this occasion.

Kind regards,

Stefanie Boehm

Stefanie Boehm
Editor
The EMBO Journal

*** As a service to authors, The EMBO Journal offers the possibility to directly transfer declined manuscripts to another EMBO Press title (EMBO Reports, EMBO Molecular Medicine, Molecular Systems Biology) or to the open access journal Life Science Alliance launched in partnership between EMBO Press, Rockefeller University Press and Cold Spring Harbor Laboratory Press. The full manuscript (including reviewer comments, where applicable and if chosen) will be automatically forwarded to the receiving journal, to allow for fast handling and a prompt decision on your manuscript. For more details of this service, and to

transfer your manuscript to another EMBO title please follow this link:
Link Not Available

Research Institute of Molecular Pathology
Campus-Vienna-Biocenter 1
1030 Vienna, Austria
T +43-1-79730-3150
www.imp.ac.at

Stefanie Böhm, PhD
Editor
The EMBO Journal

Prof. Dr. Meinrad Busslinger
Scientific Deputy Director
busslinger@imp.ac.at

Vienna, 17 October 2021

Dear Dr. Böhm,

Many thanks for your careful evaluation of our manuscript and for involving an external advisor in this process. We appreciate your positive appraisal of the novelty of our data. Your decision not to send our manuscript out for review is largely based on the argument that our manuscript lacks mechanistic insight into how PAIR4 and the novel V8.7E and V8.8 enhancers mediate distal V_H gene recombination. The important question here is what the term 'mechanistic insight' means in the context of our manuscript. The external advisor mentioned several aspects of potential mechanistic insight, which I will briefly discuss below.

Activation of germline transcription by the novel enhancers. The advisor rightly points out that we should have investigated whether the PAIR4-V8.7E module promotes distal V_H gene recombination by activating their germline transcription. So far, we have shown that the PAIR4-V8.7E module induces active chromatin at the distal V_H genes. We strongly suspect that germline transcription will also be induced by this module. We would be happy to perform the germline transcript analysis and provide these data in a revised manuscript version.

Pax5-binding site mutation. As shown by ChIP-seq analysis, Pax5 binding to PAIR4 is 3-fold reduced, but not abrogated by extensive mutation of the Pax5-binding site (Fig EV4F), which apparently did not convince the external advisor. On page 14 of the manuscript, we explicitly mention that the partial loss of Pax5 binding could be caused by a second weaker Pax5-binding site in adjacent PAIR4 sequences. Importantly however, the Pax5-binding site mutation reduced the PAIR activity by a factor of 2 (Fig 6A,B) and distal V_H gene recombination by a factor of 1.4 in the respective pro-B cells (Fig 6G), clearly indicating that Pax5 contributes to PAIR4 activity.

Function of the CTCF-binding site in PAIR4. On page 22 of the discussion section, we mention in detail that CTCF bound to PAIR4 may function as an insulator to down-tune the activity of PAIR4. We could provide mechanistic insight into this insulator function by analyzing the role of the CTCF-binding site in controlling PAIR activity in transient transfection experiments. We would be willing to provide these data in a revised manuscript version.

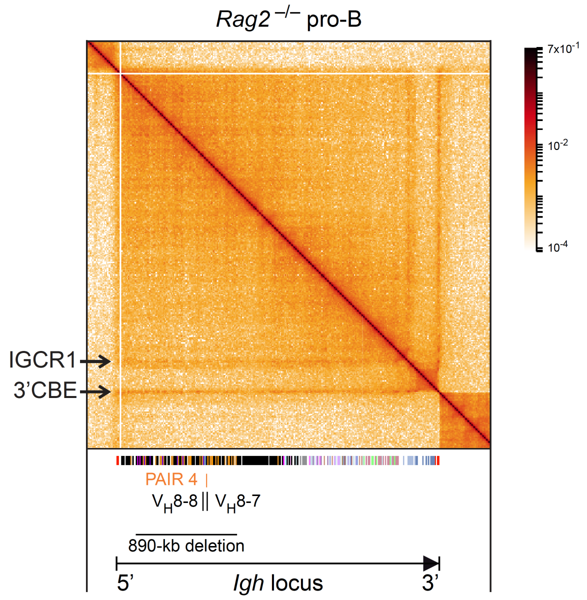

Figure 1. Contact matrix of the *Igh* locus, as determined by Micro-C analysis of *Rag2*$^{-/-}$ pro-B cells (Hill et al., 2022, Nat. Commun., in revision). While all CTCF-binding sites in the V_H gene cluster are present in forward orientation, the reversely oriented CTCF-binding sites in the IGCR1 and 3'CBE regions are able to stabilize chromatin loops, which is indicated by the contact stripes emanating from these two elements. The extent of the 890-kb deletion is shown together with the positions of the PAIR4 element as well as the V_H8-7 and V_H8-8 genes.

Altering of locus topology - long-range interactions between novel enhancers and distal V_H genes.
As enhancers are known to regulate their target genes through chromatin looping, it is an important mechanistic aspect to demonstrate the long-range interactions of an enhancer with its target genes. However, this is precisely not possible within the *Igh* locus for the following reason. We have recently shown that extended loop extrusion is the overarching principle that promotes V_H-to-DJ_H recombination at the *Igh* locus in pro-B cells (Hill et al., 2020, Nature 584, 142-147). Extended loop extrusions across the entire V_H gene cluster generates one contiguous interaction zone, where all the different sequences interact with each other. This is best shown by the contact matrix of the *Igh* locus that we determined by high-resolution Micro-C analysis (Figure 1). As there is no pronounced specificity of the interactions within the V_H gene cluster, the extended loop extrusion should also mediate the long-range interactions between the PAIR4-V8.7E module and the six distal V_H gene (V_H1-85 to V_H1-80) analyzed. However in this situation, it is not possible to experimentally verify a specific interaction between these elements by 3C-sequencing, as no sequence in the V_H gene cluster could serve as negative control. In a revised manuscript, we will mention this special feature of the *Igh* locus in detail in the discussion section.

In addition, I would like to point out that all experiments of our manuscript were performed with *in vivo* B cell types directly isolated from the mouse, as we strongly believe that the results obtained with *in vivo* B cells are closer to reality compared with those obtained with *in vitro* cultured B cell lines. For this purpose, we have generated multiple mouse strains by genetic engineering.

As you suggested in your email, I would be happy to further discuss with you these and other arguments that might be in favor of sending our manuscript out for review at the EMBO Journal.

I am looking forward to hearing from you.

With best regards,

Meinrad Busslinger

Meinrad Busslinger

Prof. Meinrad Busslinger
Research Institute of Molecular Pathology (IMP)
Campus-Vienna-Biocenter 1
Vienna, Vienna 1030
Austria

21st Nov 2022

Re: EMBOJ-2022-112741R-Q
Enhancers of the PAIR4 regulatory module promote distal VH gene recombination at the Igh locus

Dear Prof. Busslinger, dear Meinrad,

Thank you for submitting an updated version of your manuscript (EMBOJ-2022-112741) to The EMBO Journal. As we agreed upon, we sent the revised manuscript with the additional discussion to three experts. We have now received their comments (copied below) and in light of the overall positive feedback, we now invite you to prepare and submit a revised manuscript for The EMBO Journal.

As you will see, all referees appreciate the findings and acknowledge their interest to the field. However, they do raise several points that should be addressed in the revised manuscript, many of which can likely be resolved by revising the text and adding to the discussion. This includes further expanding on the aspect of locus chromatin topology, which had also been an issue brought up by the initial expert advisor, as well as taking into account the comments of referee #2 (point 2) and referee #3 (point 4) regarding a potential orientation bias. In addition, please clarify the statements on PAX5 and CTCF binding (ref #1- point 2; ref #2- point 4, minor 2; ref #3- point 5, 6, 7). Finally, please also carefully consider all other referee comments and revise the manuscript and figures as needed, as well as providing a detailed response to each comment. Please also remember that the revised manuscript should fulfill all EMBO Journal formatting requirements when it is next submitted (please see below and: https://www.embopress.org/page/journal/14602075/authorguide#submissionofrevisions)

Please note that it is our policy to allow only a single round of major revision. Acceptance depends on a positive outcome of a second round of review and therefore on the completeness of your responses included in the next, final version of the manuscript. In case any issues or questions come up, please contact us as soon as possible to discuss how to best proceed.

Thank you for the opportunity to consider your work for publication. I look forward to receiving your revised manuscript.

Kind regards,

Stefanie Boehm

Stefanie Boehm
Editor
The EMBO Journal

*** PLEASE NOTE: All revised manuscript are subject to initial checks for completeness and adherence to our formatting guidelines. Revisions may be returned to the authors and delayed in their editorial re-evaluation if they fail to comply to the following requirements (see also our Guide to Authors for further information):

1) Every manuscript requires a Data Availability section (even if only stating that no deposited datasets are included). Primary datasets or computer code produced in the current study have to be deposited in appropriate public repositories prior to resubmission, and reviewer access details provided in case that public access is not yet allowed. Further information: embopress.org/page/journal/14602075/authorguide#dataavailability

2) Each figure legend must specify
- size of the scale bars that are mandatory for all micrograph panels
- the statistical test used to generate error bars and P-values
- the type error bars (e.g., S.E.M., S.D.)
- the number (n) and nature (biological or technical replicate) of independent experiments underlying each data point
- Figures may not include error bars for experiments with n<3; scatter plots showing individual data points should be used instead.

3) At EMBO Press we ask authors to provide source data for the main and EV figures. Our source data coordinator will contact you to discuss which figure panels we would need source data for and will also provide you with helpful tips on how to upload and organize the files.

When submitting your revised manuscript, please carefully review the instructions that follow below. Failure to include requested items will delay the evaluation of your revision.***

-

When submitting your revised manuscript, please carefully review the instructions below and include the following items:

1) A .docx formatted version of the manuscript text (including legends for main figures, EV figures and tables). Please make sure that the changes are highlighted to be clearly visible.

2) Individual production quality figure files as .eps, .tif, .jpg (one file per figure). *Please remove figures from the main manuscript*

3) A .docx formatted letter INCLUDING the reviewers' reports and your detailed point-by-point response to their comments. As part of the EMBO Press transparent editorial process, the point-by-point response is part of the Review Process File (RPF), which will be published alongside your paper.

4) A complete author checklist, which you can download from our author guidelines (https://wol-prod-cdn.literatumonline.com/pb-assets/embo-site/Author%20Checklist%20-%20EMBO%20J-1561436015657.xlsx). Please insert information in the checklist that is also reflected in the manuscript. The completed author checklist will also be part of the RPF.

5) All corresponding authors are required to supply an ORCID ID for their name upon submission of a revised manuscript.

6) Before submitting your revision, primary datasets produced in this study need to be deposited in an appropriate public database. The required format for this section is:

Data availability

The datasets (and computer code) produced in this study are available in the following databases:

[data type]: [name of the resource] [accession number/identifier/doi] ([URL or identifiers.org/DATABASE:ACCESSION])

*** Note - All links should resolve to a page where the data can be accessed. ***

Please also see https://www.embopress.org/page/journal/14602075/authorguide#datadeposition Please note that the Data Availability Section is restricted to new primary data that are part of this study.

7) We replaced Supplementary Information with Expanded View (EV) Figures and Tables that are collapsible/expandable online (see examples in http://msb.embopress.org/content/11/6/812). A maximum of 5 EV Figures can be typeset. EV Figures should be cited as 'Figure EV1, Figure EV2" etc.. in the text and their respective legends should be included in the main text after the legends of regular figures.

- For the figures that you do NOT wish to display as Expanded View figures, they should be bundled together with their legends in a single PDF file called *Appendix*, which should start with a short Table of Content. Appendix figures should be referred to in the main text as: "Appendix Figure S1, Appendix Figure S2" etc. See detailed instructions regarding expanded view here: < https://www.embopress.org/page/journal/14602075/authorguide#expandedview>.

- Additional Tables/Datasets should be labeled and referred to as Table EV1, Dataset EV1, etc. Legends have to be provided in a separate tab in case of .xls files. Alternatively, the legend can be supplied as a separate text file (README) and zipped together with the Table/Dataset file.
** We suggest uploading table S1, S2, S3 as datasets. **

8) Our journal encourages inclusion of *data citations in the reference list* to directly cite datasets that were re-used and

obtained from public databases. Data citations in the article text are distinct from normal bibliographical citations and should directly link to the database records from which the data can be accessed. In the main text, data citations are formatted as follows: "Data ref: Smith et al, 2001" or "Data ref: NCBI Sequence Read Archive PRJNA342805, 2017". In the Reference list, data citations must be labeled with "[DATASET]". A data reference must provide the database name, accession number/identifiers and a resolvable link to the landing page from which the data can be accessed at the end of the reference. Further instructions are available at < https://www.embopress.org/page/journal/14602075/authorguide#referencesformat >.

Additional information on source data and instruction on how to label the files are available at < https://www.embopress.org/page/journal/14602075/authorguide#sourcedata >.

10) When assembling figures, please refer to our figure preparation guideline in order to ensure proper formatting and readability in print as well as on screen:
https://wol-prod-cdn.literatumonline.com/pb-assets/embo-site/EMBOPress_Figure_Guidelines_061115-1561436025777.pdf

Please remember: Digital image enhancement is acceptable practice, as long as it accurately represents the original data and conforms to community standards. If a figure has been subjected to significant electronic manipulation, this must be noted in the figure legend or in the 'Materials and Methods' section. The editors reserve the right to request original versions of figures and the original images that were used to assemble the figure. The editors reserve the right to request original versions of figures and the original images that were used to assemble the figure. Finally, we generally encourage uploading of numerical as well as gel/blot image source data; for details see: embopress.org/page/journal/14602075/authorguide#sourcedata

Further information is available in our Guide For Authors:
https://www.embopress.org/page/journal/14602075/authorguide

****
In the interest of ensuring the conceptual advance provided by the work, we recommend submitting a revision within 3 months (19th Feb 2023). Please discuss the revision progress ahead of this time with the editor if you require more time to complete the revisions. Use the link below to submit your revision:

Link Not Available
****

Referee #1:

The manuscript "Enhancers of the PAIR4 regulatory module promote distal VH gene
recombination at the Igh locus" by Hill et al. provides a detailed analysis on the function of PAIR4 element in regulation of VH sequence usage in VDJ recombination during early B cell development. The authors show that PAIR4 element, particularly together with a newly identified enhancer element in the immediate vicinity of a V8-7 gene segment, can rescue the deletion of a larger region within the VH region lacking all 14 PAIR elements in promoting distal VH usage. They also show that the use of distal VH gene segments can be rescued with other enhancers (V8-8 with its respective enhancer and with the well characterized Emu enhancer). The findings are novel, build on their previous findings on the PAIR element. The findings presented in the manuscript are important in understanding the mechanism and fine tuning of the antibody repertoire formation. The antibody repertoire formation has some relevance for antibody response to vaccination and is associated with human disease.

The work is carried out with great scrutiny, uses ingenious mouse models, and the conclusions drawn from the data are appropriate. It would have been curious to learn how the investigated elements affect the chromatin structure as this would have given greater mechanistic insight into their function, but the authors do provide a convincing explanation why this was not feasible. The manuscript is well written. I do not have any major concerns regarding the manuscript.

Non-essential suggestions:

Figure 2 D:
It would be helpful to include the description of the bars (black vs white) in the figure (as is done in the rest of the figures).

Page 6 first paragraph, second to last sentence:
One could clarify where the open chromatin difference between Pax5- and Pax5+ progenitors can be found as this is important for the conclusion drawn in the said paragraph.

Referee #2:

Comments for Hill et al. manuscript
Previous studies by the Busslinger lab identified the progenitor B cell-specific Pax-5 transcription-factor-activated intergenic repeat (PAIR) elements that are interspersed within a nearly 800kb portion of the upstream VH locus. PAIR elements were also found to be frequently closely associated with a related set of VH8-family gene segments. The finding that PAX5 inactivation blocked locus contraction and rearrangement of upstream VHs suggested the possibility that PAX5 regulation of PAIR elements might contribute to large scale VH locus contraction and potentially to distal VH rearrangement. However, recent studies from the Busslinger lab demonstrated that PAX5 mediates locus contraction by down-regulating expression of the WAPL cohesin complex unloading factor which, thereby, facilitates loop extrusion through the VH locus (Hill et al., Nature 2020). Parallel studies from another group further showed that WAPL downregulation allows the RAG V(D)J recombination-initiating enzyme from its location in a DJH-based recombination center (RC) at the downstream end of the VH locus to scan through the several Mb long VH locus to locate VH segments for cleavage and joining to DJH to generate VHDJH exons (Dai et al., BioRxiv, 2020; Dai et al, Nat. 2021). In the context of these latter studies, the robust sense and anti-sense transcription and close association with CTCF-binding elements were noted to correlate with relatively robust association of the PAIR4-VH8-7 unit with the RAG-bound RC during loop extrusion in Wapl-downregulated progenitor-B cells. In this regard, earlier studies had clearly implicated transcription in promoting accessibility of genomic target sequences in chromatin to RAG during scanning by allowing their prolonged interaction with the V(D)J recombination center. However, a direct demonstration of the ability of PAIR elements to enhance recombination of associated VH and more importantly, whether they could activate rearrangement of other VHs at a distance remained a very important question.
In the current study, the Busslinger group presents a direct and detailed analysis of the potential function of the PAIR4 element and its associated VH8-7 gene segment with respect to ability to regulate recombination of other VHs over a long distance. PAIR4 and its associated VH8-7 pseudogene (VH8-7) was chosen due to PAIR4 being one of the most highly transcribed and active PAIR elements. Mapping of open chromatin localized a putative regulatory element just downstream of VH8-7, which the authors denote as the VH8-7-associated enhancer (V8.7E); similar open chromatin features were identified just downstream of other VH8 family members. Due to the high conservation of the PAIR sequences, they could potentially have over-lapping, compensatory long range functions that may mask functions of a single mutated element. Therefore, as the first approach to definitively identify a PAIR specific function the authors generated a nearly 900kb VH locus deletion that eliminated all 14 PAIR elements and their interspersed VHs and other sequences from the mouse genome. Then potential functions of the PAIR4-VH8-7 complex were tested by adding different modified versions of these elements back to the point of deletion. Studies of the initial 890-kb deletion demonstrated that it resulted in greatly reduced recombination of all 6 VHs that lie about 100-kb upstream of the deletion point and the first 6 VHs that lie downstream of deletion point, after which rearrangement is normal for the many VHs that lie further downstream (of which most are not highly transcribed). Strikingly, the insertion of PAIR4-VH8.7-E unit strongly activated recombination of the VHs upstream of deletion point in pro-B cells. Reconstitution with just the PAIR4 led to a more modest activation of upstream VH recombination, suggesting that PAIR4 and V8.7E may achieve full function together. Mutation of the Pax5-binding site in the inserted PAIR4 resulted in decreased expression of the PAIR4-derived transcripts and reduced activation of upstream VH rearrangement. Finally, inactivation of the PAIR4-associated CTCF-binding site led to sustained PAIR4 expression in pre-B and immature B cells as well as in T cells. Overall, the authors conclude that PAIR4-V8.7E module can function to enhance V(D)J recombination of distal VHs, at least in a VH locus containing an 890kb deletion of all endogenous PAIR elements. This finding led to their further conclusion that this module contributes similarly in the normal, endogenous VH locus to enhance VH rearrangement and that it further diversifies the BCR repertoire in addition to the diversification provided by chromatin loop extrusion.
This study represents an impressive amount of work. Moreover, it provides the first clear evidence for a function of a PAIR element, which is functioning to enhance transcription and VH to DJH recombination of distant VHs. While these initial studies were necessarily done within a highly modified IgH locus, the authors' rationale for doing the studies in this manner was well-justified considering the potential redundancy of the function of the 14 PAIR elements spread across the deleted region. In this context, the add back experiments shown in this study by Hill et al. provide compelling evidence that the PAIR4 element and associated sequences can, in some way, function as recombinational enhancers. While there is much to be done in terms of elucidating how this PAIR works, potentially together with others, across the normal VH locus, this very interesting and comprehensive study represents an important first step which should be of significant interest in guiding future studies of the field and beyond. I do believe that, before publication, this paper could be further polished to better explain the rationale for the study, better describe potential interpretational caveats that accompany all genomic studies of this magnitude, and to better present the interesting findings in the context of the full current literature.

Comments:
1. The authors showed that the distal enhancers promote local VH recombination and speculated about the molecular mechanisms in light of open chromatin and RAG accessibility. Since the authors acknowledge and indeed made critical contributions to elucidating the fundamental role of loop extrusion in Igh V(D)J recombination, it seems very reasonable that they should better discuss the potential mechanisms by which the PAIR4 and V8.7E unit may function in the context of loop extrusion. They may wish to cite some of the robust loop extrusion-RAG-scanning literature that is likely very relevant to their findings. For example, Zhang et al. Nature, 2019 provide definitive evidence that transcription targets RC-bound RAG activity to transcribed chromosomal targets (cryptic RSSs) that are presented to it during scanning due to transcription impeding their

extrusion past the RC center and, thereby, allowing extended interactions with it. This mechanism for transcription is similar in operation to the way earlier studies demonstrated that proximal VH-associated CBEs increase targeting of associated VHs during extrusion past the RC (Jain et al., Cell, 2018). These findings have the potential to be very relevant to the activity of the Pair4 element both locally and also by potentially by activating VH transcription at a distance. In the latter context, Ba et al. Nature 2020 and Dai et al. Nature 2021 papers both demonstrated correlations between nascent transcription and local CBEs in the distal VH region with respect to the utilization of nearby VHs. I feel that a more thorough discussion on the role of transcription in generating loop extrusion-mediated RAG scanning impediments in light of these papers would greatly benefit this manuscript.
2. It is notable that all effects observed with the different insertions only affected the VHs with reduced V(D)J recombination upstream of the deletion point/insertion site and not those with reduced V(D)J recombination downstream of the site. The authors do not call attention to this interesting point that deserves significant discussion. Indeed, this directionality of the PAIR4 and V8.7E activity may be consistent with their proposed role for loop extrusion in mediating interactions of this element with upstream VHs to promote their transcription/V(D)J recombination activity. Also, they may wish to further emphasize the possibility that they may have removed other elements or modes of V(D)J recombinational enhancement in the context of their nearly 1Mb VH locus deletion. In this regard, it is perhaps interesting to note that the downstream VHs that continue to rearrange after the deletion are largely associated with a CBE-based mode of recombinational accessibility (with a few exceptions) (Dai et al, Nature, 2021; main, extended data, and Supplementary Figures 2 and 3).
3. I suggest that the authors tone down or eliminate their paragraph that states that 3C-based methods cannot be used to verify specific interactions of elements within the VH locus. We suggest that the authors consider the very specific interactions found between the IgH recombination center and specific sites across the VH locus in normal pro-B cells or in WAPl-depleted v-Abl cells. Indeed, many of these highly robust interaction peaks of the VH locus interaction with the RC were focused directly on PAIR elements including PAIR4 and V8.7E (Dai et al. Nature 2021). Notably, these robust peaks, and where present associated transcription levels and patterns, were retained even when the entire VH locus was inverted. The data is shown in a main figure of that paper, in an extended data figure, and in great detail and high resolution across the entire locus in Supplementary Data 2 and 3. Likewise, we would encourage the authors to revise paragraph based on extended data Figure 7 in Dai et al, BioRxiv, 2020. That figure shows the highly specific interaction patterns of VH8.7 with other specific peaks within the VH locus, several of which correspond to VHs associated with PAIR elements.
4. The authors showed that mutating the PAX5 binding site in PAIR4 only modestly decreased PAIR4 transcription, which is different from the result in Pax5-/- mice where all PAIR transcription seemed to be abolished (Ebert et al. 2011). This finding suggests the possibility that the latter result could in part be due to developmental effects, in which B cell development in Pax5-/- mice is arrested at an earlier stage than pro-B stage (Hill et al. Nature 2020) in which PAIR transcription may not be activated yet. It may be helpful if the authors discuss this further.
5. The authors should acknowledge that their findings were found in a locus in which nearly a Mb of sequence containing many potential control elements was deleted. This acknowledgement does not detract from their positive, very important, and publishable findings of the recombinational enhancing activity they demonstrate for PAIR4 and V8.7E, but it would put it into a better context for the uninitiated reader.
Minor comments:
1. In the abstract and other summaries, the authors should more clearly define the scope of activation following enhancer insertion they detail in their text. Given that for the distal 6 VHs, the majority of rearrangement occurred within the first 3 VHs (1-80, 1-81, 1-82), the effect may be more local than the authors described in their overall summary statements.
2. The authors claimed that "CTCF ensures the pro-B cell-specific activity of PAIR4 by suppressing the activity of this element at later B cell developmental stages and during T lymphopoiesis". However, CTCF also suppresses PAIR transcription in pro-B cells (Figure 6A), just like it does in other cell stages. It seems that this repressing activity of CTCF does not have cell type specificity.
3. In Fig.3B, it seems that the three mouse lines were not analyzed at the same time, and there are noticeable differences between the two experiments (compare top right to bottom left). It is also not straightforward how data in panel B fit into the statistical analysis in panel C. The authors should clarify these points.
4. In their summary statements the authors indicate that PAIR4-V8.7E module contributes to VH gene recombination and VH repertoire diversification of the BCR repertoire in addition to loop extrusion. It seems quite probable to this reviewer that this module may contribute in the context of a loop extrusion mechanism and not in addition to it.

Referee #3:

In this manuscript Hill and colleagues address the function of PAIR elements in the Igh locus. Although these intriguing elements were initially identified and characterized over 10 years ago, we know very little about their functional significance, in large part due to technical challenges relating to the fact that there are multiple PAIRs distributed across a large segment of the Igh locus. The authors approach this problem by removing all PAIR elements (and many V segments) from the Igh locus by creating an 890kb deletion, and then reintroducing wild-type or mutant copies of PAIR4. They analyze as well the significance of a newly described regulatory element tightly associated with PAIR4-associated VH8.7, as well as other Vh8 family members. The 890kb deletion was associated with reduced usage of VH segments flanking the deletion. This deficit in usage was reversed by reintroduction of PAIR4-VH8.7E, which was, in turn, more effective than PAIR4 alone, indicating contributions from both elements. Contribution by the downstream enhancer was confirmed by separate analysis of an analogous element associated

with VH8.8. Mechanistic experiments addressed the significance of PAIR4 orientation and transcription, but with somewhat ambiguous results: both contribute to PAIR4 activity, but neither is required. Pax5 binding to PAIR4 was shown to promote PAIR4 transcription and modestly enhance distant VH rearrangements. Interestingly, CTCF binding to PAIR4 strongly suppressed PAIR 4 transcription and limited it to pro-B cells, but it enhanced recombination locally.

This is an outstanding manuscript that makes use of a large set of complex genetic manipulations to fill a significant gap in knowledge about Igh locus regulation. I believe that it will be of broad interest to the readership of the EMBO J. I have only minor comments and concerns.

1. Do the authors know whether the insertion or removal of the promoterless Bsd gene influences any of the phenotypes they observe? Some comment on this seems warranted.
2. Fig 2D suggests a rather sharp boundary between VH genes whose rearrangement is suppressed by the 890 deletion and more downstream VH genes that are unaffected. The authors may want to address whether there is such a boundary and if so, what features are associated with it.
3. If the increase in VDJ/DJ for P4V does not reach statistical significance (EV3a) the authors should state this clearly.
4. The statement on p11 that both orientations of P4 could promote VH recombination seems to oversimplify the results: P4 increased frequency by 0.14 over 890 deletion, but P4inv by only 0.04. That is a 3.5-fold difference in activity which certainly suggests an orientation bias. Similarly, the characterization of the VDJ-seq data immediately following states that the forward orientation is "slightly better" than inverted, when in fact the increase in rearrangement over 890 was twice as high for forward (2.7-1.0=1.7) as compared to reverse (1.9-1.0=0.9). The authors seem to gloss over an orientation bias that is apparent in two different assays.
5. The authors explain residual apparent Pax5 binding to the mutated P4 element as a consequence of looping from V8.7E. This interaction is stated as fact. Was this interaction experimentally determined or is this speculation? Please clarify.
6. It is suggested that the 5' region of the Igh locus may provide a special environment that restricts the activity of Eu and possibly PAIR4-V8.7E to the pro-B cell stage. But the authors have already shown that mutation of the PAIR4 CTCF binding site in PAIR4-V8.7E eliminates both developmental stage- and lineage-specificity of PAIR4 transcription in this environment. How can these observations be reconciled?
7. In the Discussion the CTCF site of PAIR4 is referred to as an insulator. But none of the data presented suggest anything more than that CTCF is functioning to suppress transcription. The mechanism could be quite different from insulation per se.

Point-by-point to the reviews of the manuscript EMBOJ-2022-112741R-Q

Enclosed, we submit our point-by-point reply (in blue) to the reviewer's comments (in black). We have performed several new experiments and improved the discussion by explaining the results of our reconstitution experiments in the context of recently described new mechanism of loop extrusion. These changes have significantly improved the main message of the manuscript in our opinion.

Referee #1:

The manuscript "Enhancers of the PAIR4 regulatory module promote distal VH gene recombination at the Igh locus" by Hill et al. provides a detailed analysis on the function of PAIR4 element in regulation of VH sequence usage in VDJ recombination during early B cell development. The authors show that PAIR4 element, particularly together with a newly identified enhancer element in the immediate vicinity of a V8-7 gene segment, can rescue the deletion of a larger region within the VH region lacking all 14 PAIR elements in promoting distal VH usage. They also show that the use of distal VH gene segments can be rescued with other enhancers (V8-8 with its respective enhancer and with the well characterized Emu enhancer). The findings are novel, build on their previous findings on the PAIR element. The findings presented in the manuscript are important in understanding the mechanism and fine tuning of the antibody repertoire formation. The antibody repertoire formation has some relevance for antibody response to vaccination and is associated with human disease.

The work is carried out with great scrutiny, uses ingenious mouse models, and the conclusions drawn from the data are appropriate. It would have been curious to learn how the investigated elements affect the chromatin structure as this would have given greater mechanistic insight into their function, but the authors do provide a convincing explanation why this was not feasible. The manuscript is well written. I do not have any major concerns regarding the manuscript.

We thank the reviewer for the very positive evaluation of our manuscript.

Non-essential suggestions:

Figure 2 D:
It would be helpful to include the description of the bars (black vs white) in the figure (as is done in the rest of the figures).

To improve the description, we have now positioned the black and white bars with the corresponding genotypes between the upper enlargement and the lower experimental data in Figure 2D.

Page 6 first paragraph, second to last sentence:
One could clarify where the open chromatin difference between Pax5- and Pax5+ progenitors can be found as this is important for the conclusion drawn in the said paragraph.

To make the open chromatin peaks (determined by ATAC-seq) better visible in Figure EV1A,B, we have removed the red dotted line in the vicinity of ATAC-seq peaks.

Referee #2:

Comments for Hill et al. manuscript

Previous studies by the Busslinger lab identified the progenitor B cell-specific Pax-5 transcription-factor-activated intergenic repeat (PAIR) elements that are interspersed within a nearly 800kb portion of the upstream VH locus. PAIR elements were also found to be frequently closely associated with a related set of VH8-family gene segments. The finding that PAX5 inactivation blocked locus contraction and rearrangement of upstream VHs suggested the possibility that PAX5 regulation of PAIR elements might contribute to large scale VH locus contraction and potentially to distal VH rearrangement. However, recent studies from the Busslinger lab demonstrated that PAX5 mediates locus contraction by down-regulating expression of the WAPL cohesin complex unloading factor which, thereby, facilitates loop extrusion through the VH locus (Hill et al., Nature 2020). Parallel studies from another group further showed that WAPL downregulation allows the RAG V(D)J recombination-initiating enzyme from its location in a DJH-based recombination center (RC) at the downstream end of the VH locus to scan through the several Mb long VH locus to locate VH segments for cleavage and joining to DJH to generate VHDJH exons (Dai et al., BioRxiv, 2020; Dai et al, Nat. 2021). In the context of these latter studies, the robust sense and anti-sense transcription and close association with CTCF-binding elements were noted to correlate with relatively robust association of the PAIR4-VH8-7 unit with the RAG-bound RC during loop extrusion in Wapl-downregulated progenitor-B cells. In this regard, earlier studies had clearly implicated transcription in promoting accessibility of genomic target sequences in chromatin to RAG during scanning by allowing their prolonged interaction with the V(D)J recombination center. However, a direct demonstration of the ability of PAIR elements to enhance recombination of associated VH and more importantly, whether they could activate rearrangement of other VHs at a distance remained a very important question.

In the current study, the Busslinger group presents a direct and detailed analysis of the potential function of the PAIR4 element and its associated VH8-7 gene segment with respect to ability to regulate recombination of other VHs over a long distance. PAIR4 and its associated VH8-7 pseudogene (VH8-7) was chosen due to PAIR4 being one of the most highly transcribed and active PAIR elements. Mapping of open chromatin localized a putative regulatory element just downstream of VH8-7, which the authors denote as the VH8-7-associated enhancer (V8.7E); similar open chromatin features were identified just downstream of other VH8 family members. Due to the high conservation of the PAIR sequences, they could potentially have over-lapping, compensatory long-range functions that may mask functions of a single mutated element. Therefore, as the first approach to definitively identify a PAIR specific function the authors generated a nearly 900kb VH locus deletion that eliminated all 14 PAIR elements and their interspersed VHs and other sequences from the mouse genome. Then potential functions of the PAIR4-VH8-7 complex were tested by adding different modified versions of these elements back to the point of deletion. Studies of the initial 890-kb deletion demonstrated that it resulted in greatly reduced recombination of all 6 VHs that lie about 100-kb upstream of the deletion point and the first 6 VHs that lie downstream of deletion point, after which rearrangement is normal for the many VHs that lie further downstream (of which most are not highly transcribed). Strikingly, the insertion of PAIR4-VH8.7-E unit strongly activated recombination of the VHs upstream of deletion point in pro-B cells. Reconstitution with just the PAIR4 led to a more modest activation of upstream VH recombination, suggesting that PAIR4 and V8.7E may achieve full function together. Mutation of the Pax5-binding site in the inserted PAIR4 resulted in decreased expression of the PAIR4-derived transcripts and reduced activation of upstream VH rearrangement. Finally, inactivation of the PAIR4-associated CTCF-binding site led to sustained PAIR4 expression in pre-B and

immature B cells as well as in T cells. Overall, the authors conclude that PAIR4-V8.7E module can function to enhance V(D)J recombination of distal VHs, at least in a VH locus containing an 890kb deletion of all endogenous PAIR elements. This finding led to their further conclusion that this module contributes similarly in the normal, endogenous VH locus to enhance VH rearrangement and that it further diversifies the BCR repertoire in addition to the diversification provided by chromatin loop extrusion.

This study represents an impressive amount of work. Moreover, it provides the first clear evidence for a function of a PAIR element, which is functioning to enhance transcription and VH to DJH recombination of distant VHs. While these initial studies were necessarily done within a highly modified IgH locus, the authors' rationale for doing the studies in this manner was well-justified considering the potential redundancy of the function of the 14 PAIR elements spread across the deleted region. In this context, the add back experiments shown in this study by Hill et al. provide compelling evidence that the PAIR4 element and associated sequences can, in some way, function as recombinational enhancers. While there is much to be done in terms of elucidating how this PAIR works, potentially together with others, across the normal VH locus, this very interesting and comprehensive study represents an important first step which should be of significant interest in guiding future studies of the field and beyond. I do believe that, before publication, this paper could be further polished to better explain the rationale for the study, better describe potential interpretational caveats that accompany all genomic studies of this magnitude, and to better present the interesting findings in the context of the full current literature.

We thank this reviewer for the very positive evaluation of our manuscript.

Comments:

1. The authors showed that the distal enhancers promote local VH recombination and speculated about the molecular mechanisms in light of open chromatin and RAG accessibility. Since the authors acknowledge and indeed made critical contributions to elucidating the fundamental role of loop extrusion in Igh V(D)J recombination, it seems very reasonable that they should better discuss the potential mechanisms by which the PAIR4 and V8.7E unit may function in the context of loop extrusion. They may wish to cite some of the robust loop extrusion-RAG-scanning literature that is likely very relevant to their findings. For example, Zhang et al. Nature, 2019 provide definitive evidence that transcription targets RC-bound RAG activity to transcribed chromosomal targets (cryptic RSSs) that are presented to it during scanning due to transcription impeding their extrusion past the RC center and, thereby, allowing extended interactions with it. This mechanism for transcription is similar in operation to the way earlier studies demonstrated that proximal VH-associated CBEs increase targeting of associated VHs during extrusion past the RC (Jain et al., Cell, 2018). These findings have the potential to be very relevant to the activity of the Pair4 element both locally and also by potentially by activating VH transcription at a distance. In the latter context, Ba et al. Nature 2020 and Dai et al. Nature 2021 papers both demonstrated correlations between nascent transcription and local CBEs in the distal VH region with respect to the utilization of nearby VHs. I feel that a more thorough discussion on the role of transcription in generating loop extrusion-mediated RAG scanning impediments in light of these papers would greatly benefit this manuscript.

We thank the review for pointing out aspects of loop extrusion that are relevant for the interpretation of our results. We now mention the important result of Zhang et al. (2019) that describes that an active promoter (at Sγ2b) can promote prolonged interaction with the recombination center in the absence of CTCF-binding sites. We cite this paper in the context of the activation and increased recombination of distal V_H genes by the PAIR-V8.7E module on page 22 (top). We cite Jain et al. (2018) for the role of CTCF in promoting accessibility and interaction

of the most proximal V_H genes with the recombination center on page 25 (bottom part). We also mention the paper of Dai et al. (2022) on page 21 (middle) for the identification of robust and specific interactions between the recombination center in the *Igh* 3' region and the transcriptionally active PAIR elements and V8 genes in the distal V_H gene region, including PAIR4, V8-7 and V8-8. As the discussion of all the different aspects of *Igh* loop extrusion is beyond the scope of our research article, we now cite the elegant review of Zhang et al. (2022) describing multiple aspects of *Igh* loop extrusion. We furthermore discuss the results of our paper in the context of extended *Igh* loop extrusion also in response to point 2 of this reviewer by explaining in a new Figure EV7B,C how the mechanism of loop extrusion can explain some of the recombination phenotypes described in our study.

2. It is notable that all effects observed with the different insertions only affected the VHs with reduced V(D)J recombination upstream of the deletion point/insertion site and not those with reduced V(D)J recombination downstream of the site. The authors do not call attention to this interesting point that deserves significant discussion. Indeed, this directionality of the PAIR4 and V8.7E activity may be consistent with their proposed role for loop extrusion in mediating interactions of this element with upstream VHs to promote their transcription/V(D)J recombination activity. Also, they may wish to further emphasize the possibility that they may have removed other elements or modes of V(D)J recombinational enhancement in the context of their nearly 1Mb VH locus deletion. In this regard, it is perhaps interesting to note that the downstream VHs that continue to rearrange after the deletion are largely associated with a CBE-based mode of recombinational accessibility (with a few exceptions) (Dai et al, Nature, 2021; main, extended data, and Supplementary Figures 2 and 3).

We thank the reviewer for pointing out that we did not explain the two kinds of directionalities that we observed upon reconstitution of the 890-kb deletion with the PAIR4-V8.7E module: 1) the increased recombination of V_H genes, located upstream of the insertion, compared to the downstream located V_H genes (Figure 3D,F and EV3D) and 2) the decrease of upstream V_H gene recombination upon inversion of the PAIR4 element (Figure 3F,G). Both effects can be explained by the mechanism of prolonged loop extrusion that operates at the *Igh* locus in pro-B cells, as suggested by the reviewer. As suggested by the reviewer, we now emphasize at the beginning of the discussion section that the 890-kb deletion eliminates many potential regulatory elements in addition to the PAIR sequences (page 20, top).

1) First, we have quantified the effect of the inserted PAIR4-V8.7E and PAIR4 elements on the recombination efficiency of the next six V_H1 genes that are located downstream of the insertion in *Igh*$^{P4V/P4V}$ and *Igh*$^{P4/P4}$ pro-B cells. As shown by the new data in Figure EV3D, the recombination of the downstream V_H1 genes was activated 2-3-fold less efficiently compared to the recombination of the upstream V_H1 genes in *Igh*$^{P4V/P4V}$ and *Igh*$^{P4/P4}$ pro-B cells. To explain this recombination difference, it is important to mention that the loop extrusion process generates an almost contiguous interaction zone, where all the different sequences within the V_H gene region appear to interact with each other, as shown by our recent publication (Hill et al., 2023, Nat. Commun. 14, 2316). The generation of this contiguous interaction zone requires that all CTCF-binding sites are present in forward orientation in the V_H gene cluster (Hill et al., Nature 584, 142-147). Loop extrusion likely initiates at random positions in the V_H gene cluster and initially proceeds in a symmetrical manner, until the cohesin ring interacts with a CTCF protein bound to the next upstream forward-oriented CTCF-binding site, which leads to stabilized binding of cohesin at this site (Li et al., Nature 578, 472-476) (Figure EV7B). Thereafter, asymmetrical loop extrusion reels the DNA of the downstream *Igh* region into the loop (Figure EV7B), until it is halted

by a CTCF protein bound to a reverse CTCF-binding site in convergent orientation at the IGCR1 or 3'CBE elements. This model predicts that only the V_H1 genes located upstream of the PAIR4 insertion can efficiently interact through loop extrusion with the inserted PAIR4 element, which results in strong activation of V_H gene recombination, as explained in Figure EV7B. In contrast, the downstream V_H1 genes are unable to interact in this manner with the inserted PAIR4 element, which leads to lower V_H gene recombination. We mention this explanation for the functional directionality of the PAIR4 element in the control of V_H gene recombination on page 22.

2) The inversion of PAIR4 also inverts the orientation of the CTCF-binding site of PAIR4 in *Igh*$^{P4inv/P4inv}$ pro-B cells relative to *Igh*$^{P4/P4}$ pro-B cells. As a consequence, the reverse-oriented CTCF-binding site of PAIR4 is now in convergent orientation relative to the forward CTCF-binding sites in the upstream region and can thus form new stabilized loops (Figure EV7C) that prevent prolonged loop extrusion beyond the inserted PAIR4 element and can thus interfere with the interaction of the upstream V_H genes with the RAG$^+$ recombination center in the *Igh* 3' region, as shown before for the inversion of the 890-kb V_H gene region and upon insertion of an array of 20 reverse CTCF-binding sites (Hill et al., Nature 584, 142-147). This could explain why the distal V_H gene recombination is reduced in *Igh*$^{4inv/P4inv}$ pro-B cells relative to *Igh*$^{P4/P4}$ pro-B cells (Fig. 3F,G), as shown in Figure EV7C. We mention this explanation in the discussion on page 22.

3. I suggest that the authors tone down or eliminate their paragraph that states that 3C-based methods cannot be used to verify specific interactions of elements within the VH locus. We suggest that the authors consider the very specific interactions found between the IgH recombination center and specific sites across the VH locus in normal pro-B cells or in WAPI-depleted v-Abl cells. Indeed, many of these highly robust interaction peaks of the VH locus interaction with the RC were focused directly on PAIR elements including PAIR4 and V8.7E (Dai et al. Nature 2021). Notably, these robust peaks, and where present associated transcription levels and patterns, were retained even when the entire VH locus was inverted. The data is shown in a main figure of that paper, in an extended data figure, and in great detail and high resolution across the entire locus in Supplementary Data 2 and 3. Likewise, we would encourage the authors to revise paragraph based on extended data Figure 7 in Dai et al, BioRxiv, 2020. That figure shows the highly specific interaction patterns of VH8.7 with other specific peaks within the VH locus, several of which correspond to VHs associated with PAIR elements.

In response to the reviewer's comment, we have investigated the specificity of interaction by 3C-qPCR analysis, which demonstrated that the DNA sequences at a viewpoint located immediately downstream the V8.7E enhancer interacted with the distal V_H genes in *Igh*$^{P4V/P4V}$ pro-B cells at an increased frequency compared with control T cells (new Figure EV4B). Surprisingly however, the interactions from this viewpoint to the distal V_H genes were also formed with similar frequency in *Igh*$^{\Delta 890/\Delta 890}$ pro-B cells. In contrast to the observed equal interaction efficiency, active histone marks were specifically induced by insertion of the PAIR4-V8.7E module in *Igh*$^{P4V/P4V}$ pro-B cells compared with *Igh*$^{\Delta 890/\Delta 890}$ pro-B cells. Together, these data indicate that insertion of the PAIR4-V8.7E module strongly activated recombination of the six most distal V_H genes over a distance of up to 112 kb by inducing active chromatin without promoting the long-range interactions to these V_H genes. We describe the new 3C-qPCR data in the result and discussion sections on pages 11 and 21 (lower part), respectively. In the meantime, we published that the loop extrusion process generates a largely contiguous interaction zone, where all the different sequences within the V_H gene region appear to interact with each other (Hill et al., 2023, Nat. Commun. 14, 2316). Hence, the interaction between the PAIR4-V8.7E module and the upstream distal V_H gene is likely brought about by the prolonged loop extrusion mechanism shown

in Figure EV7B and described on page 22 of the manuscript, which is also mentioned above in response to point 2 of this reviewer. Our interaction data are seemingly at odds with data published by the group of Fred Alt. They identified discrete interaction peaks in the V_H gene cluster with the 3C-HTGTS method by using an RC/Eμ or V_H8-7P viewpoint, which identified specific interactions between the RAG^+ RC and transcriptionally active PAIR elements and V8 genes in the distal V_H gene region, including PAIR4, V8-7and V8-8 (Dai et al., Nature 590, 338-343), which we now mention in the discussion section on page 21 (middle). One explanation for this discrepancy could be that two different interaction modalities exist at the *Igh* locus. Prolonged loop extrusion is responsible for most interactions within the V_H gene cluster by generating of contiguous interaction zone, while, on top of this general interaction pattern, more specific interactions exist particularly between the RAG^+ recombination center (RC) and distinct sequences in the V_H gene cluster.

4. The authors showed that mutating the PAX5 binding site in PAIR4 only modestly decreased PAIR4 transcription, which is different from the result in Pax5-/- mice where all PAIR transcription seemed to be abolished (Ebert et al. 2011). This finding suggests the possibility that the latter result could in part be due to developmental effects, in which B cell development in Pax5-/- mice is arrested at an earlier stage than pro-B stage (Hill et al. Nature 2020) in which PAIR transcription may not be activated yet. It may be helpful if the authors discuss this further.

We thank the reviewer for pointing this out. We now mention this fact in the discussion section (page 23, top) with reference to our data obtained with $Pax5^-$ and $Pax5^+$ BLPs.

5. The authors should acknowledge that their findings were found in a locus in which nearly a Mb of sequence containing many potential control elements was deleted. This acknowledgement does not detract from their positive, very important, and publishable findings of the recombinational enhancing activity they demonstrate for PAIR4 and V8.7E, but it would put it into a better context for the uninitiated reader.

We added a corresponding statement in the first paragraph of the discussion on page 20 (top).

Minor comments:

1. In the abstract and other summaries, the authors should more clearly define the scope of activation following enhancer insertion they detail in their text. Given that for the distal 6 VHs, the majority of rearrangement occurred within the first 3 VHs (1-80, 1-81, 1-82), the effect may be more local than the authors described in their overall summary statements.

Although the first proximal V_H genes (1-80, 1-81, 1-82) recombine most efficiently, we would like to emphasize that the recombination of all six distal V_H genes is similarly induced by the PAIR4-V8.7E module in $Igh^{P4V/P4V}$ pro-B cells compared with $Igh^{\Delta 890/\Delta 890}$ pro-B cells, although starting from vastly different recombination frequencies of these V_H genes in $Igh^{+/+}$ pro-B cells (Figures 2D and EV3C). We thus argue that the activity of the PAIR4-V8.7E module reaches over a region of more than 100 kb and affects all recombining V_H genes upstream of the insertion.

2. The authors claimed that "CTCF ensures the pro-B cell-specific activity of PAIR4 by suppressing the activity of this element at later B cell developmental stages and during T lymphopoiesis". However, CTCF also suppresses PAIR transcription in pro-B cells (Figure 6A), just like it does in other cell stages. It seems that this repressing activity of CTCF does not have cell type specificity.

The mutation of the CTCF-binding site in PAIR4 does not increase the activity of PAIR4 in uncommitted BLP cells of *Igh*$^{P4\Delta CtcfGV/P4\Delta CtcfGV}$ mice (Figure EV5H) in contrast to the increased activity observed in B and T cells of these mice (Figure 6A-D), although CTCF binding is observed at PAIR4 in uncommitted *Pax5*$^{-/-}$ progenitors (Ebert et al., Immunity 34, 175-187). While CTCF is known to be a ubiquitous transcription factor, our data suggest that the effect of CTCF loss at PAIR4 also depends on a cell-type-specific regulatory context. It is interesting to note that the PAIR4 element is not present in open chromatin but is bound by CTCF both in DP thymocyte and Pax5^{-} BLPs, and yet the loss of CTCF binding at PAIR4 in these two different cell types has a different outcome.

3. In Fig.3B, it seems that the three mouse lines were not analyzed at the same time, and there are noticeable differences between the two experiments (compare top right to bottom left). It is also not straightforward how data in panel B fit into the statistical analysis in panel C. The authors should clarify these points.

The data of Figure 3B were indeed generated in two separate experiments. In Figure 3B, we measure the actual ratios. Importantly, the ratios determined for the *Igh*$^{\Delta 890(B6)/+(129)}$ genotype (open circles) were consistent between the separate experiments and are thus very similar in both panels of Figure 3B, as also shown in Figure 1 (for reviewer). The reviewer may have overlooked that the y-axes are different between the two panels. For better clarity, we have exchanged the order of the data in the lower panel so that *Igh*$^{\Delta 890(B6)/+(129)}$ genotype is now also on the right side. In Figure 2C, we normalized the ratios of all the different experiments relative to the ratio obtained with the control *Igh*$^{+(B6)/+(129)}$ genotype (black bar) that was set to 1, as described in the figure legend.

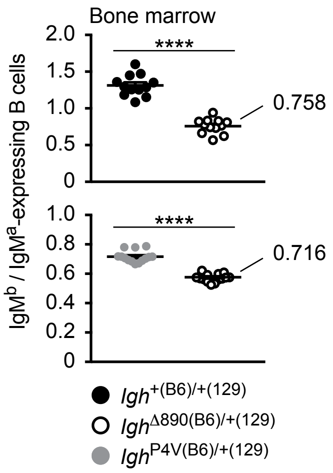

Figure 1 for reviewer.

New arrangement of the data of Figure 3B (right) to indicate that a similar ratio of IgM^{b} / IgM^{a} B cells was determined for the control *Igh*$^{+(B6)/+(129)}$ genotype (open circles) in both experiments.

4. In their summary statements the authors indicate that PAIR4-V8.7E module contributes to VH gene recombination and VH repertoire diversification of the BCR repertoire in addition to loop extrusion. It seems quite probable to this reviewer that this module may contribute in the context of a loop extrusion mechanism and not in addition to it.

We thank the reviewer for pointing this out and now mention in the abstract and throughout the manuscript that the PAIR4-V8.7E-dependent V_H repertoire diversification occurs in the context of loop extrusion, as suggested by the reviewer.

Referee #3:

In this manuscript Hill and colleagues address the function of PAIR elements in the Igh locus. Although these intriguing elements were initially identified and characterized over 10 years ago, we know very little about their functional significance, in large part due to technical challenges relating to the fact that there are multiple PAIRs distributed across a large segment of the Igh locus. The authors approach this problem by removing all PAIR elements (and many V segments) from the Igh locus by creating an 890kb deletion, and then reintroducing wild-type or mutant copies of PAIR4. They analyze as well the significance of a newly described regulatory element tightly associated with PAIR4-associated VH8.7, as well as other Vh8 family members. The 890kb deletion was associated with reduced usage of VH segments flanking the deletion. This deficit in usage was reversed by reintroduction of PAIR4-VH8.7E, which was, in turn, more effective than PAIR4 alone, indicating contributions from both elements. Contribution by the downstream enhancer was confirmed by separate analysis of an analogous element associated with VH8.8. Mechanistic experiments addressed the significance of PAIR4 orientation and transcription, but with somewhat ambiguous results: both contribute to PAIR4 activity, but neither is required. Pax5 binding to PAIR4 was shown to promote PAIR4 transcription and modestly enhance distant VH rearrangements. Interestingly, CTCF binding to PAIR4 strongly suppressed PAIR 4 transcription and limited it to pro-B cells, but it enhanced recombination locally.

This is an outstanding manuscript that makes use of a large set of complex genetic manipulations to fill a significant gap in knowledge about Igh locus regulation. I believe that it will be of broad interest to the readership of the EMBO J. I have only minor comments and concerns.

We thank this reviewer for the very positive evaluation of our manuscript.

1. Do the authors know whether the insertion or removal of the promoterless Bsd gene influences any of the phenotypes they observe? Some comment on this seems warranted.

The promoter-less Bsd gene is only present at the breakpoint of the 890-kb deletion in the *Igh*$^{\Delta 890}$ allele (Figure EV2A). In this context, it is unlikely that the bacterial Bsd gene is responsible for the reduced recombination of the distal V_H genes up to a distance of 100 kb on either side of the deletion. Instead, elimination of the many potential regulatory elements located in the 890-kb region (see Figure EV7A) is very likely to cause the reduced V_H gene recombination efficiency observed in *Igh*$^{\Delta 890/\Delta 890}$ pro-B cells. Importantly however, after Floxin-mediated insertion of the different PAIR4 constructs, we removed all bacterial DNA including the Bsd gene by Dre-mediated recombination, which only left an *frt* and *rox* site in the engineered *Igh* locus, as shown in Figure EV2C.

2. Fig 2D suggests a rather sharp boundary between VH genes whose rearrangement is suppressed by the 890 deletion and more downstream VH genes that are unaffected. The authors may want to address whether there is such a boundary and if so, what features are associated with it.

This is an interesting suggestion, which we further analyzed. In Figure 2 (for reviewer), we show a subtraction plot, in which the recombination frequency of each V_H gene in *Igh*$^{\Delta 890/\Delta 890}$ pro-B cells is subtracted from the respective frequency of the same V_H gene in *Igh*$^{+/+}$ pro-B cells. These new data clearly indicated that there is no sharp boundary between the V_H1-27 and V_H1-26 genes as suggested by the reviewer.

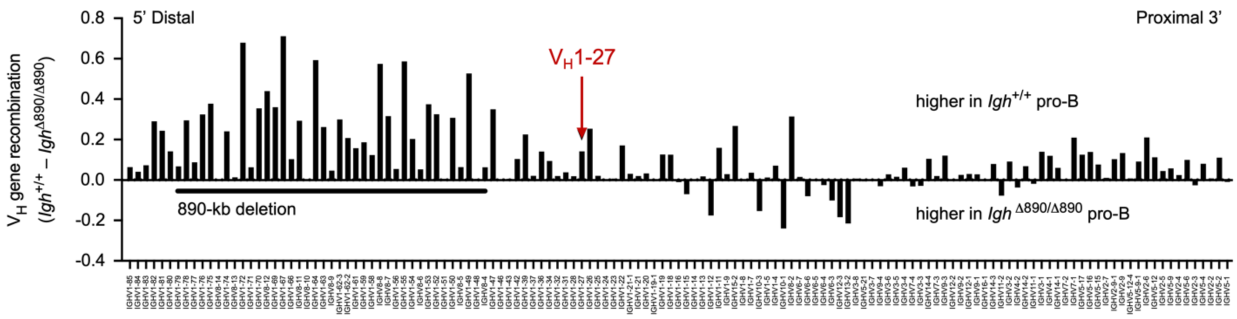

Figure 2 for reviewer. Difference of V_H gene usage across the *Igh* locus, which was determined for each V_H gene by subtracting its mean recombination frequency in $Igh^{\Delta890/\Delta890}$ pro-B cells from that of $Igh^{+/+}$ pro-B cells.

3. If the increase in VDJ/DJ for P4V does not reach statistical significance (EV3a) the authors should state this clearly.

The reviewer presumably refers to Figure EV3B. The high variance of the data obtained with control $Igh^{+/+}$ pro-B cells (many more data points) is likely responsible for the fact that only the comparison between $Igh^{+/+}$ and $Igh^{\Delta890/\Delta890}$ pro-B cells is statistically significant. We have added a sentence in the legend of Figure EV3B to mention that all other comparisons did not reach statistical significance.

4. The statement on p11 that both orientations of P4 could promote VH recombination seems to oversimplify the results: P4 increased frequency by 0.14 over 890 deletion, but P4inv by only 0.04. That is a 3.5-fold difference in activity which certainly suggests an orientation bias. Similarly, the characterization of the VDJ-seq data immediately following states that the forward orientation is "slightly better" than inverted, when in fact the increase in rearrangement over 890 was twice as high for forward (2.7-1.0=1.7) as compared to reverse (1.9-1.0=0.9). The authors seem to gloss over an orientation bias that is apparent in two different assays.

We strengthened the statement about the orientation bias of PAIR4 function by changing “slightly better” to “better” on page 12 (middle). See detailed reply to point 2.2 of reviewer #2, who raised the same issue.

5. The authors explain residual apparent Pax5 binding to the mutated P4 element as a consequence of looping from V8.7E. This interaction is stated as fact. Was this interaction experimentally determined or is this speculation? Please clarify.

As the PAIR4 and V8.7E elements are separated by a short distance of 4 kb, it is not possible to verify an interaction of these two elements by 3C-qPCR. To further investigate Pax5 binding to the mutant Pax5 recognition sequence, we now performed an electrophoretic mobility shift assay with a B cell nuclear extract. These new data (shown in Figure EV5F) unequivocally demonstrate that Pax5 is unable to bind the mutant site. We therefore assume that the residual Pax5 binding seen by ChIP-qPCR is caused by crosslinking of Pax5 (bound to V8.7E) to PAIR4 through a looping interaction. We describe these new data on page 15.

6. It is suggested that the 5' region of the Igh locus may provide a special environment that restricts the activity of Eu and possibly PAIR4-V8.7E to the pro-B cell stage. But the authors have already

shown that mutation of the PAIR4 CTCF binding site in PAIR4-V8.7E eliminates both developmental stage- and lineage-specificity of PAIR4 transcription in this environment. How can these observations be reconciled?

The reviewer raises an interesting question, for which we have no satisfying molecular explanation at present. At face value, it appears that the CTCF-mediated repression of PAIR4 is dominant over the general repression mechanism that may operate in the distal V_H gene region to repress the inserted Eμ enhancer at its distal location in the *Igh* locus. However, it is important to mention that the CTCF-binding site in PAIR4 likely acts very locally to repress only the lncRNA transcription from PAIR4 in B and T cells. In contrast, the postulated general repression mechanism may affect a larger region of the distal V_H gene region and, in this context, may also repress the distally inserted Eμ enhancer.

7. In the Discussion the CTCF site of PAIR4 is referred to as an insulator. But none of the data presented suggest anything more than that CTCF is functioning to suppress transcription. The mechanism could be quite different from insulation per se.

We thank the reviewer for pointing this out. Upon its discovery, CTCF was initially considered to be a repressor based on reporter gene assays. In the meantime, it turned out that CTCF through its interaction with cohesin functions as an insulator or architectural protein. However, it is important to note that a recent paper, using acute degradation of CTCF combined with nascent transcript analysis, has reinforced the notion that CTCF can also function as a transcriptional repressor in a manner independent of its architectural function (Luan et al., Nat. Struct. Mol. Biol. 29, 1136-1144). In response to the reviewer’s comment, we have performed transient transfection experiments with wild-type and mutant PAIR4-luciferase constructs in established pro-B and pre-B cells. The data of these experiment are shown in Figure EV5I and indicate that the CTCF bound to its binding site in PAIR4 can function as a transcriptional repressor. We mention these new data in the result and discussion on page 16 and 25 (top), respectively.

2nd Revision - Editorial Decision 18th May 2023

Hi Meinrad,

Thank you for submitting your revised manuscript to The EMBO Journal. Your manuscript was initially handled by Stefanie Boehm, but as Stefanie has left the journal, I am stepping in as secondary editor on the manuscript.

Your manuscript has now been re-reviewed by the original referees. As you can see from the comments below, the referees appreciate the introduced changes and support publication here. I am therefore pleased to let you know that we will proceed with the acceptance of the manuscript for publication here. Before sending you the formal acceptance letter there are a few editorial comments that need to be resolved.

When you submit the revised version please also submit a point-by-point response.

Editorial points:

- Please make sure that the funding information is also provided in the online submission system (Boehringer Ingelheim).

- You can only have 5 keywords.

- COI needs be renamed to Disclosure and competing interests

- Please remove the Authors Contributions from the manuscript. The 'Author Contributions' section is replaced by the CRediT contributor roles taxonomy to specify the contributions of each author in the journal submission system. Please use the free text box in the 'author information' section of the manuscript submisssion system to provide more detailed descriptions (e.g., 'X provided intracellular Ca++ measurements in fig Y')

- Tables EV1-EV3 should be renamed to Datasets EV1 etc and the callouts in the manuscript should be corrected accordingly

- You can only have 5 EV figures and Figures EV6 and EV7 need to be moved to an Appendix file with a ToC, and renamed "Appendix Figure S1" ,etc. Their callouts in the manuscript should also be corrected.

- The synopsis image should be provided as jpeg, TIFF or png format and size should be 550 pixels wide x 200-400 pixels high.

- The source data for the EV figures should be zipped in one file labelled Source Data EV figures.

- Our publisher has also done their pre-publication check on your manuscript. When you log into the manuscript submission system you will see the file "Data Edited Manuscript file". Please take a look at the word file and the comments regarding the figure legends and respond to the issues.

Congratulations on a nice study!

best Karin

Karin Dumstrei, PhD
Senior Editor
The EMBO Journal

Instructions for preparing your revised manuscript:

Guide For Authors: https://www.embopress.org/page/journal/14602075/authorguide

----

Referee #1:

The manusucript has further improved from the original submission, which already was outstanding. I recommend rapid

publication of these important findings.

Referee #2:

The authors have very thoroughly addressed all of my comments. The original submission already described an excellent study and the current version is further improved. I feel the conclusions of the paper are justified based on presented data and that no further experiments need to be done . I also feel that no further revisions of the text are needed. I support publication of this very interesting study in the EMBO J with high priority.

Referee #3:

I am satisfied with the responses to my comments and I am supportive of publication without further revision.

Point-by-point response to the editorial points of manuscript EMBOJ-2022-112741R1

Dear Karin,

Our response to the editorial comments is shown below in blue color.

- Please make sure that the funding information is also provided in the online submission system (Boehringer Ingelheim).

Boehringer Ingelheim is the pharmaceutical company that supports the IMP in general but not in a project-specific manner. Hence, we cannot enter Boehringer Ingelheim in the online submission system.

- You can only have 5 keywords.

We reduced the keywords to 5: *Igh* V_H gene recombination, PAIR4 element, novel recombination enhancers, CTCF, Pax5

- COI needs be renamed to Disclosure and competing interests

We have changed the COI title as requested on page 38.

- Please remove the Authors Contributions from the manuscript. The 'Author Contributions' section is replaced by the CRediT contributor roles taxonomy to specify the contributions of each author in the journal submission system. Please use the free text box in the 'author information' section of the manuscript submisssion system to provide more detailed descriptions (e.g., 'X provided intracellular Ca++ measurements in fig Y')

We have removed Authors Contributions. The author contribution text was added in the free text box already during the submission of the revised manuscript.

- Tables EV1-EV3 should be renamed to Datasets EV1 etc and the callouts in the manuscript should be corrected accordingly.

We have changed Tables to Datasets in the description of the Excel files and throughout the manuscript.

- You can only have 5 EV figures and Figures EV6 and EV7 need to be moved to an Appendix file with a ToC, and renamed "Appendix Figure S1" etc. Their callouts in the manuscript should also be corrected.

We have decided to rearrange the data of the EV figures so that they now fit into 5 EV figures, which we have uploaded. We accordingly changed the figure legends and figure callouts in the manuscript.

- The synopsis image should be provided as jpeg, TIFF or png format and size should be 550 pixels wide x 200-400 pixels high.

We now provide the synopsis image as a png file, as requested.

- The source data for the EV figures should be zipped in one file labelled Source Data EV figures.

We have submitted the source data for the EV figures as one file, as requested.

- Our publisher has also done their pre-publication check on your manuscript. When you log into the manuscript submission system you will see the file "Data Edited Manuscript file". Please take a look at the word file and the comments regarding the figure legends and respond to the issues.

We have responded to comments of the pre-publication check by making the corresponding changes in the figure legends.

With best regards,

Meinrad Busslinger

3rd Revision - Editorial Decision 2nd Jun 2023

Dear Meinrad,

Thank you for submitting your manuscript to The EMBO Journal. I have now looked carefully at everything and all looks fine. I am therefore very pleased to accept the manuscript for publication here.

with best wishes

Karin

Karin Dumstrei, PhD
Senior Editor
The EMBO Journal

Please note that it is EMBO Journal policy for the transcript of the editorial process (containing referee reports and your response letter) to be published as an online supplement to each paper. If you do NOT want this, you will need to inform the Editorial Office via email immediately. More information is available here:
https://www.embopress.org/page/journal/14602075/authorguide#transparentprocess

Your manuscript will be processed for publication in the journal by EMBO Press. Manuscripts in the PDF and electronic editions of The EMBO Journal will be copy edited, and you will be provided with page proofs prior to publication. Please note that supplementary information is not included in the proofs.

You will be contacted by Wiley Author Services to complete licensing and payment information. The required 'Page Charges Authorization Form' is available here: https://www.embopress.org/pb-assets/embo-site/tej_apc.pdf - please download and complete the form and return to embopressproduction@wiley.com

EMBO Press participates in many Publish and Read agreements that allow authors to publish Open Access with reduced/no publication charges. Check your eligibility: https://authorservices.wiley.com/author-resources/Journal-Authors/open-access/affiliation-policies-payments/index.html

Should you be planning a Press Release on your article, please get in contact with embojournal@wiley.com as early as possible, in order to coordinate publication and release dates.

If you have any questions, please do not hesitate to call or email the Editorial Office. Thank you for your contribution to The EMBO Journal.

EMBO Press Author Checklist

Corresponding Author Name: Meinrad Busslinger	
Journal Submitted to: EMBO Journal	
Manuscript Number: EMBOJ-2022-112741	

USEFUL LINKS FOR COMPLETING THIS FORM
The EMBO Journal - Author Guidelines
EMBO Reports - Author Guidelines
Molecular Systems Biology - Author Guidelines
EMBO Molecular Medicine - Author Guidelines

Reporting Checklist for Life Science Articles (updated January
This checklist is adapted from Materials Design Analysis Reporting (MDAR) Checklist for Authors. MDAR establishes a minimum set of requirements in transparent reporting in the life sciences (see Statement of Task: 10.31222/osf.io/9sm4x). Please follow the journal's guidelines in preparing your
Please note that a copy of this checklist will be published alongside your article.

Abridged guidelines for figures
1. Data
The data shown in figures should satisfy the following conditions:
- the data were obtained and processed according to the field's best practice and are presented to reflect the results of the experiments in an accurate and unbiased manner.
- ideally, figure panels should include only measurements that are directly comparable to each other and obtained with the same assay.
- plots include clearly labeled error bars for independent experiments and sample sizes. Unless justified, error bars should not be shown for technical
- if n<5, the individual data points from each experiment should be plotted. Any statistical test employed should be justified.
- Source Data should be included to report the data underlying figures according to the guidelines set out in the authorship guidelines on Data

2. Captions
Each figure caption should contain the following information, for each panel where they are relevant:
- a specification of the experimental system investigated (eg cell line, species name).
- the assay(s) and method(s) used to carry out the reported observations and measurements.
- an explicit mention of the biological and chemical entity(ies) that are being measured.
- an explicit mention of the biological and chemical entity(ies) that are altered/varied/perturbed in a controlled manner.
- the exact sample size (n) for each experimental group/condition, given as a number, not a range;
- a description of the sample collection allowing the reader to understand whether the samples represent technical or biological replicates (including how many animals, litters, cultures, etc.).
- a statement of how many times the experiment shown was independently replicated in the laboratory.
- definitions of statistical methods and measures:
  - common tests, such as t-test (please specify whether paired vs. unpaired), simple χ2 tests, Wilcoxon and Mann-Whitney tests, can be unambiguously identified by name only, but more complex techniques should be described in the methods section;
  - are tests one-sided or two-sided?
  - are there adjustments for multiple comparisons?
  - exact statistical test results, e.g., P values = x but not P values < x;
  - definition of 'center values' as median or average;
  - definition of error bars as s.d. or s.e.m.

Please complete ALL of the questions below.
Select "Not Applicable" only when the requested information is not relevant for your study.

Materials

Newly Created Materials	Information included in the manuscript?	In which section is the information available? (Reagents and Tools Table, Materials and Methods, Figures, Data Availability Section)
New materials and reagents need to be available; do any restrictions apply?	Not Applicable	

Antibodies	Information included in the manuscript?	In which section is the information available? (Reagents and Tools Table, Materials and Methods, Figures, Data Availability Section)
For **antibodies** provide the following information:<br>- Commercial antibodies: RRID (if possible) or supplier name, catalogue number and or/clone number<br>- Non-commercial: RRID or citation	Yes	Materials and Methods

DNA and RNA sequences	Information included in the manuscript?	In which section is the information available? (Reagents and Tools Table, Materials and Methods, Figures, Data Availability Section)
**Short novel DNA or RNA including primers, probes**: provide the sequences.	Yes	Table EV2

Cell materials	Information included in the manuscript?	In which section is the information available? (Reagents and Tools Table, Materials and Methods, Figures, Data Availability Section)
**Cell lines:** Provide species information, strain. Provide accession number in repository **OR** supplier name, catalog number, clone number, and/**OR** RRID.	Yes	Materials and Methods, Figure legends
**Primary cultures:** Provide species, strain, sex of origin, genetic modification status.	Yes	Materials and Methods, Figure legends
Report if the cell lines were recently **authenticated** (e.g., by STR profiling) and tested for mycoplasma contamination.	Not Applicable	

Experimental animals	Information included in the manuscript?	In which section is the information available? (Reagents and Tools Table, Materials and Methods, Figures, Data Availability Section)
**Laboratory animals or Model organisms:** Provide species, strain, sex, age, genetic modification status. Provide accession number in repository **OR** supplier name, catalog number, clone number, **OR** RRID.	Yes	Materials and Methods, Figure legends
**Animal observed in or captured from the field:** Provide species, sex, and age where possible.	Not Applicable	
Please detail **housing and husbandry conditions**.	Yes	Materials and Methods

Plants and microbes	Information included in the manuscript?	In which section is the information available? (Reagents and Tools Table, Materials and Methods, Figures, Data Availability Section)
**Plants:** provide species and strain, ecotype and cultivar where relevant, unique accession number if available, and source (including location for collected wild specimens).	Not Applicable	
**Microbes:** provide species and strain, unique accession number if available, and source.	Not Applicable	

Human research participants	Information included in the manuscript?	In which section is the information available? (Reagents and Tools Table, Materials and Methods, Figures, Data Availability Section)
If collected and within the bounds of privacy constraints report on age, sex and gender or ethnicity for all study participants.	Not Applicable	

Core facilities	Information included in the manuscript?	In which section is the information available? (Reagents and Tools Table, Materials and Methods, Figures, Data Availability Section)
If your work benefited from core facilities, was their service mentioned in the acknowledgments section?	Yes	Acknowledgements

Design

Study protocol	Information included in the manuscript?	In which section is the information available? (Reagents and Tools Table, Materials and Methods, Figures, Data Availability Section)

If study protocol has been **pre-registered, provide DOI in the manuscript**. For clinical trials, provide the trial registration number **OR** cite DOI.	Not Applicable	
Report the **clinical trial registration number** (at ClinicalTrials.gov or equivalent), where applicable.	Not Applicable	

Laboratory protocol	Information included in the manuscript?	In which section is the information available? (Reagents and Tools Table, Materials and Methods, Figures, Data Availability Section)
Provide DOI OR other citation details if **external detailed step-by-step protocols** are available.	Yes	Materials and Methods

Experimental study design and statistics	Information included in the manuscript?	In which section is the information available? (Reagents and Tools Table, Materials and Methods, Figures, Data Availability Section)
Include a statement about **sample size** estimate even if no statistical methods were used.	Yes	Figure legends, Materials and Methods
Were any steps taken to minimize the effects of subjective bias when allocating animals/samples to treatment (e.g. **randomization procedure**)? If yes, have they been described?	Not Applicable	
Include a statement about **blinding** even if no blinding was done.	Not Applicable	
Describe **inclusion/exclusion criteria** if samples or animals were excluded from the analysis. Were the criteria pre-established?<br><br>If sample or data points were omitted from analysis, report if this was due to attrition or intentional exclusion and provide justification.	Not Applicable	
For every figure, are **statistical tests** justified as appropriate? Do the data meet the assumptions of the tests (e.g., normal distribution)? Describe any methods used to assess it. Is there an estimate of variation within each group of data? Is the variance similar between the groups that are being statistically compared?	Yes	Materials and Methods, Figure legends

Sample definition and in-laboratory replication	Information included in the manuscript?	In which section is the information available? (Reagents and Tools Table, Materials and Methods, Figures, Data Availability Section)
In the figure legends: state number of times the experiment was **replicated** in laboratory.	Yes	Figure legends, Source data
In the figure legends: define whether data describe **technical or biological replicates**.	Yes	Figure legends, Source data

Ethics

Ethics	Information included in the manuscript?	In which section is the information available? (Reagents and Tools Table, Materials and Methods, Figures, Data Availability Section)
Studies involving **human participants**: State details of **authority granting ethics approval** (IRB or equivalent committee(s), provide reference number for approval.	Not Applicable	
Studies involving **human participants**: Include a statement confirming that **informed consent** was obtained from all subjects and that the experiments conformed to the principles set out in the WMA Declaration of Helsinki and the Department of Health and Human Services Belmont Report.	Not Applicable	
Studies involving **human participants:** For publication of **patient photos**, include a statement confirming that consent to publish was obtained.	Not Applicable	
Studies involving experimental **animals**: State details of **authority granting ethics approval** (IRB or equivalent committee(s), provide reference number for approval. Include a statement of compliance with ethical regulations.	Yes	Materials and Methods
Studies involving **specimen and field samples:** State if relevant **permits** obtained, provide details of authority approving study; if none were required, explain why.	Not Applicable	

Dual Use Research of Concern (DURC)	Information included in the manuscript?	In which section is the information available? (Reagents and Tools Table, Materials and Methods, Figures, Data Availability Section)
Could your study fall under dual use research restrictions? Please check biosecurity documents and list of **select agents and toxins** (CDC): https://www.selectagents.gov/sat/list.htm	Not Applicable	
If you used a select agent, is the security level of the lab appropriate and reported in the manuscript?	Not Applicable	
If a study is subject to dual use research of concern regulations, is the name of the **authority granting approval and reference number** for the regulatory approval provided in the manuscript?	Not Applicable	

Reporting

The MDAR framework recommends adoption of discipline-specific guidelines, established and endorsed through community initiatives. Journals have their own policy about requiring specific guidelines and recommendations to complement MDAR.

Adherence to community standards	Information included in the manuscript?	In which section is the information available? (Reagents and Tools Table, Materials and Methods, Figures, Data Availability Section)
State if relevant guidelines or checklists (e.g., **ICMJE, MIBBI, ARRIVE, PRISMA**) have been followed or provided.	Not Applicable	
For **tumor marker prognostic studies,** we recommend that you follow the **REMARK** reporting guidelines (see link list at top right). See author guidelines, under 'Reporting Guidelines'. Please confirm you have followed these guidelines.	Not Applicable	
For **phase II and III randomized controlled trials**, please refer to the **CONSORT** flow diagram (see link list at top right) and submit the CONSORT checklist (see link list at top right) with your submission. See author guidelines, under 'Reporting Guidelines'. Please confirm you have submitted this list.	Not Applicable	

Data Availability

Data availability	Information included in the manuscript?	In which section is the information available? (Reagents and Tools Table, Materials and Methods, Figures, Data Availability Section)
Have **primary datasets** been deposited according to the journal's guidelines (see 'Data Deposition' section) and the respective accession numbers provided in the Data Availability Section?	Yes	Materials and Methods, Table EV3
Were **human clinical and genomic datasets** deposited in a public access-controlled repository in accordance to ethical obligations to the patients and to the applicable consent agreement?	Not Applicable	
Are **computational models** that are central and integral to a study available without restrictions in a machine-readable form? Were the relevant accession numbers or links provided?	Not Applicable	
If publicly available data were reused, provide the respective **data citations in the reference list**.	Yes	Materials and Methods, Table EV3
